# Comparison of Odor Mitigation in Squid Cartilage Fermented by *Saccharomyces cerevisiae* and *Lactobacillus plantarum*

**DOI:** 10.3390/foods14173117

**Published:** 2025-09-06

**Authors:** Tingting Zhang, Rongbin Zhong, Feifei Shi, Qian Yang, Peng Liang, Jiacong Deng

**Affiliations:** 1 College of Food Science, Fujian Agriculture and Forestry University, Fuzhou 350002, China; 2 College of Oceanology and Food Science, Quanzhou Normal University, Quanzhou 362000, China; 3 College of Food and Biological Engineering, Fujian Polytechnic Normal University, Fuzhou 350300, China

**Keywords:** Biological fermentation technology, squid cartilage, volatile flavor substances, *Saccharomyces cerevisiae*, *Lactobacillus plantarum*

## Abstract

This study established a biological fermentation process using *Saccharomyces cerevisiae* and *Lactobacillus plantarum* to deodorize squid cartilage homogenate. The optimal fermentation conditions for *S. cerevisiae* were determined as follows: fermentation time 105 min, temperature 34 °C, and inoculum size 0.85%. For *L. plantarum*, the optimum conditions were 79 min, 34.5 °C, and 4.5% inoculum. Based on electronic nose and HS-SPME-GC-MS analyses, *S. cerevisiae* outperformed *L. plantarum* in eliminating key offensive odor compounds, especially sulfur-containing compounds and aldehydes, while promoting the formation of pleasant aroma compounds such as esters and ketones (e.g., carvone and δ-pentenol). Mechanistic insights suggest that the enhanced deodorization efficiency of *S. cerevisiae* may be attributed to its multi-pathway synergistic metabolism, involving enzymes like dioxygenases and sulfide oxidases that facilitate the conversion of malodorous substances into odorless or pleasantly aromatic compounds. These findings provide a valuable theoretical and practical foundation for the high-value utilization of squid processing by-products and propose a promising bio-deodorization strategy for aquatic products.

## 1. Introduction

Squid is a prominent species of marine cephalopod, possessing a number of advantageous biological characteristics. These include a brief life cycle, rapid growth rates, high energy conversion efficiency, and robust reproductive capacity [1]. Its stock biomass accounts for approximately 36% of the total global marine organisms [2], and the annual average catch in the last decade reached 4.3 million tons, accounting for 5% of the total global fish and cephalopod catch, occupying an important position in the marine fishery economy [3]. However, the environmental challenges associated with the squid processing industry are increasingly evident. The processing of squid generates approximately 40–50% of by-products, primarily comprising the head, fins, feet, viscera, and underutilized outer coat and cartilage tissues. These by-products are currently primarily utilized as low-value feedstuffs, and their efficient utilization has not been achieved, resulting in significant organic pollution and the wastage of high-quality biomass resources [4]. It is important to note that squid cartilage, as a typical processing by-product, is rich in collagen, chondroitin sulfate and other bioactive components, despite accounting for only 2% of the total mass of the raw material [5]. The prevailing processing system predominantly treats it as a by-product, and this rudimentary disposal method not only exacerbates environmental concerns but also results in a substantial loss of valuable biological resources.

In recent years, major breakthroughs in biotechnological extraction techniques have advanced the utilization of high-value squid cartilage resources, demonstrating the material’s immense potential: For example, Leng, Wang [4] confirmed that type II collagen derived from squid laryngeal cartilage promotes healing of osteoporotic fractures. Tan’s team [6] used wet spinning technology to turn cartilage chitin into absorbable surgical sutures, and Zhao, Li [7] further elucidated the efficacy of type II collagen peptides in alleviating osteoarthritis. However, these applications are limited by characteristic fishy residues, such as thiols, unsaturated aldehydes, and pyrazine compounds, which form stable complexes with collagen matrices via hydrophobic interactions and disulfide bonds [8]. The root cause of this issue lies in the unique nature of squid cartilage extracts. Studies have confirmed their strong, characteristic fishy odor, which is primarily attributed to residual lipids and trimethylamine oxide (TMAO) degradation products. The issue of a fishy odor in aquatic collagen is particularly pronounced in cephalopod cartilage [9], and squid by-products have a significantly stronger fishy odor than fish because they are rich in phospholipids and TMAO, which makes them harder to eliminate [10]. Therefore, developing efficient deodorization technology is essential for converting squid cartilage into a valuable resource.

Currently, fishy flavor removal techniques for aquatic products are mainly divided into three categories: physical, chemical and biological methods [11]. Physical methods such as activated carbon adsorption or β-cyclodextrin embedding are easy to operate and have no chemical residues, but the removal rate of bound fishy components is low, and it is easy to damage the texture of the product [12]; chemical methods such as acid and alkali treatment or oxidant deodorization can improve the efficiency, but there is a risk of protein denaturation, flavor deterioration, and the retention of harmful by-products [13,14]. In comparison with conventional methodologies, the biocatalytic conversion mechanism of biotechnology exhibits distinctive advantages in the removal of fishy odors. Pan, Jia [15] discovered that the fermentation process of *S. cerevisiae* can enhance the off-flavor of pufferfish skin gel and mitigate the intensity of fishy odors. As indicated in the study by Li’s team [12], *S. cerevisiae* fermentation has been demonstrated to be an effective method for the removal of fishy odors from tilapia enzymatic hydrolysates. Ma, Liang [16] further elucidated the effect of *L. plantarum* deodorization metabolic mechanism in tuna and found that microbial fermentation produced pleasant flavor substances while removing unpleasant odors. Therefore, the bio-deodorization technique, which specifically degrades fishy precursor substances by microbial or enzymatic agents, not only has a higher deodorization rate, but also improves the flavor profile [16,17].

This study aims to compare the effectiveness of *S. cerevisiae* and *L. plantarum* in removing fishy odors. In this study, squid cartilage was utilized as the raw material, and was separately inoculated with *S. cerevisiae* and *L. plantarum* for fermentation. Using sensory evaluation as the standard, the fermentation process conditions (i.e., fermentation time, fermentation temperature, and inoculum size) were optimized. Subsequently, the squid cartilage was fermented using the optimal fermentation process conditions for the two strains. The volatile flavor compounds in the fermentation products were analyzed using electronic nose technology and headspace solid-phase microextraction gas chromatography-mass spectrometry (HS-SPME-GC-MS). Finally, by comparing the abundance differences in volatile flavor compounds, key fishy odor compounds were identified, and conclusions were drawn regarding the fishy odor removal effects of *S. cerevisiae* and *L. plantarum*.

## 2. Materials and Methods

### 2.1. Materials

Squid cartilage obtained from Fuzhou Hongdong Food Co., Ltd. (Fuzhou, China). The commercial baker’s yeast (*S*. *cerevisiae*) was purchased from Angel Yeast Co., Ltd. (Yichang, China). *L. plantarum* was obtained from China Industrial Microbial Strain Preservation and Management Centre (Beijing, China), strain no.: CICC 10345. MRS medium and MRS broth medium were purchased from Huasheng Chemical Reagent Co., Ltd. (Tianjin, China). NaCl and sucrose are both food grade and come from Fujian Salt Industry Group Co., Ltd. (Fuzhou, China) and Xiamen Gulong Food Co., Ltd. (Xiamen, China), respectively. Other chemical reagents are analytical grade or chromatographic grade.

### 2.2. Reactivation of S. cerevisiae and L. plantarum

Prior to inoculation for fermentation, 2% *S. cerevisiae* was rehydrated in activation solution containing 4% sterilized sucrose at 37 °C for 30 min. Then, the rehydrated activation solution of *S. cerevisiae* was added to the liquid medium containing 2.5% NaCl and 5% sucrose at 10% of the inoculation volume. Incubate the mixture on a shaking incubator at 180 rpm and 28 °C for 12 h to obtain activated *S. cerevisiae* for later fermentation [18]. After activation, *S. cerevisiae* were collected by centrifugation (4000× *g*, 10 min, 4 °C) and resuspended in sterile physiological saline (0.85% NaCl). Cell concentration was determined by measuring the optical density (OD_600_) at 600 nm using a spectrophotometer, and calibrated using a standard curve of viable cell counts established by counting on YPD medium. The cell suspension was then adjusted to a final concentration of 1 × 10^8^ CFU/mL using sterile physiological saline for fermentation experiments [19]. All fermentation experiments were conducted in 250 mL Erlenmeyer Flask, each containing 100 mL of squid cartilage homogenate.

The method for reviving *L. plantarum* was based on the method described in previous studies [20]. In brief, 0.5 mL of sterile physiological saline was added to an ampoule containing dried *L. plantarum* powder. The *L. plantarum* solution was then transferred to sterile MRS medium. The mixture was cultured at 37 °C for 24 h to obtain the revived bacterial suspension. Take 100 μL of the revived bacterial suspension and inoculate it into 5 mL of MRS medium, incubating at 37 °C for 18 h. Finally, transfer the suspension at a 2% inoculum rate to 50 mL of MRS medium and incubate anaerobically at 37 °C for 12 h to obtain the activated *L. plantarum* solution, which is used for subsequent fermentation. Activated *L. plantarum* was collected by centrifugation (8000× *g*, 10 min, 4 °C) and resuspended in sterile saline. The cell concentration was adjusted based on the optical density (OD_600_) at 600 nm and verified by counting on MRS agar plates to obtain the standard suspension concentration of 1 × 10^9^ CFU/mL required for fermentation experiments. All fermentation experiments were conducted in 250 mL Erlenmeyer Flask, each containing 100 mL of squid cartilage homogenate.

### 2.3. Single-Factor Experiment

Prior to statistical optimization, single-factor experiments were conducted to assess the individual effects of each variable (fermentation time, temperature, and addition amount) on deodorization effectiveness [21]. The purpose of this preliminary study was to determine the appropriate range for each variable in subsequent response surface methodology studies.

After removing the squid meat, the squid cartilage is washed and drained. The squid cartilage was mixed with deionized water at a ratio of 1:10 (weight/volume) and homogenized for 1 min at a speed of 10,000 rpm. Add the standardized *S. cerevisiae* or *L. plantarum* cell suspension to the squid cartilage homogenate at the specified addition ratio (Note: The percentage here refers to the ratio of the volume of the standardized suspension added to 100 mL of homogenate. Sensory evaluation was used as the assessment criterion to evaluate the effects of fermentation time, fermentation temperature, and the addition ratio (volume ratio of microbial fermentation solution to squid cartilage homogenate) for each strain on the removal of fishy odor. The fermentation time for *S. cerevisiae* was set at 30, 60, 90, 120, and 150 min, and the fermentation temperature was set at 25, 30, 35, 40, and 45 °C. The addition ratios were set at 0.2%, 0.4%, 0.6%, 0.8%, and 1.0%. The fermentation products of *S. cerevisiae* were named the Y group. The fermentation time for *L. plantarum* was set at 30, 60, 90, 120, and 150 min, with fermentation temperatures of 25, 30, 35, 40, and 45 °C, respectively, and addition rates of 2%, 4%, 6%, 8%, and 10%, respectively. The fermentation products of *L. plantarum* were named the L group. The unfermented squid cartilage homogenate was named the CK group.

### 2.4. Response Surface Experiment

The subsequent single-factor experiment yielded the results that informed the selection of a response surface experiment. This experiment was designed using Design-Expert 13 software, version 13.0.5.0 (2021) (Stat-Ease Inc.,Minneapolis, MN, USA), which created a Box–Behnken design. The independent variables in the design were divided into three levels, coded as −1, 0, and 1, as illustrated in Table 1 and Table 2. The dependent variable that was the subject of measurement was the sensory score. Table 3 and Table 4 presents the levels of the independent variables and the design matrix. The experimental design comprised a total of 17 runs, including five replicates at the center point, and was conducted in a random order to minimize unpredictable variations. Finally, verification experiments were conducted.

### 2.5. Sensory Evaluation

As stated in the previous literature [18], a group consisting of five males and five females underwent training over a two-month period to become acquainted with the odors of fishiness, floral, sweet, earthy, and fatty. The group members were randomly assigned to perform sensory evaluations of the fishiness of the fermented samples. Following each sniff, a 20-second interval was observed, during which the subject was exposed to fresh air. Each evaluator was tasked with the evaluation of each sample on three separate occasions. The intensity of the fishy odor was evaluated on a scale ranging from 0 to 5, where 0 indicated a strong fishy odor and 5 indicated an absence of fishy odor.

### 2.6. Electronic Nose Analysis

Electronic nose analysis was conducted referring to the methods as previously described with slight modification [22]. The product sample (10 g) was placed within the headspace bottle, and sealed with a silica gel spacer. The bottle was left at room temperature for 2 h. Following this, the electronic nose sampling needle was inserted into the bottle, and the headspace air was extracted for analysis. The electronic nose determination parameters were as follows: a sampling time interval of 1 s, a pre-sampling time of 5 s, a self-cleaning time of 90 s, an inlet flow rate of 400 mL/min, and a sample determination time of 150 s. The data between 141 and 143 s after stabilization were selected for flavor component analysis.

Each sensor of electronic nose responds preferentially to specific organic compounds: W1C for aromatic benzene, W5S for nitrogen oxides, W3C for ammonia, W6S for hydrocarbons, W5C for short-chain alkanes, W1S for wide-chain alkanes, W1W for sulfur-inorganics, W2S for alcohols, aldehydes and ketones, W2W for aromatic compounds, W3S is for detection of long-chain alkanes.

### 2.7. HS-SPME-GC/MS Analysis

#### 2.7.1. Sample Preparation and Extraction

The material was weighed post-harvest, immediately frozen in liquid nitrogen and stored at −80 °C until required. The samples were then ground to a powder in liquid nitrogen.

Thereafter, 0.2 g (0.2 mL of the powder was transferred into a 20 mL headspace vial (Agilent, Palo Alto, CA, USA) containing 0.2 g of NaCl powder to inhibit any enzymatic reaction. The vials were then sealed using a rolled-edge cap with a TFE silicone headspace septum (Agilent). Under constant temperature conditions of 60 °C, shake for 5 min, insert a 120 µm DVB/CWR/PDMS extraction head into the headspace vial of the sample, perform headspace extraction for 15 min, desorb at 250 °C for 5 min, and then perform GC-MS separation and identification. Prior to sampling, the extraction head is aged at 250 °C for 5 min in the Fiber Conditioning Station.

#### 2.7.2. GC-MS Conditions

Following the sampling stage, the volatile organic compounds (VOCs) on the fiber coatings were desorbed at the inlet of a gas chromatograph (model 8890; Agilent) in splitless mode for a period of 5 min at a temperature of 250 °C. Identification and quantification of the VOCs was achieved through the utilization of an Agilent 8890 gas chromatograph, in conjunction with a 7000D mass spectrometer (Agilent), which was equipped with a 30 m × 0.25 mm × 0.25 μm DB-5MS (5% phenyl-polymethylsiloxane) capillary column. The carrier gas utilized was helium, with a linear velocity of 1.2 mL/min. The injector temperature was maintained at 250 °C. The oven temperature was initiated at 40 °C (3.5 min) and subsequently heated to 100 °C at a rate of 10 °C/min, 180 °C at 7 °C/min, and 280 °C at 25 °C/min for a duration of 5 min. The mass spectrum was recorded in 70 eV electron collision ionization mode. The temperatures of the quadrupole mass detector, the ion source and the transfer line were set to 150, 230 and 280 °C, respectively. The mass spectrum was subsequently utilized in selected ion monitoring (SIM) mode for the identification and quantification of the analytes. The internal standard is 3-Hexanone-2,2,4,4-d4, added at a concentration of 20 μL (10 μg/mL). The relative amount of volatile compounds in the sample (µg/g) was calculated using the following formula:

(1) $$X_{i}=\frac{V_{s}\times C_{s}}{V}\times \frac{I_{i}}{I_{s}}\times 10^{-3}$$

*X_i_* is the amount of compound i in the sample to be tested (μg/mL); *V_s_* is the volume of internal standard added (μL); *C_s_* is the concentration of internal standard (μg/mL); *V* is the volume of the sample to be tested (mL); *I_s_* is the peak area of the internal standard; *I_i_* is the peak area of compound i in the sample to be tested.

#### 2.7.3. Temperament Data Analysis

Additionally, the volatile flavor compounds were analyzed using the MyviCloud platform (https://cloud.metware.cn/, accessed on 26 August 2025). Unsupervised PCA (principal component analysis) was performed by the statistical function prcomp in R (www.r-project.org, accessed on 20 July 2025). Prior to the unsupervised PCA analysis, the data were scaled by unit variance. In the context of two-group analyses, the determination of differential metabolites was conducted through the utilization of the variable importance in projection (VIP) metric, with a threshold value of 1, in conjunction with the absolute Log_2_fold change (|Log_2_FC| ≥ 1.0). In the context of multi-group analyses, differential metabolites were determined by VIP (VIP > 1) and *p*-value (*p*-value < 0.05, ANOVA). The VIP values were extracted from the OPLS-DA results using the R package MetaboAnalystR (version 4.1.2), which also contains score plots and permutation plots. Prior to the implementation of the OPLS-DA, the data underwent log-transformation (log_2_) and mean centering. A permutation test (200 permutations) was performed to avoid overfitting.

### 2.8. ROAV Analysis

The relative odor activity value (ROAV) is a method for identifying the main flavor compounds in a food product by combining the sensory thresholds of the compounds. This method is utilized to elucidate the contribution of each flavor compound to the overall flavor profile of a given sample. Typically, an ROAV greater than 1 signifies that a compound exerts a direct influence on the flavor of a given sample [23,24]. The ROAV calculation of the odor compound was conducted using Equation (2):

(2) $$ROAV_{i}=\frac{C_{i}}{T_{i}}$$

where *ROAV_i_* is the relative odor activity value of the odor compound *i*, *C_i_* is the relative amount of the odor compound (μg/g or μg/mL), and *T_i_* is the threshold value of the odor compound (μg/g or μg/mL).

## 3. Results

This section may be divided by subheadings. It should provide a concise and precise description of the experimental results, their interpretation, as well as the experimental conclusions that can be drawn.

### 3.1. Optimization of the Fermentation Process for Removing Fishy Odor from Squid Cartilage Using S. cerevisiae

#### 3.1.1. Single-Factor Experimental Results and Analysis

**Figure 1:**
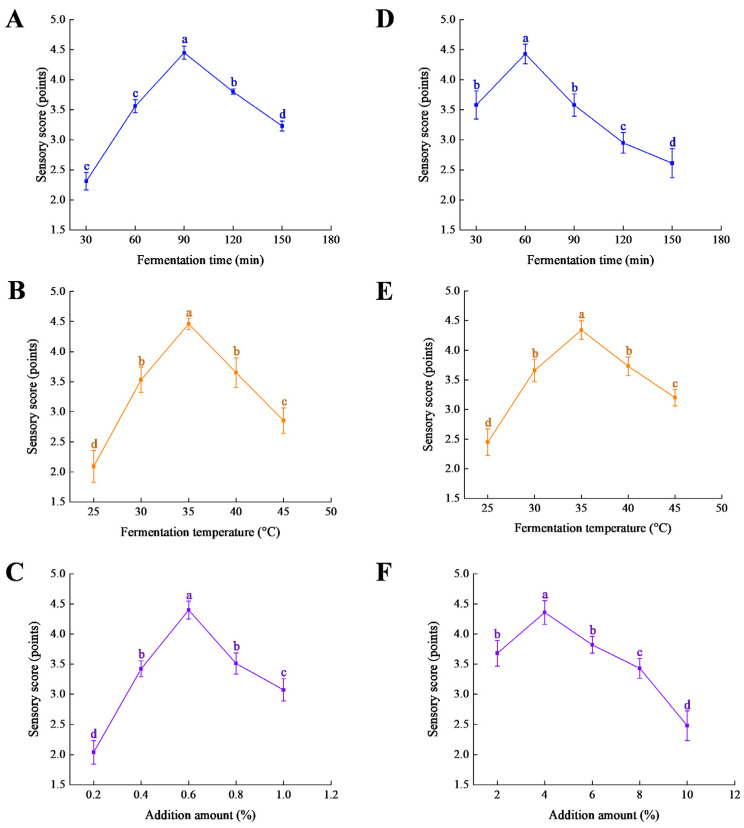
Effects of different factors on the deodorization effect of *S. cerevisiae* (**A**–**C**). Effects of different factors on the deodorization effect of *L. plantarum* (**D**–**F**).Significance letters (a, b, c, d) indicate significant differences between groups (*p* < 0.05).

Single-factor experiments showed that the deodorization efficiency of *S. cerevisiae* was significantly influenced by time, temperature, and addition amount (Figure 1A–C) In the time gradient, sensory scores peaked at 90 min, significantly higher than the scores at 30 min and 150 min. This indicates that moderately extending the fermentation time helps reduce fishy odor. After 90 min, a continuous decline in sensory scores was observed. Temperature experiments showed that optimal results were achieved at 35 °C, while both 25 °C and 45 °C significantly inhibited deodorization activity. Addition amount experiments indicated that 0.8% was the optimal concentration, with both lower and higher concentrations reducing efficiency.

#### 3.1.2. Response Surface Experiment Results and Analysis

Based on the results of the response surface experiment, fermentation time (A), fermentation temperature (B), and addition amount (C) were designated as independent variables. Sensory scores were used as the dependent variable for response surface optimization. The results of the response surface experiment are shown in Table 5. After comprehensive analysis of the experimental data set, the following second-order polynomial regression equation was obtained: R = 4.44 − 0.4000A − 0.2000B + 0.2250C + 0.2000AC + 0.1000BC − 0.8950A^2^ − 0.6950B^2^ − 0.6450C^2^. The data indicate that the regression model is highly significant (*p* < 0.0001), while the error term is not significant (*p* > 0.05). These results suggest that the model fits well and experimental errors are low. The coefficient of determination (R^2^), adjusted coefficient of determination (R^2^_Adj_), and coefficient of variation (CV) of the regression model are 0.9829, 0.9609, and 4.69%, respectively. This result indicates that the model explains over 98% of the variability in response values, enabling accurate analysis and prediction of experimental results. As shown in Table 5, the linear terms A, B, and C, as well as the quadratic terms A^2^, B^2^, and C^2^ in the model, all significantly influence the sensory scores (*p* < 0.05). Among the interaction terms, the AB and BC terms in the model are not significant, but the AC term significantly influences the sensory scores (*p* < 0.05). This finding indicates that fermentation time, fermentation temperature, and inoculum amount are significantly correlated with the enhancement of flavor in squid cartilage homogenate. Additionally, this study demonstrates that the overall flavor of squid cartilage homogenate is primarily influenced by fermentation time, followed by addition amount, and finally by fermentation temperature, as confirmed by the F-value.

**Figure 2:**
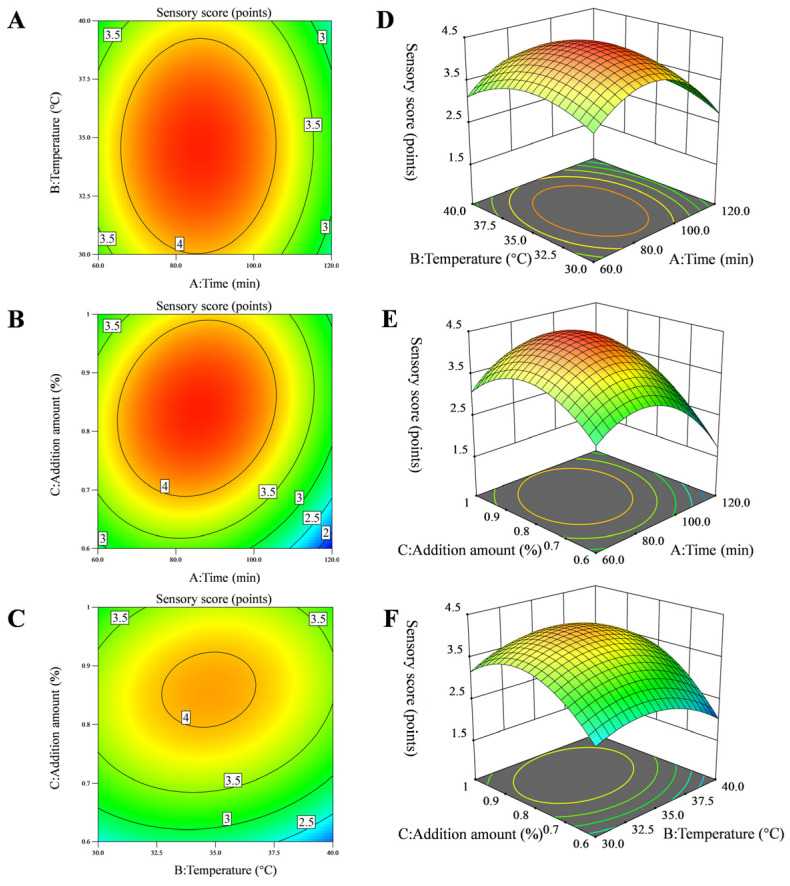
Contour plot (**A**–**C**) and 3D response surface plot (**D**–**F**) showing the effects of interactions between various factors on the deodorization effect of *S. cerevisiae*.

A response surface plot is a graphical representation of a regression equation. The shape of the curve in the plot intuitively reflects the influence of each factor on the corresponding value. The steeper the curve, the denser the contour lines, and the more elliptical the shape, the greater the influence of that factor on the response value. This indicates that the interaction between the two factors is more significant. Conversely, if the curve is gentler, the contour lines are sparser, and the shape is less elliptical, the interaction between the two factors is less significant [25]. Response surface plots reveal the maximum values, indicating that the selected factor levels are reasonable. As shown in Figure 2, the sensory scores in the model response surface plot exhibit a trend of first increasing and then decreasing with fermentation time, fermentation temperature, and addition amount. When fermentation temperature is fixed, the interaction between fermentation time and addition amount exhibits the steepest response surface at factors A (fermentation time) and C (addition amount), indicating that the AC interaction has the greatest influence on sensory scores. This result is consistent with the conclusions of the analysis of variance, which indicate that the AC interaction is statistically significant (*p* < 0.05). Based on the response surface experiment results, the optimized fermentation process for removing fishy odor from squid cartilage homogenate was determined as follows: fermentation time of 104.98 min, fermentation temperature of 34.37 °C, and addition amount of 0.85%. The predicted sensory score is 4.07 points. The model was validated, and considering practical operational constraints, the optimal process was adjusted to: fermentation time of 105 min, fermentation temperature of 34 °C, and addition amount of 0.85%. The sensory scores for the deodorized squid cartilage homogenate were all 4 points, closely matching the model’s predicted values. This indicates the reliability of the results.

### 3.2. Optimization of the Fermentation Process for Removing Fishy Odor from Squid Cartilage Using L. plantarum

#### 3.2.1. Single-Factor Experimental Results and Analysis

In single-factor experiments, *L. plantarum* exhibited different response pattern (Figure 1D–F). In the time gradient, the highest score was reached at 60 min, with a significant decline after 90 min, indicating a fast metabolic rate but weak persistence. Temperature experiments indicated that 35 °C is the optimal fermentation temperature. According to the product manual of the commercial brewing yeast strain used in this study (Angel Yeast Co., Ltd.), the recommended fermentation temperature range is 30–36 °C. This data is consistent with its recognized limited heat tolerance and the results observed in our experiments, where performance declined at temperatures above this range. In contrast, *L. plantarum* maintained significantly higher sensory scores at higher temperatures, demonstrating superior heat tolerance under the same conditions.

#### 3.2.2. Response Surface Experiment Results and Analysis

**Figure 3:**
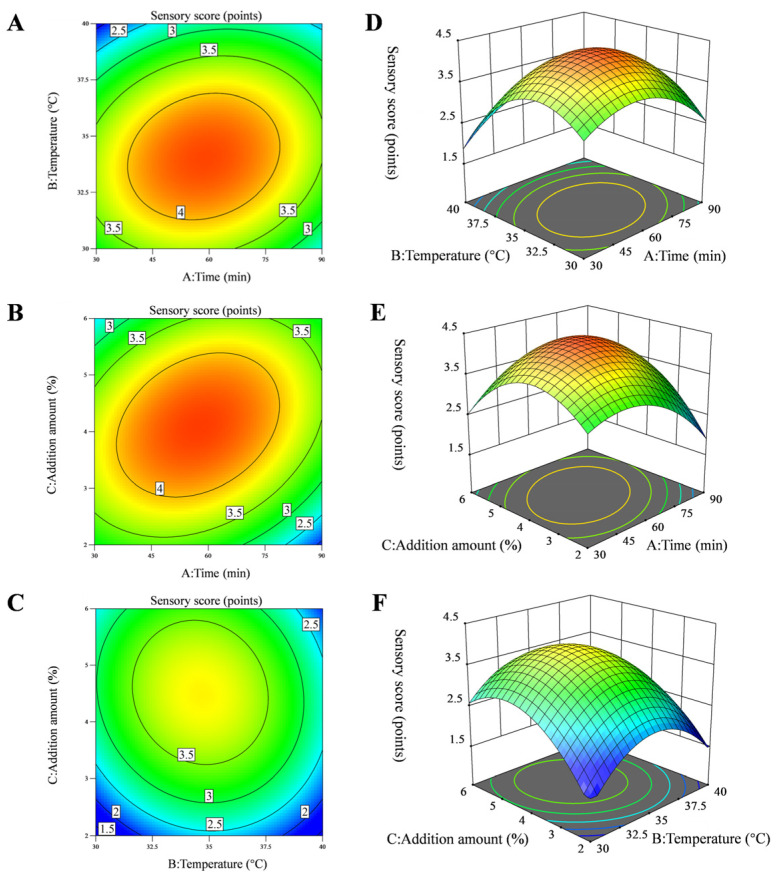
Contour plot (**A**–**C**) and 3D response surface plot (**D**–**F**) showing the effects of interactions between various factors on the deodorization effect of *L. plantarum*.

The results of the response surface experiment are shown in Table 6. After comprehensive analysis of the experimental data set, the following second-order polynomial regression equation was obtained: R = 4.20 − 0.2000A − 0.2875B + 0.1375C + 0.1250AB + 0.4250AC − 0.0500BC − 0.8000A^2^ − 0.9250B^2^ − 0.7250C^2^. A variance and significance analysis was conducted on this model. The data indicate that the regression model is highly significant (*p* < 0.0001), while the error term is not significant (*p* > 0.05). These results suggest that the model fits well and experimental error is low. The coefficient of determination (R^2^), adjusted coefficient of determination (R^2^_Adj_), and coefficient of variation (CV) of the regression model are 0.9847, 0.9650, and 5.23%, respectively. This result indicates that the model can explain over 98% of the variability in response values, enabling accurate analysis and prediction of experimental results. As shown in Figure 3 and Table 6, the linear terms A, B, and C, as well as the quadratic terms A^2^, B^2^, and C^2^ in both models, significantly influence the sensory scores (*p* < 0.05). Among the interaction terms, the AB and BC terms in both models are not significant, while the AC term has a highly significant influence on the sensory scores (*p* < 0.01). The results of this study indicate that fermentation time, fermentation temperature, and addition amount are significantly correlated with the enhancement of flavor in squid cartilage homogenate. In addition, the overall flavor of squid cartilage homogenate is mainly affected by fermentation temperature, followed by the fermentation time, and finally by addition amount. The sensory scores in the response surface plot exhibit a trend of first increasing and then decreasing with fermentation time, fermentation temperature, and addition amount. When the fermentation temperature is fixed, the interaction between fermentation time and addition amount exhibits the steepest response surface at factors A (fermentation time) and C (addition amount), indicating that the AC interaction has the greatest influence on sensory scores. This result is consistent with the conclusions of the analysis of variance, indicating that the AC interaction is statistically significant (*p* < 0.05). Based on the results of the response surface experiment, the optimized fermentation process for removing fishy odor from squid cartilage homogenate is determined as follows: fermentation time of 79.08 min, fermentation temperature of 34.40 °C, and addition rate of 4.57%. The predicted sensory score is 3.82 points. After model validation and consideration of practical operational constraints, the optimized process was adjusted to: fermentation time of 79 min, fermentation temperature of 34.5 °C, and addition amount of 4.5%. The sensory scores of the deodorized squid cartilage homogenate were all 4 points, highly consistent with the model-predicted values, which indicates the reliability of the predicted results.

### 3.3. Analysis of Electronic Nose Sensor Response

**Figure 4:**
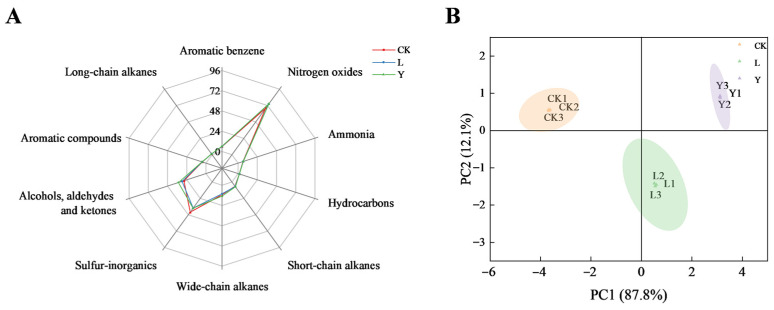
E-nose radargrams (**A**) and e-nose-based PCA 2D maps (**B**) for different treatment groups (CK, L, Y). The axes of the radar plot represent the intensity of each sensory attribute.

Electronic nose technology showed good capacity of gathering and distinguishing volatile odor compound present in sample by W1C, W5S, W3C, W6S, W5C, W1S, W1W, W2S, W2W and W3S sensor. As shown in Figure 4A, there is no significant difference between the CK, L, and Y groups in the e-nose response values of W1C, W3C, W6S, W5C, W1S, W2W and W3S sensors (sensitive to aromatic benzene, ammonia, hydrocarbons, short-chain alkanes, wide-chain alkanes, aromatic compounds and long-chain alkanes, respectively). However, compared with the CK group, both the L group (fermented by *L. plantarum*) and the Y group (fermented by *S. cerevisiae*) exhibited a marked decrease in the e-nose response value at W1W (sensitive to sulfur-inorganics). This phenomenon may be associated with the elevated activity of esterases and sulfur oxidases produced in the metabolism of the two strains. Such enzymes have been shown to be effective in reducing the production of fishy odor characteristics by catalyzing the oxidation or decomposition of sulfide precursors such as thiols [26]. Conversely, the L and Y groups have a higher response value to W5S (sensitive to NOx) and W2S (sensitive to alcohols, aldehydes and ketones) compared with the CK group, indicating that the addition of two exogenous strains may increase the concentration of NOx, alcohols, aldehydes and ketones in the overall compounds of squid cartilage.

Furthermore, the PCA was performed in the dimensionality reduction analysis of the sensor response data to understand the differences in sample’s odor pattern (Figure 4B). The results demonstrated that the first principal component (PC1, accounting for 87.8% of the total variance) and the second principal component (PC2, 12.1% of the total variance) collectively explained 99.9% of the observed variance (Figure 4B). In PCA space, the samples of the L and Y groups were significantly separated with the CK group along the PC1 axis, with the distribution of the Y group being the furthest away from the CK group, indicating that the fermentation treatment had the most significant change in the overall odor profile. PCA-based results demonstrated that both microorganisms significantly influenced the volatile odor components of squid cartilage. A subsequent analysis of the electronic nose response data demonstrated that both strains exhibited a substantial reduction in the concentration of sulfur-inorganics. However, a comparative analysis of the inter-strain differences indicated that *S. cerevisiae* exerted a more pronounced effect on increasing the concentration of nitrogen oxides and alcohols, aldehydes and ketones in comparison to *L. plantarum*. In order to achieve a comprehensive understanding of the mechanism of action, it is essential to analyze the specific volatile compound composition of the squid cartilage.

### 3.4. Analysis of Volatile Compound Composition

#### 3.4.1. Volatile Compound Phenotype Analysis

**Figure 5:**
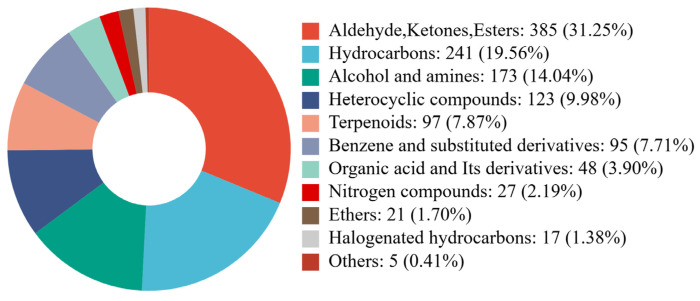
Circular plot of metabolite category composition. Each color in the color block is representative of a particular metabolite class, with the area of the block indicating the percentage of that class.

The volatile compounds present in squid cartilage homogenate were identified prior to and following fermentation, employing a technique known as HS-SPME-GC-MS analysis. A total of 1233 volatile compounds were identified from the CK, L, and Y groups (Supplementary Table S1). The circular plot of metabolite category composition (Figure 5) shows that it includes alcohols and amines (14.04%), aldehydes, ketones, and esters (31.25%), and benzene and its substituted derivatives (7.71%). The remaining compounds included ethers (1.7%), halogenated hydrocarbons (1.38%), heterocyclic compounds (9.98%), hydrocarbons (19.56%), nitrogen compounds (2.19%), organic acids and their derivatives (3.9%), others (0.41%), and terpenoid compounds (7.87%). The distinctive odor of squid cartilage is primarily attributable to its volatile compounds, with amines (e.g., trimethylamine) and aldehydes and ketones (e.g., lipid oxidation products hexanal and nonanal) constituting the predominant sources of this characteristic odor [27]. The detected components are closely related to the percentage of nitrogen compounds and aldehydes, ketones, and esters. Furthermore, the presence of nitrogen or sulfur-containing components in heterocyclic compounds has been demonstrated to induce the generation of noxious odors, often characterized by their putrid or fishy-like characteristics.

**Figure 6:**
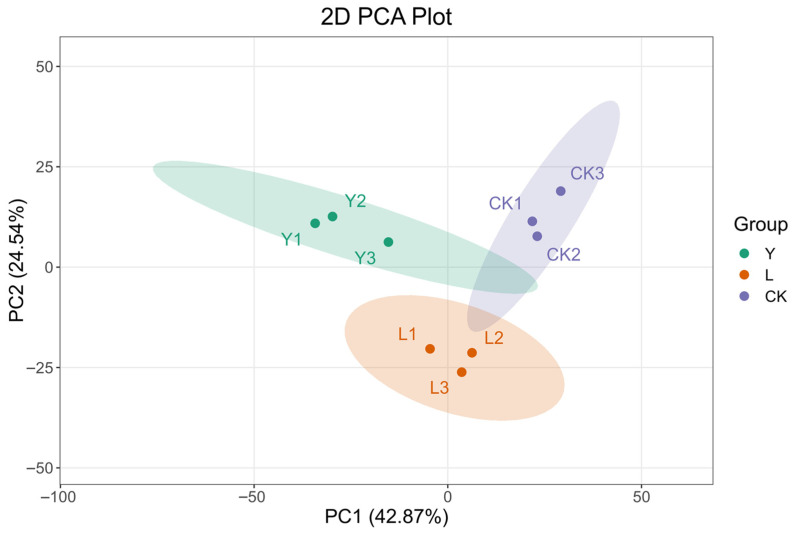
Plot of PCA scores of samples from different treatment groups (CK, L, Y).

**Figure 7:**
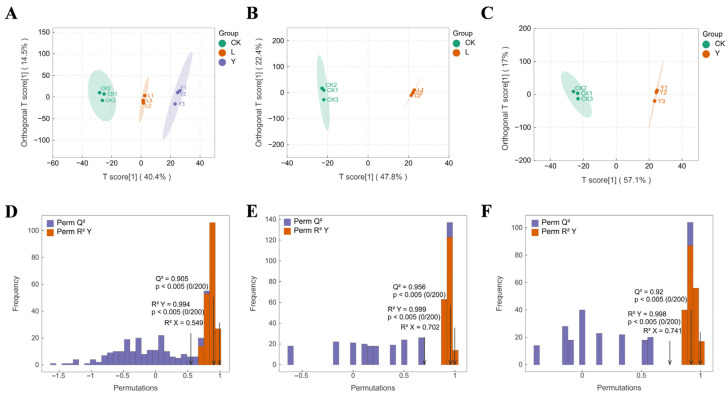
OPLS-DA score plots for CK vs. L vs. Y (**A**). OPLS-DA score plots for CK vs. L (**B**). OPLS-DA score plots for CK vs. Y (**C**). OPLS-DA validation chart for CK vs. L vs. Y (**D**). Validation plot of OPLS-DA for CK vs. L (**E**). Validation plot of OPLS-DA for CK vs. Y (**F**).

The PCA of the detected 1233 volatile substances was conducted to acquire the compound composition differences among the CK, L, and Y groups. The cumulative variance contribution of PC1 and PC2 was 67.41% (Figure 6), indicating that the PC1 and PC2 after dimensionality reduction can describe 67.41% of total compound data. In the PCA space, the sample points of three experimental groups show a significant separation tendency, with the Y group and the CK group demonstrating the most pronounced separation on the PC1 axis, which manifests that there are significant differences in the volatile compound composition among the CK, L, and Y groups. Additionally, all groups of samples exhibited satisfactory intra-group clustering in the PCA space, thereby signifying that the experimental reproducibility satisfied the criteria of the analysis. The OPLS-DA results of the overall volatile compounds in the three groups also corroborated the above-mentioned conclusions. The OPLS-DA results of the overall volatile compounds in the three groups also corroborated the above-mentioned conclusions. As shown in Figure 7A–C, all of T scores of OPLS-DA in the three group comparisons (CK vs. L vs. Y, CK vs. L, and CK vs. Y) were larger than the corresponding Orthogonal T scores, indicating that the predicted principal component of each OPLS-DA model can effectively stand for the most of the volatile compound data. The sample points of the L and Y groups form a significant spatial separation from the CK group on the abscissa axis, with the Y group being the most significantly separated from the CK group, suggesting that *S. cerevisiae* had a more prominent role in changing the volatile compound composition of squid cartilage. Furthermore, all of Q^2^ value of the model validation data were larger than 0.9 (Figure 7D–F), demonstrating that the proposed OPLS-DA model showed both excellent predictive efficacy and explanatory ability, and could reliably support the screening of differential metabolites.

#### 3.4.2. Screening of Differential Volatile Compounds

Based on the criteria of VIP value > 1 from OPLS-DA results and *p*-value < 0.05 from univariate analysis of the relative abundance of volatile compounds, a total of 509 significantly differential compounds were screened among the CK, L, and Y groups (Supplementary Table S2). These included 162 types of aldehydes, ketones and esters, 105 hydrocarbons, 67 alcohol and amines compounds, 56 heterocyclic compounds, 38 terpenoids, 25 organic acid and its derivatives, 23 benzene and substituted derivatives, 14 nitrogen compounds, 12 ethers, 5 halogenated hydrocarbons and 2 other compounds.

**Figure 8:**
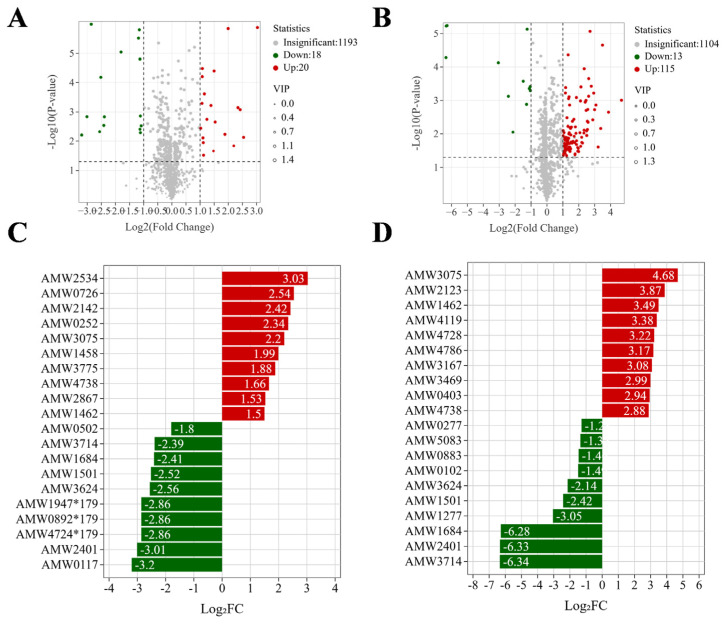
Differential metabolite volcano plots for CK vs. L and CK vs. Y (**A**,**B**). In the volcano diagram, each dot denotes a metabolite, with green dots indicating down-regulated differential metabolites, red dots denoting up-regulated differential metabolites, and gray dots representing metabolites that were detected but did not exhibit a significant difference. Differential ploidy (top 20) bar graphs for CK vs. L and CK vs. Y (**C**,**D**). Red indicates an increase in metabolite levels, whereas green indicates a decrease.

The fold change (FC) analysis of the volatile compounds was performed to screen differential substances between the CK, L, and Y groups based on the criteria of FC ≥ 2 and FC ≤ 0.5. In the two group comparisons (CK vs. L, CK vs. Y), 38, and 128 significantly differential compounds in the relative content were screened (Figure 8A,B), respectively. Thus, the regulation of *S. cerevisiae* in the relative volatile compound content of squid cartilage was greater than *L. plantarum*. Thereinto, the volatile compounds of FC in the top 20 are shown in Figure 8C,D (Supplementary Tables S3 and S4). These were predominantly enriched in aldehyde, Ketones and, Esters heterocyclic compounds, alcohol and amines, hydrocarbons, nitrogen compounds and so on. Specifically, a total of nine aldehydes, ketones, and esters were detected to be significantly up-regulated in the CK vs. Y comparison, while only six such compounds were found to show a significant up-regulation trend in the CK vs. L comparison. This outcome is consistent with the group separation characteristics of the clustered heat map analysis and the electronic nose sensor response pattern.

#### 3.4.3. Screening of Key Flavor Substances

**Figure 9:**
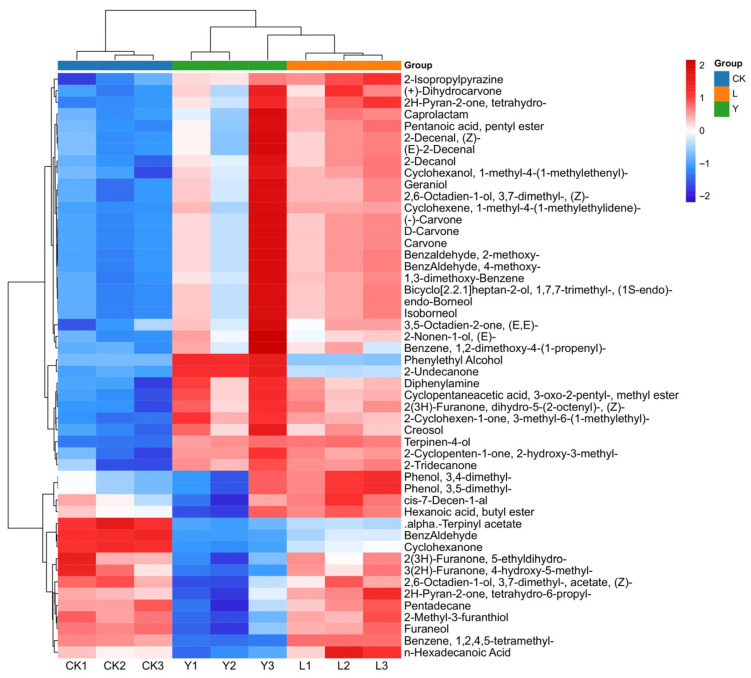
Heat map of 50 key flavor compounds (VIP value > 1.0, rOAV > 1.0, and *p* < 0.059) (red for high content, green for low content).

ROAV is a core indicator that characterizes the degree of contribution of active compounds to the aroma of a sample. Research has demonstrated that this parameter can effectively reflect the intensity of the substance’s contribution to the overall aroma by quantifying the ratio of the compound concentration to the olfactory threshold. Research has demonstrated that when the ROAV of a compound exceeds 1, it can be deduced that the substance under scrutiny has a substantial impact on the distinctive aroma of the sample [23,28]. This class of substance is typically regarded as the pivotal aroma-active ingredient. A total of 50 key flavor substances were identified with the criteria of VIP value > 1.0, ROAV > 1.0 as well as *p* < 0.05 (Table 7). These substances encompassed 7 alcohols, 8 esters, 13 ketones 6 aldehydes and 16 other compounds. Among them, aldehydes and alcohols are mainly involved in the formation of fishy flavors, while esters and ketones mainly form aromatic substances. Heat map analysis indicated that both *S. cerevisiae* and *L. plantarum* fermentation effectively degraded key fishy odor-associated compounds in squid cartilage (Figure 9), thereby reducing their undesirable flavor intensity.

Six aldehyde compounds were identified as key flavor compounds in squid cartilage homogenates. Among them, benzaldehyde—which exhibits a bitter almond odor and relatively high relative content—was confirmed as the primary source of fishy off-flavor. This finding aligns with results from other seafood studies, indicating that this compound originates from lipid oxidation or amino acid degradation [29,30]. Similarly, cis-7-Decenal, an unsaturated aldehyde with fatty and herbal notes, was identified as a potential off-flavor contributor. Fermentation with *S. cerevisiae* and *L. plantarum* reduced benzaldehyde levels by 77% and 55%, respectively, while cis-7-Decenal decreased by 11.5% and 0.1%. Additionally, 2-methyl-3-furanthiol—a sulfur compound responsible for strong fishy and meaty odors—was significantly reduced by 18.5% after *S. cerevisiae* treatment and by 6.6% after *L. plantarum* treatment. The high efficiency of *S. cerevisiae* aligns with its broader activity in degrading aldehydes and sulfides, consistent with findings from yeast fermentation studies on seafood byproducts [31].

**Figure 10:**
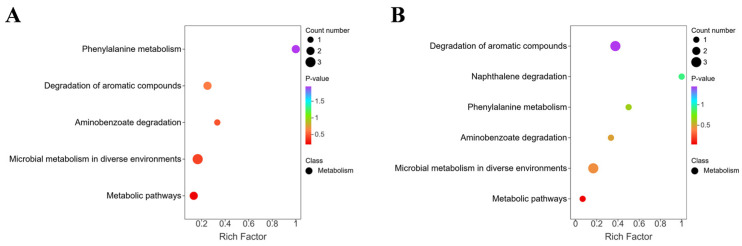
Differential metabolite pathway enrichment plot for CK vs. L (**A**). Differential metabolite pathway enrichment plot for CK vs. Y (**B**).

The mechanism for removing off-flavor compounds can be attributed to specific enzyme activities and metabolic pathways. In *S. cerevisiae*, the degradation of aldehydes such as benzaldehyde may occur via aldehyde reductases and alcohol dehydrogenases, which reduce aldehydes to less volatile alcohols [32]. Furthermore, the enrichment of aromatic compound degradation pathways—including naphthalene degradation, benzoate degradation, and polycyclic environmental microbial metabolism (Figure 10B)—indicates that dioxygenases participate by catalyzing aromatic ring cleavage, thereby neutralizing fishy odor precursors. For sulfur-containing compounds (e.g., 2-methyl-3-furancarboxylic acid), *S. cerevisiae* may metabolize these off-flavor compounds into odorless intermediates via sulfide oxidases or participation in methionine cycle pathways. This mechanism aligns with findings in other food systems, such as cheese fermentation, where yeast has been shown to possess an efficient methionine uptake and metabolic system capable of generating or converting sulfur-containing flavor compounds [33]. Our findings suggest *S. cerevisiae* may activate similar metabolic pathways, but with a focus on degrading rather than producing off-flavor compounds, thereby achieving efficient deodorization.

Although *L. plantarum* exhibits lower efficiency, it contributes to deodorization by converting aldehydes into acids or esters via aldehyde dehydrogenase and esterase. However, its deodorization effect remains limited due to restricted metabolic regulation and relatively fewer metabolic pathways (Figure 10A). This aligns with prior studies indicating that lactic acid bacteria exhibit narrower substrate specificity than *S. cerevisiae* during deodorization [34,35].

Beyond odor reduction, both microorganisms promote the synthesis of pleasant aroma compounds. Ketonones such as piperitone, p-anisalacetone, and (+)-dihydrocarvone significantly increased, imparting minty, sweet, and bready notes—consistent with findings in aquatic microbial fermentation studies [31,36]. Esters such as δ-decalactone, amyl valerate, and (Z)-6-dodecen-γ-lactone also increased significantly, contributing fruity and buttery aromas. This effect can be attributed to enhanced esterase and lipase activity during fermentation [37,38].

It could be seen that the relative content of the key volatile fishy compounds such as benzaldehyde and 2-Methyl-3-furanethiol significantly decreased after fermentation by *S. cerevisiae* and *L. plantarum*, but the key volatile aroma compounds including carvone and geraniol increased obviously. At the same time, *S. cerevisiae* has a better deodorizing and aroma-enhancing effect than *L. plantarum*.

## 4. Conclusions

In conclusion, this study successfully determined the optimized parameters for the deodorizing fermentation of squid cartilage homogenate using *S. cerevisiae* and *L. plantarum*. The optimal fermentation conditions for *S. cerevisiae* were as follows: fermentation time of 105 min, temperature of 34 °C, and inoculum level of 0.85%. For *L. plantarum*, the optimal conditions were as follows: fermentation time of 79 min, temperature of 34.5 °C, and inoculum level of 4.5%. Electronic nose and HS-SPME-GC-MS analyses confirmed that *S. cerevisiae* demonstrated superior performance in eliminating key fishy odor compounds (particularly sulfur-containing compounds and aldehydes) while promoting the formation of desirable aroma compounds such as esters and ketones (e.g., carvone and δ-pentenol). Mechanistic analysis suggests that *S. cerevisiae*’s enhanced deodorization efficiency may stem from its multi-pathway synergistic metabolism, including enzymes such as dioxygenases and sulfide oxidases involved in aromatic compound degradation. These enzymatic reactions facilitate the conversion of malodorous substances into odorless or pleasant aromas.

This study has the following limitations: Research conducted under controlled laboratory conditions may face challenges in maintaining fermentation parameter stability when scaled up to industrial production. Furthermore, the precise enzymatic pathways and genetic regulatory mechanisms underlying the metabolic activities of *S. cerevisiae* and *L. plantarum* remain incompletely elucidated, necessitating proteomics and transcriptomics analyses. Simultaneously, the potential impact of fermentation on the final product’s texture and nutritional value requires in-depth investigation to ensure comprehensive application in food processing. Despite these limitations, this study establishes a valuable theoretical and practical foundation for the high-value utilization of squid processing by-products and offers a highly promising bio-deodorization strategy for aquatic products.

## Figures and Tables

**Table 1:** Factors and level settings for the Box–Behnken test for *Saccharomyces cerevisiae* deodorization.

Factor	Name	Low Actual	High Actual	Low Coded	High Coded
A	fermentation time (min)	60	120	−1	1
B	Fermentation temperature (°C)	30	40	−1	1
C	Saccharomyces addition (%)	0.6	1.0	−1	1

**Table 2:** Factors and level settings for the Box–Behnken test of *Lactobacillus plantarum* deodorization.

Factor	Name	Low Actual	High Actual	Low Coded	High Coded
A	fermentation time (min)	30	90	−1	1
B	fermentation temperature (°C)	30	40	−1	1
C	Saccharomyces addition (%)	2	6	−1	1

**Table 3:** Box–Behnken design test plan and results for *Saccharomyces cerevisiae* deodorization.

Run	Independent Variable			Response	
	A	B	C	Sensory Score	Predicted
1	90	40	0.6	2.5	2.58
2	120	30	0.8	2.5	2.65
3	90	35	0.8	4.2	4.44
4	90	35	0.8	4.5	4.44
5	120	35	1	3	2.93
6	90	40	1	3.2	3.23
7	90	35	0.8	4.5	4.44
8	60	35	1	3.2	3.33
9	60	40	0.8	3.2	3.05
10	60	30	0.8	3.5	3.45
11	90	30	0.6	3.2	3.18
12	120	35	0.6	2.2	2.08
13	120	40	0.8	2.2	2.25
14	90	30	1	3.5	3.43
15	90	35	0.8	4.5	4.44
16	90	35	0.8	4.5	4.44
17	60	35	0.6	3.2	3.28

**Table 4:** Box–Behnken design test plan and results for deodorization of *Lactobacillus plantarum*.

Run	Independent Variable			Response	
	A	B	C	Sensory Score	Predicted
1	60	35	4	4.4	4.2
2	90	35	6	3	3.04
3	60	30	2	2.5	2.65
4	90	35	2	2	1.91
5	60	40	2	2.2	2.19
6	90	40	4	2	2.11
7	60	35	4	4.2	4.2
8	60	30	6	3	3.03
9	60	35	4	4	4.2
10	30	30	4	3.2	3.09
11	30	35	2	3.2	3.16
12	30	35	6	2.5	2.59
13	60	35	4	4.2	4.2
14	90	30	4	2.5	2.44
15	60	35	4	4.2	4.2
16	60	40	6	2.5	2.35
17	30	40	4	2.2	2.26

**Table 5:** Analysis of variance of the *Saccharomyces cerevisiae* deodorization return model.

Source	Sum of Square	Degrees of Freedom	Mean Square	F Value	*p* Value	Significance
Model	10.18	9	1.13	44.74	<0.0001	**
A	1.28	1	1.28	50.62	0.0002	**
B	0.3200	1	0.3200	12.66	0.0093	**
C	0.4050	1	0.4050	16.02	0.0052	*
AB	0.0000	1	0.0000	0.0000	1.0000	
AC	0.1600	1	0.1600	6.33	0.0401	*
BC	0.0400	1	0.0400	1.58	0.2488	
A^2^	3.37	1	3.37	133.99	<0.0001	**
B^2^	2.03	1	2.03	80.43	<0.0001	**
C^2^	1.75	1	1.75	69.28	<0.0001	**
Residual	0.1770	7	0.0253			
Lack of Fit	0.1050	3	0.0350	1.94	0.2643	Not significant
Pure Error	0.0720	4	0.0180			
Cor Total	10.36	16				
R^2^	0.9829					
R^2^_Adj_	0.9609					

Note: * *p* < 0.05, ** *p* < 0.001.

**Table 6:** Analysis of variance of the *Lactobacillus plantarum* deodorization model.

Source	Sum of Square	Degrees of Freedom	Mean square	F Value	*p* Value	Significance
Model	11.42	9	1.27	50.06	<0.0001	**
A	0.3200	1	0.3200	12.62	0.0193	*
B	0.6612	1	0.6612	26.08	0.0001	**
C	0.1512	1	0.1512	5.96	0.0838	
AB	0.0625	1	0.0625	2.46	0.1182	
AC	0.7225	1	0.7225	28.49	0.0005	**
BC	0.0100	1	0.0100	0.3944	0.1182	
A^2^	2.69	1	2.69	106.27	<0.0001	**
B^2^	3.60	1	3.60	142.08	<0.0001	**
C^2^	2.21	1	2.21	87.28	<0.0001	**
Residual	0.1775	7	0.0254			
Lack of Fit	0.0975	3	0.0325	1.62	0.3177	Not significant
Pure Error	0.0800	4	0.0200			
Cor Total	11.60	16				
R^2^	0.9847					
R^2^_Adj_	0.9650					

Note: * *p* < 0.05, ** *p* < 0.001.

**Table 7:** Changes in volatile flavor substances during fermentation of squid cartilage homogenates.

Number	RI	Volatile Aromatic Compound	Classes	Relative Contents (μg/g)			Aroma Description
				CK	L	Y	
1	1246	D-Carvone	Ketones	2.779 ± 0.02 ^b^	3.038 ± 0.03 ^a^	3.054 ± 0.19 ^a^	minty, bread,
2	1242	Carvone	Ketones	2.779 ± 0.02 ^b^	3.038 ± 0.03 ^a^	3.054 ± 0.19 ^a^	minty, licorice
3	1249.95	(−)-Carvone	Ketones	2.78 ± 0.02 ^b^	3.04 ± 0.03 ^a^	3.05 ± 0.19 ^a^	spearmint, minty
4	1294.65	2-Undecanone	Ketones	0.009 ± 0.00 ^c^	0.012 ± 0.00 ^b^	0.022 ± 0.00 ^a^	fruity, creamy, floral
5	1261.17	Piperitones	Ketones	0.011 ± 0.00 ^b^	0.015 ± 0.00 ^a^	0.017 ± 0.00 ^a^	herbal, minty
6	1070	Furaneol	Ketones	0.101 ± 0.00 ^a^	0.100 ± 0.00 ^a^	0.079 ± 0.01 ^b^	caramel
7	1054	3(2H)-Furanone, 4-hydroxy-5-methyl-	Ketones	0.015 ± 0.00 ^a^	0.014 ± 0.00 ^a^	0.011 ± 0.00 ^b^	Caramel
8	1034	2-Cyclopenten-1-one, 2-hydroxy-3-methyl-	Ketones	0.044 ± 0.00 ^b^	0.069 ± 0.00 ^a^	0.061 ± 0.00 ^a^	caramel,
9	1287	2H-Pyran-2-one, tetrahydro-6-propyl-	Ketones	0.020 ± 0.00 ^a^	0.021 ± 0.00 ^a^	0.017 ± 0.00 ^b^	coconut
10	1210	(+)-Dihydrocarvone	Ketones	0.019 ± 0.00 ^b^	0.021 ± 0.00 ^a^	0.021 ± 0.00 ^a^	minty
11	1495.82	2-Tridecanone	Ketones	0.111 ± 0.02 ^b^	0.157 ± 0.00 ^a^	0.158 ± 0.01 ^a^	fatty, earthy
12	1073	3,5-Octadien-2-one, (E,E)-	Ketones	1.993 ± 0.07 ^b^	2.153 ± 0.04 ^ab^	2.158 ± 0.13 ^a^	fruity, grassy
13	1102	L-Fenchone	Ketones	0.244 ± 0.01 ^b^	0.259 ± 0.00 ^ab^	0.267 ± 0.02 ^a^	camphor,
14	1116.09	Phenylethyl Alcohol	Alcohols	0.004 ± 0.00 ^b^	0.025 ± 0.00 ^b^	0.356 ± 0.02 ^a^	fruity, rose,
15	1255.61	Geraniol	Alcohols	0.196 ± 0.00 ^b^	0.214 ± 0.00 ^a^	0.216 ± 0.01 ^a^	floral, fruity
16	1230.47	2,6-Octadien-1-ol, 3,7-dimethyl-, (Z)-	Alcohols	0.196 ± 0.00 ^b^	0.214 ± 0.00 ^a^	0.216 ± 0.01 ^a^	lemon
17	1181.44	Terpinen-4-ol	Alcohols	0.017 ± 0.00 ^b^	0.039 ± 0.00 ^a^	0.037 ± 0.00 ^a^	nutmeg
18	1198	2-Decanol	Alcohol	0.035 ± 0.00 ^b^	0.039 ± 0.00 ^a^	0.039 ± 0.00 ^a^	coconut
19	1168.13	2-Nonen-1-ol, (E)-	Alcohol	0.416 ± 0.01 ^b^	0.445 ± 0.00 ^ab^	0.464 ± 0.03 ^a^	melon
20	1153	Cyclohexanol, 1-methyl-4-(1-methylethenyl)-	Alcohol	1.489 ± 0.05 ^b^	1.651 ± 0.02 ^a^	1.656 ± 0.10 ^a^	woody
21	1350	alpha. -Terpinyl acetate	Esters	0.032 ± 0.00 ^a^	0.016 ± 0.00 ^b^	0.010 ± 0.00 ^c^	lavender, citrus
22	1365.22	2,6-Octadien-1-ol, 3,7-dimethyl-, acetate, (Z)-	Esters	0.017 ± 0.00 ^a^	0.017 ± 0.00 ^a^	0.013 ± 0.00 ^b^	floral, pear
23	1665.06	Cyclopentaneacetic acid, 3-oxo-2-pentyl-, methyl ester	Esters	1.266 ± 0.05 ^b^	1.530 ± 0.02 ^a^	1.588 ± 0.08 ^a^	floral, oily, jasmin, green
24	1662	(Z)-6-dodecen-γ-lactone	Esters	1.746 ± 0.07 ^b^	2.132 ± 0.05 ^a^	2.183 ± 0.11 ^a^	creamy, fruity
25	1150	Pentyl valerate	Esters	3.306 ± 0.04 ^b^	3.593 ± 0.04 ^a^	3.574 ± 0.23 ^ab^	fruity
26	1054.86	2(3H)-Furanone, 5-ethyldihydro-	Esters	0.303 ± 0.02 ^a^	0.292 ± 0.01 ^a^	0.239 ± 0.02 ^b^	sweet, caramel
27	961	δ-pentolactone	Esters	0.133 ± 0.00 ^b^	0.153 ± 0.00 ^a^	0.149 ± 0.01 ^a^	fruity, sweet
28	1189.39	Hexanoic acid, butyl ester	Esters	0.495 ± 0.01 ^a^	0.529 ± 0.01 ^a^	0.447 ± 0.08 ^a^	fruity,
29	1239	Benzaldehyde, 2-methoxy-	Aldehydes	5.050 ± 0.05 ^b^	5.514 ± 0.06 ^a^	5.545 ± 0.35 ^a^	wax
30	1259.75	BenzAldehyde, 4-methoxy-	Aldehydes	5.050 ± 0.05 ^b^	5.514 ± 0.06 ^a^	5.545 ± 0.35 ^a^	floral, balsamic
31	962.46	BenzAldehyde	Aldehydes	0.427 ± 0.02 ^b^	0.205 ± 0.03 ^a^	0.101 ± 0.19 ^a^	bitter, almond
32	1252	2-Decenal, (Z)-	Aldehydes	0.157 ± 0.00 ^a^	0.169 ± 0.00 ^a^	0.169 ± 0.01 ^a^	tallow
33	1263	(E)-2-Decenal	Aldehydes	0.157 ± 0.00 ^a^	0.169 ± 0.00 ^a^	0.169 ± 0.01 ^a^	fatty
34	1199	Cis-7-decenal	Aldehydes	0.371 ± 0.01 ^ab^	0.394 ± 0.01 ^a^	0.345 ± 0.03 ^b^	Fatty, grassy
35	870	2-Methyl-3-furanthiol	Others	0.071 ± 0.00 ^a^	0.070 ± 0.00 ^a^	0.060 ± 0.00 ^b^	fishy, metallic
36	1170.42	endo-Borneol	Others	1.586 ± 0.02 ^b^	1.731 ± 0.02 ^a^	1.743 ± 0.11 ^a^	woody, balsamic
37	1157	Isoborneol	Others	1.586 ± 0.02 ^b^	1.731 ± 0.02 ^a^	1.743 ± 0.11 ^a^	balsamic
38	1170	Bicyclo[2.2.1] heptan-2-ol, 1,7,7-trimethyl-, (1S-endo)-	Others	1.586 ± 0.02 ^b^	1.731 ± 0.02 ^a^	1.743 ± 0.11 ^a^	pine, woody, camphor
39	1090.66	Cyclohexene, 1-methyl-4-(1-methylethylidene)-	Others	0.053 ± 0.00 ^b^	0.058 ± 0.00 ^a^	0.059 ± 0.00 ^a^	citrus, pine
40	1499.37	Pentadecane	Others	0.124 ± 0.00 ^b^	0.123 ± 0.00 ^a^	0.093 ± 0.01 ^a^	waxy
41	1634.64	Diphenylamine	Others	0.135 ± 0.01 ^b^	0.159 ± 0.00 ^a^	0.165 ± 0.01 ^a^	sweet, floral,
42	1168	1,3-dimethoxy-Benzene	Others	0.169 ± 0.00 ^b^	0.183 ± 0.00 ^a^	0.184 ± 0.01 ^a^	fruity, neroli
43	1115	Benzene, 1,2,4,5-tetramethyl-	Others	0.010 ± 0.00 ^b^	0.011 ± 0.00 ^a^	0.002 ± 0.00 ^c^	sweet
44	1194.62	Creosol	Others	0.836 ± 0.01 ^b^	0.921 ± 0.01 ^a^	0.948 ± 0.04 ^a^	smoky
45	1253.70	Caprolactam	Others	1.032 ± 0.01 ^a^	1.116 ± 0.01 ^a^	1.107 ± 0.07 ^a^	spicy
46	1193	Phenol, 3,4-dimethyl-	Others	0.070 ± 0.00 ^a^	0.074 ± 0.00 ^a^	0.069 ± 0.00 ^a^	flat, dry
47	1171	Phenol, 3,5-dimethyl-	Others	0.070 ± 0.00 ^a^	0.074 ± 0.00 ^a^	0.069 ± 0.00 ^a^	coffee
48	988	2-Isopropylpyrazine	Others	0.025 ± 0.00 ^b^	0.035 ± 0.00 ^a^	0.033 ± 0.00 ^a^	minty, honey
49	1492	Benzene, 1,2-dimethoxy-4-(1-propenyl)-	Others	9.721 ± 0.10 ^b^	10.333 ± 0.21 ^ab^	10.712 ± 0.70 ^a^	spicy clove
50	1968	n-Hexadecanoic Acid	Others	0.011 ± 0.00 ^a^	0.020 ± 0.00 ^a^	0.011 ± 0.00 ^b^	fatty

Note: RI: retention index; values are expressed as mean ± standard deviation (*n* = 3). Values in the same row with different superscript letters (a, b, c) are significantly different (*p* < 0.05) by Duncan’s multiple range test.

## Data Availability

The original contributions presented in this study are included in the article/Supplementary Materials. Further inquiries can be directed to the corresponding authors.

## Supplementary Materials

The following supporting information can be downloaded at: https://www.mdpi.com/article/10.3390/foods14173117/s1. Table S1 1232 poorly volatile compounds detected in CK, L and Y groups. Table S2 509 significantly different compounds screened in CK, L and Y groups. Table S3 Annotated list of CK vs. L differential metabolites. Table S4 Annotated list of CK vs. Y differential metabolites.

## Abbreviations

The following abbreviations are used in this manuscript:

VOCs	Volatile Organic Compounds
HS-SPME-GC-MS	headspace solid-phase microextraction gas chromatography-mass spectrometry
ROAV	Relative odor activity value
PCA	Principal component analysis
OPLS-DA	Orthogonal partial least squares discriminant analysis
VIP	Variable importance projection
FC	Fold Change
