# Supplementary material for: Comparison of Odor Mitigation in Squid Cartilage Fermented by Saccharomyces cerevisiae and Lactobacillus plantarum
Source: Foods. 2025 Sep 6;14(17):3117. doi: 10.3390/foods14173117 (PMC12428700; doi:10.3390/foods14173117)
Supplement: Supplementary file 1 [file foods-14-03117-s001.zip › foods-3811091-supplementary.pdf]

Supplementary Data

Comparison of Odor Mitigation in Squid Cartilage Fermented by *Saccharomyces cerevisiae* and *Lactobacillus plantarum*

Tingting Zhang [1,†], Rongbin Zhong [1,†], Feifei Shi [1], Qian Yang [1], Peng Liang [1,2,*] and Jiacong Deng [3,*]

[1] College of Food Science, Fujian Agriculture and Forestry University, Fuzhou 350002, China

[2] College of Oceanology and Food Science, Quanzhou Normal University, Quanzhou 362000, China

[3] College of Food and Biological Engineering, Fujian Polytechnic Normal University, Fuzhou 350300, China

* Correspondence: liangpeng137@sina.com (P.L.); dengjc810508@163.com (J.D.); Tel.: +86-15980598934 (P.L.); +86-18259032940 (J.D.)

† These authors contributed equally to this work.

Captions:

Table S1 1232 poorly volatile compounds detected in CK, L and Y groups.

Table S2 509 significantly different compounds screened in CK, L and Y groups.

Table S3 Annotated list of CK vs. L differential metabolites.

Table S4 Annotated list of CK vs. Y differential metabolites.

Table S1 1232 poorly volatile compounds detected in CK, L and Y groups

Number	Index	Compounds	Class I	Formula	Odor
1	IS-04	3-Hexanone-2,2,4,4-d4	Aldehyde, Ketones, Esters	C6H8D4O	-
2	AMW0940*166	D-Carvone	Terpenoids	C10H14O	spice, minty, bread, caraway
3	AMW2871*365	Decane, 2,3,6-trimethyl-	Hydrocarbons	C13H28	-
4	AMW0813*092	2-Octen-1-ol, (E)-	Alcohol and amines	C8H16O	green, citrus, vegetable, fatty
5	AMW1445*087	o-Cymene	Benzene and substituted derivatives	C10H14	gasoline
6	AMW4723*232	3-Hexenoic acid, (E)-	Organic acid and Its derivatives	C6H10O2	Sweaty, body odour
7	AMW4914*387	Benzaldehyde, 2-methoxy-	Aldehyde, Ketones, Esters	C8H8O2	wax, medicinal
8	AMW0542*287	Isobornyl acetate	Aldehyde, Ketones, Esters	C12H20O2	balsamic, camphor, herbal, woody, sweet
9	AMW4851*339	cis-Ocimenol	Terpenoids	C10H18O	-
10	AMW1219*069	3,5-Octadien-2-one	Aldehyde, Ketones, Esters	C8H12O	fruity, fatty, mushroom
11	AMW1589*112	Ethanone, 1-(2-methylphenyl)-	Aldehyde, Ketones, Esters	C9H10O	sweet, hawthorn, powdery, anisic, coumarin, phenol, burnt, nutty, honey
12	AMW2371*353	Tridecane, 2-methyl-	Hydrocarbons	C14H30	-
13	AMW1220*096	(E)-3-Nonen-2-ol	Alcohol and amines	C9H18O	-
14	AMW0556*088	3-methyl-Decane	Hydrocarbons	C11H24	-
15	AMW2004*342	(Z)-8-methyl-2-Decene	Hydrocarbons	C11H22	-
16	AMW3258*367	Nonane, 2-methyl-5-propyl-	Hydrocarbons	C13H28	-
17	AMW0208*113	L-.alpha.-Terpineol	Terpenoids	C10H18O	lilac, floral, terpenic
18	AMW4684*211	2(3H)-Furanone, dihydro-4-methyl-	Aldehyde, Ketones, Esters	C5H8O2	-
19	AMW2006*342	(Z)-4-methyl-2-Decene	Hydrocarbons	C11H22	-
20	AMW1115*345	5-methyl-5-Undecene	Hydrocarbons	C12H24	-
21	AMW0233*125	Geraniol	Terpenoids	C10H18O	sweet, floral, fruity, rose, waxy, citrus
22	AMW0999*269	2-Decenal, (Z)-	Aldehyde, Ketones, Esters	C10H18O	tallow
23	AMW2600*363	(3S,3aS,8aR)-6,8a-Dimethyl-3-(prop-1-en-2-yl)-	Terpenoids	C15H24	-

		1,2,3,3a,4,5,8,8a-octahydroazulene			
24	AMW2654*263	(3E,5Z)-1,3,5-Undecatriene	Hydrocarbons	C11H18	-
25	AMW2231*393	1-ethyl-2,3-dimethyl-Benzene	Hydrocarbons	C10H14	-
26	AMW2356*338	endo-Borneol	Terpenoids	C10H18O	pine, woody, camphor, balsamic
27	AMW2309*344	Propanoic acid, 2-methyl-, 3-methylbutyl ester	Aldehyde, Ketones, Esters	C9H18O2	fruity, ethereal, tropical, green, grape, cherry, unripe banana, apple, cocoa
28	AMW4849*270	Cyclohexanol, 1-methyl-4-(1-methylethenyl)-	Terpenoids	C10H18O	pungent, earthy, woody
29	AMW0703*347	Undecane, 5-methyl-	Hydrocarbons	C12H26	-
30	AMW1743*107	2,6,8-trimethyl-Decane	Hydrocarbons	C13H28	-
31	AMW2559*236	Phenol, 3,4-dimethyl-	Benzene and substituted derivatives	C8H10O	flat, dry
32		1H-3a,7-Methanoazulene, 2,3,4,7,8,8a-hexahydro-3,6,8,8-			
	AMW2512*359	tetramethyl-, [3R-(3.alpha.,3a.beta.,7.beta.,8a.alpha.)]-	Terpenoids	C15H24	woody, cedary, sweet, fresh
33	AMW1739*068	2,2,6-trimethyl-Octane	Hydrocarbons	C11H24	-
34	AMW1535*254	2,6-Nonadienal, (E,Z)-	Aldehyde, Ketones, Esters	C9H14O	cucumber, green
35	AMW1494*273	1-(2-butoxyethoxy)-Ethanol	Alcohol and amines	C8H18O3	-
36	AMW2242*221	o-Toluidine	Alcohol and amines	C7H9N	-
37	AMW2814*366	Dodecane, 6-methyl-	Hydrocarbons	C13H28	-
38	AMW2718*311	Benzene, 1-ethyl-4-(1-methylethyl)-	Benzene and substituted derivatives	C11H16	-
39	AMW1740*372	2,3,6-trimethyl-Octane	Hydrocarbons	C11H24	-
40	AMW0571*326	3-Carene	Terpenoids	C10H16	citrus, herbal, pine, solvent, resinous, phenol, cypress, medicinal, woody
41	AMW0278*099	1-methyl-2-propyl-Benzene	Benzene and substituted derivatives	C10H14	-
42	AMW2232*105	Benzene, 1-ethyl-3,5-dimethyl-	Benzene and substituted derivatives	C10H14	-
43	AMW2270*051	2-Furanmethanol	Alcohol and amines	C5H6O2	alcoholic, chemical, musty, sweet, caramel, bread, coffee
44	AMW0572*100	1,2-diethyl-Benzene	Benzene and substituted derivatives	C10H14	-
45	AMW4595*214	3-Hexen-1-ol, (Z)-	Alcohol and amines	C6H12O	Green
46	AMW1190*248	1-(6-methyl-3-pyridinyl)-Ethanone	Heterocyclic compounds	C8H9NO	-
47	AMW1528*329	.beta.-Phellandrene	Terpenoids	C10H16	terpenic, herbal

48	AMW0931*319	3-methyl-Octane	Hydrocarbons	C9H20	-
49	AMW0001*117	2,8,8-trimethyl-Decane	Hydrocarbons	C13H28	-
50	AMW1629*335	3-methyl-Nonane	Hydrocarbons	C10H22	-
51	AMW1703*122	4-methyl-4-Undecene	Hydrocarbons	C12H24	-
52	AMW0395*377	4-methyl-Pentanamide	Alcohol and amines	C6H13NO	-
53	AMW2736*330	Cyclooctane, 1,4-dimethyl-, trans-	Terpenoids	C10H20	-
54	AMW4452*118	2-Nonen-1-ol, (E)-	Alcohol and amines	C9H18O	waxy, green, violet, melon
55	AMW0828*226	(E)-2-Heptenal	Aldehyde, Ketones, Esters	C7H12O	pungent, green, vegetable, fresh, fatty
56	AMW1979*381	4-Hexen-1-ol, acetate	Aldehyde, Ketones, Esters	C8H14O2	-
57	AMW4496*332	3,5-Dimethyl-4-heptanone	Aldehyde, Ketones, Esters	C9H18O	-
58	AMW4427*237	2-Cyclopenten-1-one, 2,3,4-trimethyl-	Aldehyde, Ketones, Esters	C8H12O	-
59	AMW3031*248	5-Acetyl-2-methylpyridine	Heterocyclic compounds	C8H9NO	-
60	AMW1091*346	Undecane, 4-methyl-	Hydrocarbons	C12H26	-
61	AMW2349*351	Pentanoic acid, 2-methylbutyl ester	Aldehyde, Ketones, Esters	C10H20O2	-
62	AMW0771*368	Decane, 3-ethyl-3-methyl-	Hydrocarbons	C13H28	-
63	AMW2878*316	(+)-epi-Bicyclosesquiphellandrene	Terpenoids	C15H24	-
64	AMW0932*318	4-methyl-Octane	Hydrocarbons	C9H20	-
65	AMW0862*111	3-Cyclohexen-1-ol, 4-methyl-1-(1-methylethyl)-, (R)-	Terpenoids	C10H18O	-
66	AMW0777*369	Undecane, 4,6-dimethyl-	Hydrocarbons	C13H28	-
67	AMW1722*045	3-methyl-1H-Pyrrole	Heterocyclic compounds	C5H7N	-
68	AMW0772*369	Undecane, 4,4-dimethyl-	Hydrocarbons	C13H28	-
69	AMW3081*352	n-Amyl isovalerate	Aldehyde, Ketones, Esters	C10H20O2	apple, fresh fruit
70	AMW1736*061	2,5,6-trimethyl-Octane	Hydrocarbons	C11H24	-
71	AMW3102*253	3-Cyclohexen-1-one, 3,5,5-trimethyl-	Aldehyde, Ketones, Esters	C9H14O	-
72	AMW0630*324	(R)-1-methyl-5-(1-methylethenyl)-Cyclohexene	Terpenoids	C10H16	-
73	AMW1153*327	Bicyclo[3.1.0]hexane, 4-methylene-1-(1-methylethyl)-	Terpenoids	C10H16	woody, terpene, citrus, pine, spice

74	AMW4936*233	Maleic acid	Organic acid and Its derivatives	C4H4O4	-
75	AMW0937*102	2,4-Dimethylstyrene	Benzene and substituted derivatives	C10H12	-
76	AMW0889*288	(1.alpha.,2.beta.,5.beta.)-5-methyl-2-(1-methylethyl)- Cyclohexanol, acetate	Alcohol and amines	C12H22O2	-
77	AMW2965*207	1H-1,2,4-Triazol-3-amine, 5-methyl-	Alcohol and amines	C3H6N4	-
78	AMW0588*079	2-ethyl-6-methyl-Pyrazine	Heterocyclic compounds	C7H10N2	roasted, potato
79	AMW3412*184	Fomepizole	Heterocyclic compounds	C4H6N2	-
80	AMW0776*119	Undecane, 3,5-dimethyl-	Hydrocarbons	C13H28	-
81	AMW0770*126	2,2-dimethyl-Undecane	Hydrocarbons	C13H28	-
82	AMW2540*083	Phenol, 2-methyl-	Benzene and substituted derivatives	C7H8O	phenol
83	AMW0773*126	5,5-dimethyl-Undecane	Hydrocarbons	C13H28	-
84	AMW2667*278	Indan, 1,1,6,7-tetramethyl-	Benzene and substituted derivatives	C13H18	-
85	AMW1050*073	2,6-Octadiene, 2,6-dimethyl-	Hydrocarbons	C10H18	-
86	AMW1747*348	2,2,4,4-Tetramethyloctane	Hydrocarbons	C12H26	-
87	AMW0761*074	Nonane, 4,5-dimethyl-	Hydrocarbons	C11H24	-
88	AMW2175*085	1-Decene	Hydrocarbons	C10H20	-
89	AMW0756*119	Undecane, 2,9-dimethyl-	Hydrocarbons	C13H28	-
90	AMW0626*224	1-ethyl-Cyclohexene	Hydrocarbons	C8H14	-
91	AMW1790*093	1-ethenyl-3-methoxy-Benzene	Benzene and substituted derivatives	C9H10O	-
92	AMW0177*317	.alpha.-Muurolene	Terpenoids	C15H24	woody
93	AMW2488*252	2-Methoxy-5-methylphenol	Benzene and substituted derivatives	C8H10O2	-
94	AMW4939*283	5-Tridecene, (E)-	Hydrocarbons	C13H26	-
95	AMW0754*119	Undecane, 2,6-dimethyl-	Hydrocarbons	C13H28	-
96	AMW1242*255	2,7-dimethyl-1,6-Octadiene	Hydrocarbons	C10H18	-
97	AMW4834*270	3-Cyclohexen-1-ol, 1-methyl-4-(1-methylethyl)-	Terpenoids	C10H18O	dry, woody, musty
98	AMW0755*308	2,8-dimethyl-Undecane	Hydrocarbons	C13H28	-

99	AMW1236*269	(E)-2-Decenal	Aldehyde, Ketones, Esters	$C_{10}H_{18}O$	waxy, fatty, earthy, green, cilantro, mushroom, aldehydic, fried, chicken, fatty, tallow
100	AMW0660*272	2,7-dimethyl-1-Octanol	Alcohol and amines	$C_{10}H_{22}O$	-
101	AMW2243*084	Benzene, 1,2,4-trimethyl-	Benzene and substituted derivatives	C_9H_{12}	plastic
102	AMW0647*142	β-Ionone	Terpenoids	$C_{13}H_{20}O$	floral, woody, sweet, fruity, berry, tropical, beeswax
103	AMW3055*386	1-Heptadecene	Hydrocarbons	$C_{17}H_{34}$	-
104	AMW0283*132	3,5-Dimethyldodecane	Hydrocarbons	$C_{14}H_{30}$	-
105	AMW0762*074	Nonane, 2,5-dimethyl-	Hydrocarbons	$C_{11}H_{24}$	-
106	AMW0768*109	3,7-dimethyl-Decane	Hydrocarbons	$C_{12}H_{26}$	-
107	AMW0243*083	p-Cresol	Benzene and substituted derivatives	C_7H_8O	phenol, narcissus, animalic, mimosa
108	AMW0506*104	Phenol, 4-ethyl-	Benzene and substituted derivatives	$C_8H_{10}O$	phenol, leathery
109	AMW0987*310	2-Undecenal	Aldehyde, Ketones, Esters	$C_{11}H_{20}O$	fresh, fruity, orange, peel
110	AMW2689*357	(1S,5S)-2-Methyl-5-((R)-6-methylhept-5-en-2-yl)bicyclo[3.1.0]hex-2-ene	Terpenoids	$C_{15}H_{24}$	-
111	AMW3482*282	trans-1,10-Dimethyl-trans-9-decalol	Alcohol and amines	$C_{12}H_{22}O$	-
112	AMW2805*312	(1S,4S,4aS)-1-Isopropyl-4,7-dimethyl-1,2,3,4,4a,5-hexahydronaphthalene	Terpenoids	$C_{15}H_{24}$	-
113	AMW1025*143	1,3,6,10-Dodecatetraene, 3,7,11-trimethyl-, (Z,E)-	Terpenoids	$C_{15}H_{24}$	-
114	AMW1456*310	2-Undecenal, E-	Aldehyde, Ketones, Esters	$C_{11}H_{20}O$	fresh, fruity, citrus, orange, peel
115	AMW0789*105	Benzene, 2-ethyl-1,4-dimethyl-	Benzene and substituted derivatives	$C_{10}H_{14}$	-
116	AMW1848*093	Benzene, 1-ethenyl-4-methoxy-	Benzene and substituted derivatives	$C_9H_{10}O$	sweet
117	AMW1175*098	Benzene, 1-ethenyl-4-ethyl-	Benzene and substituted derivatives	$C_{10}H_{12}$	-
118	AMW1046*103	Benzene, 4-ethenyl-1,2-dimethyl-	Benzene and substituted derivatives	$C_{10}H_{12}$	-
119	AMW0685*022	(Z)-2-methyl-3-Hexene	Hydrocarbons	C_7H_{14}	-
120	AMW2201*257	Naphthalene, 2-methyl-	Benzene and substituted derivatives	$C_{11}H_{10}$	sweet, floral, woody
121	AMW1665*266	Cyclohexanone, 2-methyl-5-(1-methylethenyl)-, trans-	Terpenoids	$C_{10}H_{16}O$	warm, herbal

122	AMW5010*285	2-Buten-1-one, 1-(2,6,6-trimethyl-1,3-cyclohexadien-1-yl)-	Terpenoids	C13H18O	natural, sweet, fruity, rose, plum, grape, raspberry, sugar
123	AMW2852*308	Decane, 6-ethyl-2-methyl-	Hydrocarbons	C13H28	-
124	AMW2262*274	Phenol, 2-methoxy-4-(1-propenyl)-	Benzene and substituted derivatives	C10H12O2	sweet, spicy, clove, woody, allspice, carnation, floral
125	AMW2378*293	Tridecane, 4,8-dimethyl-	Hydrocarbons	C15H32	-
126	AMW3359*352	Butanoic acid, 3-methyl-, 3-methylbutyl ester	Aldehyde, Ketones, Esters	C10H20O2	sweet, fruity, green, ripe apple, jammy, tropical
127	AMW4756*099	Benzene, 1-methyl-3-propyl-	Benzene and substituted derivatives	C10H14	-
128	AMW3078*378	1-Heptene, 2,6-dimethyl-	Hydrocarbons	C9H18	-
129	AMW2281*382	Carveol	Terpenoids	C10H16O	minty, spearmint, cool, green, herbal, caraway, spicy
130	AMW1252*374	3-(2-methylpropyl)-Cyclohexene	Hydrocarbons	C10H18	-
131	AMW4965*350	4-Methylnonanoic acid	Organic acid and Its derivatives	C10H20O2	tallowy, roasted, sheep
132	AMW1467*168	(Z)-9-Tetradecenal	Aldehyde, Ketones, Esters	C14H26O	-
133	AMW5019*282	4a(2H)-Naphthalenol, octahydro-4,8a-dimethyl-,(4.alpha.,4a.alpha.,8a.beta.)-	Alcohol and amines	C12H22O	Earthy
134	AMW0971*151	Z-11-Hexadecenoic acid	Organic acid and Its derivatives	C16H30O2	-
135	AMW0636*206	5-Amino-3-methylisoxazole	Alcohol and amines	C4H6N2O	-
136	AMW0568*261	1-(2-methoxypropoxy)-2-Propanol	Alcohol and amines	C7H16O3	-
137	AMW0215*095	1,4-diethenyl-Benzene	Benzene and substituted derivatives	C10H10	-
138	AMW2893*368	Undecane, 3-ethyl-	Hydrocarbons	C13H28	-
139	AMW2202*258	Quinoline, 6-methyl-	Heterocyclic compounds	C10H9N	leathery, tonka, castoreum, tobacco, civet, fecal
140	AMW2627*259	3-Vinyl-1,2-dithiacyclohex-4-ene	Heterocyclic compounds	C6H8S2	-
141	AMW2304*289	Ethyl trans-4-decenoate	Aldehyde, Ketones, Esters	C12H22O2	green, fruity, waxy, cognac
142	AMW0442*144	Phenol, 3,5-bis(1,1-dimethylethyl)-	Benzene and substituted derivatives	C14H22O	-
143	AMW0119*243	1-Hexanol, 2-ethyl-	Alcohol and amines	C8H18O	citrus, fresh, floral, oily, sweet
144	AMW1002*222	Ethanone, 1-(1H-pyrazol-4-yl)-	Aldehyde, Ketones, Esters	C5H6N2O	-
145	AMW1103*277	Naphthalene, 1,2-dihydro-1,1,6-trimethyl-	Benzene and substituted derivatives	C13H16	licorice
146	AMW0565*101	Pyrazine, 3-ethyl-2,5-dimethyl-	Heterocyclic compounds	C8H12N2	potato, cocoa, roasted, nutty

147		1H-Cyclopenta[1,3]cyclopropa[1,2]benzene, octahydro-7-			
		methyl-3-methylene-4-(1-methylethyl)-, [3aS-			
	AMW0581*161	(3a.alpha.,3b.beta.,4.beta.,7.alpha.,7aS*)]-	Terpenoids	C15H24	citrus, fruity, radish
148	AMW1475*061	Octane, 2,6,6-trimethyl-	Hydrocarbons	C11H24	-
149	AMW1387*143	.alpha.-Farnesene	Terpenoids	C15H24	citrus, herbal, lavender, bergamot, myrrh, neroli, green
150		Naphthalene, 1,2,3,4-tetrahydro-1,6-dimethyl-4-(1-			
	AMW1356*290	methylethyl)-, (1S-cis)-	Terpenoids	C15H22	herbal, spice
151	AMW2724*337	3-Cyclohexen-1-one, 2-isopropyl-5-methyl-	Terpenoids	C10H16O	-
152	AMW2585	L-Fenchone	Terpenoids	C10H16O	camphor, herbal, earthy, woody
153	AMW4091	6-Ethyl-5,6-dihydro-2H-pyran-2-one	Aldehyde, Ketones, Esters	C7H10O2	-
154	AMW2720	Tridecane, 3-methylene-	Hydrocarbons	C14H28	-
155	AMW2981	3-Hydroxybutyric acid, t-butyl ester	Aldehyde, Ketones, Esters	C8H16O3	-
156	AMW0396	2-Decanol	Alcohol and amines	C10H22O	anisic, coconut
157	AMW2749	(2S,6R)-2,6-Dibutyl-4-methylpiperidine	Heterocyclic compounds	C14H29N	-
158	AMW4772	Hexanoic acid, 1,1-dimethylethyl ester	Aldehyde, Ketones, Esters	C10H20O2	-
159	AMW0044	5H-Tetrazol-5-Amine	Alcohol and amines	CH3N5	-
160	AMW1126	7-methyl-5-Octen-4-one	Aldehyde, Ketones, Esters	C9H16O	-
161					fruity, banana, pineapple, green, cherry, tropical fruit, ripe fruit, juicy
	AMW0328	Butanoic acid, butyl ester	Aldehyde, Ketones, Esters	C8H16O2	fruity
162	AMW4892	Butanoic acid, 2-hexenyl ester, (E)-	Aldehyde, Ketones, Esters	C10H18O2	Floral, fruity, cheesy, banana peel
163	AMW0785	1,4-Dihydro-4-oxopyridazine	Heterocyclic compounds	C4H4N2O	-
164	AMW1936	(Z)-3-Octenoic acid, methyl ester	Aldehyde, Ketones, Esters	C9H16O2	-
165	AMW2277	Acetophenone	Aldehyde, Ketones, Esters	C8H8O	sweet, pungent, hawthorn, mimosa, almond, acacia
166	AMW0800	2H-Pyran, 3,6-dihydro-4-methyl-2-(2-methyl-1-propenyl)-	Terpenoids	C10H16O	green, weedy, cortex, herbal, diphenyl, narcissus, celery
167	AMW1724	2-Pyrrolidinone	Aldehyde, Ketones, Esters	C4H7NO	-
168	AMW1146	3-Hexen-1-ol, propanoate, (Z)-	Aldehyde, Ketones, Esters	C9H16O2	green, fresh, fruity, apple, pear, vegetable, melon, banana, peach

169	AMW3998	Ethanol, 2-[2-(ethenyloxy)ethoxy]-	Alcohol and amines	C6H12O3	-
170	AMW2746	1,1'-Biphenyl, 2,2',5,5'-tetramethyl-	Benzene and substituted derivatives	C16H18	-
171	AMW2433	1,4,7,-Cycloundecatriene, 1,5,9,9-tetramethyl-, Z,Z,Z-	Hydrocarbons	C15H24	-
172	AMW2257	Aminothiazole	Heterocyclic compounds	C3H4N2S	-
173	AMW4876	2-Heptenal, 2-propyl-	Aldehyde, Ketones, Esters	C10H18O	-
174	AMW0402	2-Butoxyethyl acetate	Aldehyde, Ketones, Esters	C8H16O3	sweet, fruity
175	AMW3882	Succinimide, N-(E-2-buten-1-one-1-yl)-	Alcohol and amines	C8H9NO3	-
176	AMW2670	Benzaldehyde, 3,4-dimethyl-	Aldehyde, Ketones, Esters	C9H10O	-
177	AMW2445	Decane, 2,5-dimethyl-	Hydrocarbons	C12H26	-
178	AMW4971	1(3H)-Isobenzofuranone	Aldehyde, Ketones, Esters	C8H6O2	sweet, tonka, coumarin, powdery, coconut, phenol, lactonic
179	AMW3405	2-Furanmethanol, tetrahydro-, acetate	Aldehyde, Ketones, Esters	C7H12O3	fruity, ethereal, rummy, brown, cooked, cognac, tequila, caramel, nutty
180	AMW5049	Benzene, 1,2-dimethoxy-4-(1-propenyl)-	Benzene and substituted derivatives	C11H14O2	spicy clove
181	AMW2646	1-Octyl trifluoroacetate	Aldehyde, Ketones, Esters	C10H17F3O 2	-
182	AMW4913	3-Acetamidofuran	Heterocyclic compounds	C6H7NO2	-
183	AMW1442	Benzene, 1,2,3-trimethyl-	Benzene and substituted derivatives	C9H12	-
184	AMW1245	3,4-Dimethyl-3-pyrrolin-2-one	Aldehyde, Ketones, Esters	C6H9NO	-
185	AMW3727	Hydroxylamine, O-(phenylmethyl)-	Alcohol and amines	C7H9NO	-
186	AMW2106	Linalool	Terpenoids	C10H18O	floral, green
187	AMW2967	1,2,3,1',2',3'-Hexamethyl-bicyclopentyl-2,2'-diene	Hydrocarbons	C16H26	-
188	AMW2779	4(3H)-Pyrimidinone, 3-methyl-	Aldehyde, Ketones, Esters	C5H6N2O	-
189	AMW0126	para-Anisaldehyde diethyl acetal	Benzene and substituted derivatives	C12H18O3	-
190	AMW0110	Succinic acid, 3-methylbut-2-yl 2-methylpent-3-yl ester	Aldehyde, Ketones, Esters	C15H28O4	-
191	AMW3329	2,5-Dihydroxy-4-methoxyacetophenone	Aldehyde, Ketones, Esters	C9H10O4	-
192	AMW2164	2,6-Difluorobenzyl bromide	Others	C7H5BrF2	-
193	AMW2841	1,4-Dioxane-2,6-dione	Aldehyde, Ketones, Esters	C4H4O4	-

194	AMW0022	(E)-2-Butenoic acid, 2-(methylenecyclopropyl)prop-2-yl ester	Aldehyde, Ketones, Esters	C11H16O2	-
195	AMW0121	5-Methyl-4-hexene-1-yl acetate	Aldehyde, Ketones, Esters	C9H16O2	-
196	AMW2344	N-(1-Cyano-1-methylethyl)isobutyramide	Alcohol and amines	C8H14N2O	-
197	AMW1675	methyl-Thiourea	Nitrogen compounds	C2H6N2S	-
198	AMW1135	2H-1,2-Oxaborin, 2,3,3-triethyl-3,6-dihydro-	Heterocyclic compounds	C10H19BO	-
199	AMW1332	3-Aminopyridine	Heterocyclic compounds	C5H6N2	-
200	AMW2133	4-(2,2,3-trimethyl-6-methylenecyclohexyl)-3-Buten-2-one	Terpenoids	C14H22O	-
201	AMW0446	Paramethadione	Aldehyde, Ketones, Esters	C7H11NO3	-
202	AMW0026	5-Ethoxy-2-ethoxymethyl-3,4-dihydro-2H-pyrrole	Heterocyclic compounds	C9H17NO2	-
203	AMW3801	1H-Tetrazole, 1,5-dimethyl-	Heterocyclic compounds	C3H6N4	-
204	AMW2246	3,6-dimethyl-1,4-Dioxane-2,5-dione	Aldehyde, Ketones, Esters	C6H8O4	-
205	AMW4223	Amine, dimethyl-2-phosphinoethyl-	Alcohol and amines	C4H12NP	-
206	AMW3502	Acetic acid, diethoxy-, ethyl ester	Aldehyde, Ketones, Esters	C8H16O4	-
207	AMW3724	3-Cyclohexene-1-carboxaldehyde, 1-methyl-	Aldehyde, Ketones, Esters	C8H12O	-
208	AMW3301	5-Nonanol	Alcohol and amines	C9H20O	-
209	AMW2756	Propane, 1,1'-[ethylidenebis(oxy)]bis[2-methyl-	Ethers	C10H22O2	-
210	AMW3942	N-Methyl-2-isopropoxycarbonylazetidine	Aldehyde, Ketones, Esters	C8H15NO2	-
211	AMW0562	6-methyl-3(2H)-Pyridazinone	Aldehyde, Ketones, Esters	C5H6N2O	-
212	AMW2400	Benzaldehyde dimethyl acetal	Aldehyde, Ketones, Esters	C9H12O2	green, earthy, root, nutty, vegetable, spinach, potato, fungal, almond, winey
213	AMW0654	1,3-dimethoxy-Benzene	Ethers	C8H10O2	acid, fruity, nutmeg, neroli
214	AMW0393	1-Octanol	Alcohol and amines	C8H18O	intense citrus, rose
215	AMW2843	Nonane, 3-methyl-5-propyl-	Hydrocarbons	C13H28	-
216	AMW5047	2,4-Pentanedione, 3-(phenylmethyl)-	Aldehyde, Ketones, Esters	C12H14O2	-
217	AMW0533	Octanal	Aldehyde, Ketones, Esters	C8H16O	lemon, citrus, green grass
218	AMW3136	.beta.-BERGAMOTENE	Terpenoids	C15H24	-

219	AMW0563	1-Pentadecene	Hydrocarbons	C15H30	-
220	AMW0978	Dodecanenitrile	Nitrogen compounds	C12H23N	citrus, orange, peel, metallic, spicy
221	AMW4371	Cyclohexanamine, N-hydroxy-	Alcohol and amines	C6H13NO	-
222	AMW2266	n-Butyl methacrylate	Aldehyde, Ketones, Esters	C8H14O2	-
223	AMW3875	Butan-2-ol, 3,3-dimethyl-1-(pyrrolidin-1-yl)-	Alcohol and amines	C10H21NO	-
224	AMW0494	BenzenAmine, N,N-dimethyl-	Alcohol and amines	C8H11N	-
225	AMW5023	Dodecane, 2,2,11,11-tetramethyl-	Hydrocarbons	C16H34	-
226	AMW4319	4,4'-Bitriazolyl	Heterocyclic compounds	C4H4N6	-
227	AMW0887	6-Nonynoic acid, methyl ester	Aldehyde, Ketones, Esters	C10H16O2	-
228	AMW0926	2-Nonen-1-ol	Alcohol and amines	C9H18O	sweet, fatty, melon, cucumber, vegetable
229	AMW1414	5-methyl-2-(1-methylethyl)-2-Cyclohexen-1-one	Aldehyde, Ketones, Esters	C10H16O	-
230	AMW0765	2,3-Dimethyldecane	Hydrocarbons	C12H26	-
231	AMW1264	2-phenoxy-1-Propanol	Alcohol and amines	C9H12O2	-
232	AMW2343	3,4-Dimethoxytoluene	Ethers	C9H12O2	-
233	AMW0955	2,7-Octadien-1-ol	Alcohol and amines	C8H14O	-
234	AMW3838	3H-Pyrazole, 3,4-diamino-	Heterocyclic compounds	C3H6N4	-
235	AMW3338	2H-Pyranmethanol, tetrahydro-2,5-dimethyl-	Alcohol and amines	C8H16O2	-
236	AMW1794	Hexanoic acid, butyl ester	Aldehyde, Ketones, Esters	C10H20O2	fruity, pineapple, berry, apple, juicy, green, winey, waxy, cognac, soapy
237	AMW0192	propyl-Benzene	Benzene and substituted derivatives	C9H12	-
238	AMW1668	3-methyl-3-phenyl-Azetidine	Nitrogen compounds	C10H13N	-
239	AMW1448	Cyclohexene, 2-ethenyl-1,3,3-trimethyl-	Hydrocarbons	C11H18	-
240	AMW4870	4-Acetylbutyric acid	Organic acid and Its derivatives	C6H10O3	-
241	AMW0697	3,4-dihydro-6-methyl-2H-Pyran	Heterocyclic compounds	C6H10O	-
242	AMW1250	3(2H)-Furanone, 4-methoxy-2,5-dimethyl-	Aldehyde, Ketones, Esters	C7H10O3	sweet, moldy, mushroom, vegetable, potato, burnt, sugar, nut skin, wasabi, caramel, fruity, brandy
243	AMW1384	Benzenepropanoic Acid, 4-hydroxy-	Organic acid and Its derivatives	C9H10O3	-

244	AMW4143	2-Ethylhexylamine, N,N-di(allyl)-	Alcohol and amines	C14H27N	-
245	AMW2504	Hexanoic acid, 2-methylbutyl ester	Aldehyde, Ketones, Esters	C11H22O2	ethereal
246	AMW3421	3,3'-Bifuran, 2,2',3,3'-tetrahydro-	Heterocyclic compounds	C8H10O2	-
247	AMW3280	2,4,8-Trimethyl-1,2,3,4-tetrahydroquinoline	Benzene and substituted derivatives	C12H17N	-
248	AMW3268	2,4-Dimethylsulfolane	Heterocyclic compounds	C6H12O2S	-
249	AMW1149	6-UndecylAmine	Alcohol and amines	C11H25N	-
250	AMW1506	2,5-Dihydroxy-4-isopropyl-2,4,6-cycloheptatrien-1-one	Aldehyde, Ketones, Esters	C10H12O3	-
251	AMW0802	decyl-Cyclohexane	Hydrocarbons	C16H32	-
252	AMW3722	(S)-(-)-2-(Methoxymethyl)-1-pyrrolidinecarboxaldehyde	Aldehyde, Ketones, Esters	C7H13NO2	-
253	AMW2019	chloro-Acetic acid, 2-phenylethyl ester	Aldehyde, Ketones, Esters	C10H11ClO2	-
254	AMW1818	DiSulfur compounds, dipropyl	Ethers	C6H14S2	sulfury, earthy, burnt, green, onion
255	AMW4084	2-Methyl-4-decanone	Aldehyde, Ketones, Esters	C11H22O	-
256	AMW4228	Quinoline, decahydro-	Heterocyclic compounds	C9H17N	-
257	AMW4799	Ethanol, 2-nitro-, propionate (ester)	Aldehyde, Ketones, Esters	C5H9NO4	-
258	AMW0866	3-(4-Hydroxyphenyl)propanal	Aldehyde, Ketones, Esters	C9H10O2	-
259	AMW1748	trans-4-tert-butylcycloheptanol	Alcohol and amines	C11H22O	-
260	AMW3146	2-tert-Butoxytetrahydrofuran	Heterocyclic compounds	C8H16O2	-
261	AMW4465	N-Cyclopropanecarbonylcyclopropanecarbohydrazide	Nitrogen compounds	C8H12N2O2	-
262	AMW2464	2(3H)-Furanone, 5-ethyldihydro-5-methyl-	Aldehyde, Ketones, Esters	C7H12O2	-
263	AMW0686	5-methyl-Nonane	Hydrocarbons	C10H22	-
264	AMW4788	Benzene, nitro-	Benzene and substituted derivatives	C6H5NO2	-
265	AMW2934	2-Amino-2-oxoethyl acetate	Aldehyde, Ketones, Esters	C4H7NO3	-
266	AMW2714	Dodecyl isobutyl ether	Ethers	C16H34O	-
267	AMW3122	Pyrimidine, 2-methoxy-	Heterocyclic compounds	C5H6N2O	-

268	AMW3220	Octane, 2-iodo-	Halogenated hydrocarbons	C8H17I	-
269	AMW1383	Phenol, 4-(2-propenyl)-	Benzene and substituted derivatives	C9H10O	phenol, medicinal, herbal
270	AMW2652	2-Cyclopenten-1-one, 2-hydroxy-3-methyl-	Aldehyde, Ketones, Esters	C6H8O2	caramel, maple, syrup
271	AMW4774	Thiophene, 2-(methylthio)-	Heterocyclic compounds	C5H6S2	-
272	AMW3514	Cyclobutylamine, N-(2-methylpropionyl)-	Alcohol and amines	C8H15NO	-
273	AMW1195	Furaneol	Aldehyde, Ketones, Esters	C6H8O3	sweet, cotton, candy, caramel, strawberry, sugar
274	AMW3745	Bicyclo[2.2.1]heptan-2-one, 7,7-dimethyl-	Aldehyde, Ketones, Esters	C9H14O	-
275	AMW0886	5-methyl-2-(methylthio)-4(1H)-Pyrimidinone	Aldehyde, Ketones, Esters	C6H8N2OS	-
276	AMW0029	2-Amino-2-methyl-but-3-enoic Acid	Organic acid and Its derivatives	C5H9NO2	-
277	AMW0228	Caprolactam	Alcohol and amines	C6H11NO	amine, spicy
278	AMW3096	2-Oxazolidinone,4,4-dimethyl-	Aldehyde, Ketones, Esters	C5H9NO2	-
279	AMW3948	Carbonic acid, neopentyl 2-ethylhexyl ester	Aldehyde, Ketones, Esters	C14H28O3	-
280	AMW0099	6-methyl-7-Oxa-8-azabicyclo[4.2.1]non-8-ene	Heterocyclic compounds	C8H13NO	-
281	AMW2369	Benzoic acid, methyl ester	Aldehyde, Ketones, Esters	C8H8O2	phenol, wintergreen, almond, floral, canga
282	AMW4900	1-Heptanol, 2-propyl-	Alcohol and amines	C10H22O	-
283	AMW2704	.alpha.-Cadinol	Terpenoids	C15H26O	herbal, woody
284	AMW3944	2,2-Dimethylpropionic acid, 4-methylpentyl ester	Aldehyde, Ketones, Esters	C11H22O2	-
285	AMW4308	Cyclobutanone, 2,2,4,4-tetramethyl-	Aldehyde, Ketones, Esters	C8H14O	-
286	AMW1572	2-methyl-2-Undecene	Hydrocarbons	C12H24	-
287	AMW0410	isothiocyanato-Cyclohexane	Nitrogen compounds	C7H11NS	-
288	AMW2681	4-Methyleneisophorone	Aldehyde, Ketones, Esters	C10H14O	-
289	AMW3958	2-Azetidinone, 3,3-dimethyl-	Aldehyde, Ketones, Esters	C5H9NO	-
290	AMW4336	Nitric acid, octyl ester	Aldehyde, Ketones, Esters	C8H17NO3	-
291	AMW2151	δ-valerolactone6-methyl-	Aldehyde, Ketones, Esters	C6H10O2	creamy, fruity, coconut
292	AMW0148	4-Amino-4,5(1H)-dihydro-1,2,4-triazole-5-one	Aldehyde, Ketones, Esters	C2H4N4O	-
293	AMW1295	2-Chloro-2-methylhexane	Halogenated hydrocarbons	C7H15Cl	-

294	AMW4794	2-Butynamide, N,N-dimethyl-	Alcohol and amines	C6H9NO	-
295	AMW4035	N-(2-Cyano-1-methylvinyl)acetamide	Alcohol and amines	C6H8N2O	-
296	AMW0712	1,9-Decadiene	Hydrocarbons	C10H18	-
297	AMW0405	2-Undecanone	Aldehyde, Ketones, Esters	C11H22O	waxy, fruity, creamy, fatty, orris, floral
298	AMW2633	2-Undecene, 3-methyl-, (E)-	Hydrocarbons	C12H24	-
299	AMW1092	Ethanone, 1-(4,5-dihydro-2-thiazolyl)-	Aldehyde, Ketones, Esters	C5H7NOS	corn, chip, taco, potato, toasted, bread, nutty
300	AMW4694	3,4-Diethyl-3-hexene	Hydrocarbons	C10H20	-
301	AMW1395	4-Aminopyridine	Heterocyclic compounds	C5H6N2	-
302	AMW0303	m-Chloroaniline	Alcohol and amines	C6H6ClN	-
303	AMW1151	1,3-Cyclohexanediamine	Alcohol and amines	C6H14N2	-
304	AMW1503	1-(2-furanyl)-1-Octanone	Aldehyde, Ketones, Esters	C12H18O2	-
305	AMW3454	(4aS,9aR)-3,5,5,9-Tetramethyl-2,4a,5,6,7,9a-hexahydro-1H-benzo[7]annulene	Heterocyclic compounds	C15H24	-
306	AMW1948	Undecane, 2-methyl-	Hydrocarbons	C12H26	-
307	AMW0416	Dodecane	Hydrocarbons	C12H26	alkane
308	AMW1667	2H-Indol-2-one, 1,3-dihydro-	Aldehyde, Ketones, Esters	C8H7NO	-
309	AMW1793	Hexanoic acid, propyl ester	Aldehyde, Ketones, Esters	C9H18O2	sweet, fruity, juicy, pineapple, green, tropical
310	AMW2762	Isopropoxycarbamic acid, ethyl ester	Aldehyde, Ketones, Esters	C6H13NO3	-
311	AMW3380	4-Piperidone	Aldehyde, Ketones, Esters	C5H9NO	-
312	AMW1841	N,N-diethyl-Urea	Nitrogen compounds	C5H12N2O	-
313	AMW4759	1,5-Cyclooctadiene, 1,5-dimethyl-	Hydrocarbons	C10H16	-
314	AMW3934	1H,5H,7H,11H-Dipyrazolo[1,2-a:1',2'-d][1,2,4,5]tetrazine, tetrahydro-	Heterocyclic compounds	C8H16N4	-
315	AMW0180	3-Ethylcyclopentanone	Aldehyde, Ketones, Esters	C7H12O	-
316	AMW2077	2,5-dimethyl-2,4-Hexadiene	Hydrocarbons	C8H14	-
317	AMW0751	2(4H)-Benzofuranone, 5,6,7,7a-tetrahydro-4,4,7a-trimethyl-,	Aldehyde, Ketones, Esters	C11H16O2	musky, coumarin

		(R)-			
318	AMW4006	1,2,4-Triazin-3-amine, 5,6-dimethyl-	Alcohol and amines	C5H8N4	-
319	AMW4309	2H-Inden-2-one, 1,3-dihydro-	Aldehyde, Ketones, Esters	C9H8O	-
320	AMW3138	5-Hydroxy-2-pyrimidinecarbonitrile	Nitrogen compounds	C5H3N3O	-
321	AMW4328	8-Azabicyclo[3.2.1]octan-3-ol, 8-methyl-, endo-	Alcohol and amines	C8H15NO	-
322	AMW0135	N-(2-Pyridinylmethyl)-1-butanamine, N-acetyl-	Alcohol and amines	C12H18N2 O	-
323	AMW1021	2,4,6-trimethyl-Heptane	Hydrocarbons	C10H22	-
324	AMW3666	3-Methyl-2-butenoic acid, cyclobutyl ester	Aldehyde, Ketones, Esters	C9H14O2	-
325	AMW3609	1H-1,2,4-Triazole	Heterocyclic compounds	C2H3N3	-
326	AMW2431	1,3-Cyclohexanedione	Aldehyde, Ketones, Esters	C6H8O2	-
327	AMW3824	2,3-Butanedione, monooxime	Aldehyde, Ketones, Esters	C4H7NO2	-
328	AMW0432	N,N-dimethyl-1-Tetradecanamine	Alcohol and amines	C16H35N	-
329	AMW3404	Propanoic acid, 2-(aminooxy)-	Organic acid and Its derivatives	C3H7NO3	-
330	AMW3155	Phenylglyoxylic acid, 2-methylbutyl ester	Aldehyde, Ketones, Esters	C13H16O3	-
331	AMW4506	Dimethyl-1,2,3-oxadiazol-3-one	Aldehyde, Ketones, Esters	C4H6N2O2	-
332	AMW3695	6-Undecanol	Alcohol and amines	C11H24O	-
333	AMW1049	5-methyl-5-(2-methylpropyl)-2,4-Imidazolidinedione	Aldehyde, Ketones, Esters	C8H14N2O 2	-
334	AMW2657	2-Isobutoxyethyl acetate	Aldehyde, Ketones, Esters	C8H16O3	-
335	AMW1925	2-Decanone	Aldehyde, Ketones, Esters	C10H20O	orange, floral, fatty, peach
336	AMW3866	3-Aminopyrazole	Heterocyclic compounds	C3H5N3	-
337	AMW2579	Propanoic acid, 2-hydroxyethyl ester	Aldehyde, Ketones, Esters	C5H10O3	-
338	AMW0749	Naphthalene, decahydro-4a-methyl-1-methylene-7-(1-methylethenyl)-, [4aR-(4a.alpha.,7.alpha.,8a.beta.)]-	Terpenoids	C15H24	herbal
339	AMW2576	Dodecane, 5-methyl-	Hydrocarbons	C13H28	-

340	AMW3919	1,4-Dimethyl-2-piperidone	Aldehyde, Ketones, Esters	C7H13NO	-
341	AMW0918	6-hydroxy-2-Hexanone	Aldehyde, Ketones, Esters	C6H12O2	-
342	AMW3485	Acetonitrile, 2,2'-iminobis-	Nitrogen compounds	C4H5N3	-
343	AMW3007	Oxalic acid, butyl cyclobutyl ester	Aldehyde, Ketones, Esters	C10H16O4	-
344	AMW1778	Aniline	Alcohol and amines	C6H7N	-
345	AMW3104	Butyl isocyanatoacetate	Aldehyde, Ketones, Esters	C7H11NO3	-
346	AMW2490	Benzenemethanol, .alpha.,.alpha.-dimethyl-	Alcohol and amines	C9H12O	-
347	AMW4453	4,5-Dihydro-2(1H)-pentalenone	Aldehyde, Ketones, Esters	C8H8O	-
348	AMW1578	3,4-Dimethylcyclohexanol	Alcohol and amines	C8H16O	-
349	AMW0250	5-Heptenal, 2,6-dimethyl-	Aldehyde, Ketones, Esters	C9H16O	fresh, ozonous, melon, fresh air, sweet, green
350	AMW1820	Tridecane	Hydrocarbons	C13H28	alkane
351	AMW0409	nitro-Cyclohexane	Nitrogen compounds	C6H11NO2	-
352	AMW2956	2,2'-Bifuran, octahydro-	Heterocyclic compounds	C8H14O2	-
353	AMW0728	2-Acetylcyclopentanone	Aldehyde, Ketones, Esters	C7H10O2	-
354	AMW4719	Cyclooctane, methyl-	Hydrocarbons	C9H18	-
355	AMW3067	Oxazolidin-2-one	Aldehyde, Ketones, Esters	C3H5NO2	-
356	AMW1441	2,3,6,7-tetramethyl-Octane	Hydrocarbons	C12H26	-
357	AMW0555	4-methyl-1-Decene	Hydrocarbons	C11H22	-
358	AMW0622	2,2,4-trimethyl-1,3-Pentanediol	Alcohol and amines	C8H18O2	-
359	AMW2068	N,N-dibutyl-Formamide	Alcohol and amines	C9H19NO	-
360	AMW1302	2-Phenylpropenal	Aldehyde, Ketones, Esters	C9H8O	-
361	AMW1235	2-butyl-1-Octanol	Alcohol and amines	C12H26O	-
362	AMW1063	2-Methyl-3-furanthiol	Alcohol and amines	C5H6OS	sulfury, meaty, fishy, metallic
363	AMW1702	1,3-Hexadiene, 3-ethyl-2-methyl-	Hydrocarbons	C9H16	nutty
364	AMW4416	4,4-Dimethyl-2-pentanol, 2-methylpropionate	Aldehyde, Ketones, Esters	C11H22O2	-
365	AMW4866	Pyrrolidine, 1-acetyl-	Heterocyclic compounds	C6H11NO	bread, dark, malty, grassy, hay, perfumy, currant, sweet

366	AMW3105	2,4-Dimethyl-2-oxazoline-4-methanol	Alcohol and amines	C6H11NO2	-
367	AMW2678	2-Pyrazoline, 1-isobutyl-3-methyl-	Heterocyclic compounds	C8H16N2	-
368	AMW0907	Decyl acrylate	Aldehyde, Ketones, Esters	C13H24O2	-
369	AMW0064	Oxalic acid, neopentyl propyl ester	Aldehyde, Ketones, Esters	C10H18O4	-
370	AMW4708	2-Isopropylpyrazine	Heterocyclic compounds	C7H10N2	minty, green, nutty, honey
371	AMW2341	Dodecane, 3-methyl-	Hydrocarbons	C13H28	-
372	AMW2287	.gamma.-Terpinene	Terpenoids	C10H16	oily, smoky
373	AMW1114	Dodecane, 2,6,11-trimethyl-	Hydrocarbons	C15H32	-
374	AMW3056	1,2,4-Triazolo[4,3-b]pyridazine, 6-methyl-	Heterocyclic compounds	C6H6N4	-
375	AMW0226	Ethyl cyanoacetate	Aldehyde, Ketones, Esters	C5H7NO2	-
376	AMW4840	Benzyl nitrile	Nitrogen compounds	C8H7N	green, crushed leaves
377	AMW2992	2-Butanone, 3,3-dimethyl-1-thiocyanato-	Aldehyde, Ketones, Esters	C7H11NOS	-
378	AMW3043	Cyclohexane, 1-ethyl-2-propyl-	Hydrocarbons	C11H22	-
379	AMW2803	Pentanoic acid, 2-methyl-, anhydride	Organic acid and Its derivatives	C12H22O3	-
380	AMW4280	Piperidine, 1-pentyl-	Heterocyclic compounds	C10H21N	-
381	AMW4711	Pyridine, 2,4,6-trimethyl-	Heterocyclic compounds	C8H11N	-
382	AMW3223	1,3-Diacetin	Aldehyde, Ketones, Esters	C7H12O5	-
383	AMW4899	Pyridine, 2-pentyl-	Heterocyclic compounds	C10H15N	fatty, tallow, green, pepper, mushroom, herbal
384	AMW1509	dihydro-3-methylene-2(3H)-Furanone	Aldehyde, Ketones, Esters	C5H6O2	-
385	AMW2023	1,2,4,5-tetramethyl-6-methylene-Spiro[2.4]heptane	Hydrocarbons	C12H20	-
386	AMW2793	Carbonic acid, isobutyl 4-isopropylphenyl ester	Aldehyde, Ketones, Esters	C14H20O3	-
387	AMW4706	3-Hepten-2-one, 5-methyl-	Aldehyde, Ketones, Esters	C8H14O	Green
388	AMW0217	2(3H)-Furanone, dihydro-5-propyl-	Aldehyde, Ketones, Esters	C7H12O2	sweet, coconut, nutty, caramel, tonka, hay
389	AMW0207	1,8,11,14-Heptadecatetraene, (Z,Z,Z)-	Hydrocarbons	C17H28	costus
390	AMW4922	8-Hydroxy-2-octanone	Aldehyde, Ketones, Esters	C8H16O2	-
391	AMW2367	Hexanoic acid, 2-methylpropyl ester	Aldehyde, Ketones, Esters	C10H20O2	fruity, pineapple, green, apple skin, sour, tropical, peach, earthy

392	AMW0209	Tridecanal	Aldehyde, Ketones, Esters	C13H26O	fresh, clean, aldehydic, soapy, citrus, petal, waxy, grapefruit, peel
393	AMW4691	1-Pentanol, 4-amino-	Alcohol and amines	C5H13NO	-
394	AMW1001	1,3-Dimethyl-1H-pyrazole-4-carbaldehyde	Aldehyde, Ketones, Esters	C6H8N2O	-
395	AMW3990	Carbonic acid, monoamide, N-(2-pentyl)-N-butyl-, propargyl ester	Aldehyde, Ketones, Esters	C13H23NO 2	-
396	AMW0500	Diphenylamine	Alcohol and amines	C12H11N	sweet, floral, faint
397	AMW5038	2-Dodecenal, (E)-	Hydrocarbons	C12H22O	fatty, pungent
398	AMW0774	Nonane, 5-methyl-5-propyl-	Hydrocarbons	C13H28	-
399	AMW4085	1H-Imidazole-2-carboxaldehyde, 1-methyl-	Aldehyde, Ketones, Esters	C5H6N2O	-
400	AMW4792	1-Pyrrolidinecarboxaldehyde	Aldehyde, Ketones, Esters	C5H9NO	-
401	AMW3142	Nonane, 5-propyl-	Hydrocarbons	C12H26	-
402	AMW0914	[1aR-(1a.alpha.,7.alpha.,7a.beta.,7b.alpha.)]-1a,2,3,5,6,7,7a,7b-octahydro-1,1,4,7-tetramethyl-1H-Cycloprop[e]azulene	Terpenoids	C15H24	-
403	AMW0730	1,2-dimethyl-Cyclohexene	Hydrocarbons	C8H14	-
404	AMW2853	Hexahydro-1,3,5-trinitroso-1,3,5-triazine	Heterocyclic compounds	C3H6N6O3	-
405	AMW0421	Undecanal	Aldehyde, Ketones, Esters	C11H22O	waxy, soapy, floral, aldehydic, citrus, green, fatty, fresh
406	AMW3533	Bicyclo(3.1.1)heptane-2,3-diol, 2,6,6-trimethyl-	Alcohol and amines	C10H18O2	-
407	AMW1225	4-Amino-2(1H)-pyridinone	Aldehyde, Ketones, Esters	C5H6N2O	-
408	AMW4377	l-Alanine, n-propargyloxycarbonyl-, pentyl ester	Aldehyde, Ketones, Esters	C12H19NO 4	-
409	AMW1458	4-methyl-3-Pentenal	Aldehyde, Ketones, Esters	C6H10O	-
410	AMW1933	2,4-dimethyl-Cyclohexanol	Alcohol and amines	C8H16O	-
411	AMW3776	4H-1,2,4-Triazole, 4-ethyl-	Heterocyclic compounds	C4H7N3	-
412	AMW4491	3-Butylisobenzofuran-1(3H)-one	Aldehyde, Ketones, Esters	C12H14O2	herbal, phenol, celery
413	AMW3455	Tetradecane, 6,9-dimethyl-	Hydrocarbons	C16H34	-

414	AMW0277	Phenylglyoxal	Aldehyde, Ketones, Esters	C8H6O2	-
415	AMW3876	Oxirane, dodecyl-	Heterocyclic compounds	C14H28O	-
416	AMW4253	1,2,4-Cyclopentanetrione, 3-methyl-	Aldehyde, Ketones, Esters	C6H6O3	-
417	AMW4267	Vinyl 2-ethylhexanoate	Aldehyde, Ketones, Esters	C10H18O2	-
418	AMW3115	5-Ethyl-2-furaldehyde	Aldehyde, Ketones, Esters	C7H8O2	-
419	AMW3014	Acetic acid, [(1,1-dimethylethyl)thio]-	Organic acid and Its derivatives	C6H12O2S	-
420	AMW2364	4-Methylpentyl 4-methylpentanoate	Aldehyde, Ketones, Esters	C12H24O2	fruity, waxy, soapy, herbal
421	AMW5110	1-Tetradecanamine	Alcohol and amines	C14H31N	-
422	AMW4131	2,2-Dimethyl-3-methoxy-cyclopropane-1-carboxylic acid	Organic acid and Its derivatives	C7H12O3	-
423	AMW1567	Allylidenecyclohexane	Hydrocarbons	C9H14	-
424	AMW2083	1-Pentadecyne	Hydrocarbons	C15H28	-
425	AMW2919	Decane, 2,9-dimethyl-	Hydrocarbons	C12H26	-
426	AMW0927	3-methyl-2(5H)-Furanone	Aldehyde, Ketones, Esters	C5H6O2	-
427	AMW4111	Pyrazole-4-carboxaldehyde, 1-ethyl-5-methyl-	Aldehyde, Ketones, Esters	C7H10N2O	-
428	AMW0473	2-Acetyl-5-methylfuran	Heterocyclic compounds	C7H8O2	strong, musty, nutty, hay, coconut, coumarin, milky
429	AMW2987	Butane-2-one, 3-methyl-3-(2-oxopropylamino)-	Aldehyde, Ketones, Esters	C8H15NO2	-
430	AMW1582	4'-Butoxyacetophenone	Aldehyde, Ketones, Esters	C12H16O2	-
431	AMW4941	3-Hexanol, 1,5-dimethoxy-2,4-dimethyl-	Alcohol and amines	C10H22O3	-
432	AMW2236	(1-hydroxycyclohexyl)phenyl-Methanone	Aldehyde, Ketones, Esters	C13H16O2	-
433	AMW4395	2-Pyrimidinecarbonitrile	Nitrogen compounds	C5H3N3	-
434	AMW3996	(2S,4aS,5R,8aR)-2,5-Dipropyldecahydroquinoline	Heterocyclic compounds	C15H29N	-
435	AMW4469	Hexyl 4-methoxyphenyl ether	Ethers	C13H20O2	-
436	AMW3622	Cyclohexanemethanol, 4-methyl-, cis-	Alcohol and amines	C8H16O	-
437	AMW0974	1-chloro-Tetradecane	Halogenated hydrocarbons	C14H29Cl	-
438	AMW4303	1,3-Dioxolan-2-one, 4,5-bis(methylene)-	Aldehyde, Ketones, Esters	C5H4O3	-
439	AMW2260	4-t-Butylcyclohexathiazole	Heterocyclic compounds	C11H17NS	-

440	AMW3804	1-(1-Cyclopropyl-pentyl)piperidine	Heterocyclic compounds	C13H25N	-
441	AMW3281	Oxalic acid, allyl ethyl ester	Aldehyde, Ketones, Esters	C7H10O4	-
442	AMW2429	δ-valerolactone6-propyl-	Aldehyde, Ketones, Esters	C8H14O2	coconut
443	AMW0128	3-Ethyl-2,6,10-trimethylundecane	Hydrocarbons	C16H34	-
444	AMW1489	1-Nonene, 4,6,8-trimethyl-	Hydrocarbons	C12H24	-
445	AMW0863	2,4-Dithiapentane 2,2-dioxide	Ethers	C3H8O2S2	-
446	AMW4763	3(2H)-Furanone, 4-hydroxy-5-methyl-	Aldehyde, Ketones, Esters	C5H6O3	Caramel
447	AMW3997	4-Methylpiperidine-2-carboxylic acid, methyl ester	Aldehyde, Ketones, Esters	C8H15NO2	-
448	AMW4413	4-Hepten-3-one, 5-methyl-	Aldehyde, Ketones, Esters	C8H14O	-
449	AMW4747	3-(Methylthio)propanoic acid methyl ester	Aldehyde, Ketones, Esters	C5H10O2S	sulfury, vegetable, onion, sweet, garlic, tomato
450	AMW0946	Nonaneperoxoic acid, 1,1-dimethylethyl ester	Aldehyde, Ketones, Esters	C13H26O3	-
451	AMW4865	p-Mentha-1(7),2-dien-8-ol	Terpenoids	C10H16O	-
452	AMW2589	Pentanoic acid, 2-methylpropyl ester	Aldehyde, Ketones, Esters	C9H18O2	ethereal, fruity
453	AMW3530	Heptane, 2,2,3,3,5,6,6-heptamethyl-	Hydrocarbons	C14H30	-
454	AMW4222	2(1H)-Pyridinone	Aldehyde, Ketones, Esters	C5H5NO	-
455	AMW2228	1-(1-cyclohexen-1-yl)-Ethanone	Aldehyde, Ketones, Esters	C8H12O	-
456	AMW4755	3,4-Octadiene, 2,2,7,7-tetramethyl-	Hydrocarbons	C12H22	-
457	AMW1372	octahydro-1H-Indene	Benzene and substituted derivatives	C9H16	-
458	AMW3768	Urea, 2-propenyl-	Nitrogen compounds	C4H8N2O	-
459	AMW1931	2(3H)-Furanone, 5-ethyldihydro-	Aldehyde, Ketones, Esters	C6H10O2	sweet, caramel
460	AMW4183	4'-Propoxy-2-methylpropiophenone	Aldehyde, Ketones, Esters	C13H18O2	-
461	AMW0159	BenzAldehyde	Aldehyde, Ketones, Esters	C7H6O	sweet, bitter, almond, cherry
462	AMW4494	Benzenamine, 4-propyl-	Alcohol and amines	C9H13N	-
463	AMW0815	3-methyl-Tetradecane	Hydrocarbons	C15H32	-
464	AMW2497	7-Exo-ethyl-5-methyl-6,8-dioxabicyclo[3.2.1]oct-3-ene	Heterocyclic compounds	C9H14O2	-
465	AMW4734	3-Octen-2-ol, 2-methyl-, (Z)-	Alcohol and amines	C9H18O	-

466	AMW4773	Acetic acid, phenyl ester	Aldehyde, Ketones, Esters	C8H8O2	phenol, medicinal, animalic, resinous, castoreum, woody, smoky, burnt
467	AMW5097	1-Naphthalenol, 1,2,3,4-tetrahydro-2,5,8-trimethyl-	Benzene and substituted derivatives	C13H18O	-
468	AMW4406	2-Hydroxy-4,6-dimethylbenzaldehyde	Aldehyde, Ketones, Esters	C9H10O2	-
469	AMW4811	1-Nonen-4-ol	Alcohol and amines	C9H18O	-
470	AMW0005	trans,trans- and trans,cis-1,8-Dimethylspiro[5.5]undecane	Hydrocarbons	C13H24	-
471	AMW3500	Precocene I	Heterocyclic compounds	C12H14O2	-
472	AMW4949	Phenol, p-tert-butyl-	Benzene and substituted derivatives	C10H14O	oakmoss, leathery
473	AMW3663	1-Heptanol, 6-methyl-	Alcohol and amines	C8H18O	-
474	AMW3357	Benzene, 1-ethyl-3-(1-methylethyl)-	Benzene and substituted derivatives	C11H16	-
475	AMW2898	3,5-Dimethylpyrazole-1-carboxamide	Alcohol and amines	C6H9N3O	-
476	AMW0612	Hexanoic Acid	Organic acid and Its derivatives	C6H12O2	rose, geranium, cheese, fatty
477	AMW0734	3,5-Heptanedione, 4-ethyl-2,2,6,6-tetramethyl-	Aldehyde, Ketones, Esters	C13H24O2	-
478	AMW0149	2-Methylthiolane, S,S-dioxide	Others	C5H10O2S	-
479	AMW1213	Furan, 2-pentyl-	Heterocyclic compounds	C9H14O	fruity, green, earthy, beany, vegetable, metallic
480	AMW0514	.beta.-Myrcene	Terpenoids	C10H16	musty, balsamic, spice
481	AMW2623	6,8-Dioxabicyclo[3.2.1]octane, 7-ethyl-5-methyl-, (1R-exo)-	Heterocyclic compounds	C9H16O2	-
482	AMW1122	Phenol, 2-butyl-	Benzene and substituted derivatives	C10H14O	-
483	AMW4559	1,3-Butanediol	Alcohol and amines	C4H10O2	-
484	AMW4814	Hotrienol	Terpenoids	C10H16O	sweet, tropical, ocimene, fennel, ginger, myrcene
485	AMW0062	Oxalic acid, ethyl 2-isopropylphenyl ester	Aldehyde, Ketones, Esters	C13H16O4	-
486	AMW4895	4H-Pyran-4-one, 3,5-dihydroxy-2-methyl-	Aldehyde, Ketones, Esters	C6H6O4	caramel
487	AMW0102	Ethyl 3-methylbut-3-enyl carbonate	Aldehyde, Ketones, Esters	C8H14O3	-
488	AMW0550	2,6-diethyl-Pyrazine	Heterocyclic compounds	C8H12N2	nutty, hazelnut
489	AMW0158	Benzyl Alcohol	Alcohol and amines	C7H8O	floral, rose, phenol, balsamic
490	AMW4405	Tetradecane, 2,5-dimethyl-	Hydrocarbons	C16H34	-
491	AMW3715	1-Methyl-2,4,5-trioxoimidazolidine	Heterocyclic compounds	C4H4N2O3	-

492	AMW4843	Benzene, 1,3-diethyl-5-methyl-	Benzene and substituted derivatives	C11H16	-
493	AMW0911	cis-7-Decen-1-al	Aldehyde, Ketones, Esters	C10H18O	citrus, aldehydic, cucumber
494	AMW0006	threo-2,5-Dimethyl-2-(2-methyl-2-tetrahydrofuryl)tetrahydrofuran	Heterocyclic compounds	C11H20O2	-
495	AMW1693	2,3-dichloro-Benzenamine	Alcohol and amines	C6H5Cl2N	-
496	AMW1603	Cyclohexene, 1-methyl-4-(1-methylethylidene)-	Terpenoids	C10H16	citrus, pine
497	AMW4749	1,3-Dithiane	Heterocyclic compounds	C4H8S2	garlic, onion, roasted, boiled
498	AMW3148	2-Cyano-2-isopropyl-3-methylbutanoic acid	Organic acid and Its derivatives	C9H15NO2	-
499	AMW2783	Decane, 5-ethyl-5-methyl-	Hydrocarbons	C13H28	-
500	AMW2157	1,8-Ethylenenaphthalene	Benzene and substituted derivatives	C12H10	-
501	AMW1136	2-Pentoxy-tetrahydropyran	Heterocyclic compounds	C10H20O2	-
502	AMW2686	1-(1-Methoxypropan-2-yloxy)propan-2-yl acetate	Aldehyde, Ketones, Esters	C9H18O4	-
503	AMW3216	1H-Pyrrole, 3-ethyl-2,4,5-trimethyl-	Heterocyclic compounds	C9H15N	-
504	AMW3347	Decane, 3-bromo-	Halogenated hydrocarbons	C10H21Br	-
505	AMW0369	2-Propanol, 1,1'-oxybis-	Alcohol and amines	C6H14O3	mild alcoholic
506	AMW1617	3-Octanol	Alcohol and amines	C8H18O	earthy, mushroom, herbal, melon, citrus, woody, spicy, minty
507	AMW4458	1H-Pyrazole, 3-ethyl-4,5-dihydro-	Heterocyclic compounds	C5H10N2	-
508	AMW4363	4-Amino-3-hydroxytetrahydrothiophene 1,1-dioxide	Heterocyclic compounds	C4H9NO3S	-
509	AMW0079	4-Ethylbenzoic acid, 2-methylbutyl ester	Aldehyde, Ketones, Esters	C14H20O2	-
510	AMW2639	Cyclopentanecarboxylic acid, ethenyl ester	Aldehyde, Ketones, Esters	C8H12O2	-
511	AMW1229	2,6,10-Trimethyltridecane	Hydrocarbons	C16H34	-
512	AMW4258	2,2-Dimethoxypropionamide	Alcohol and amines	C5H11NO3	-
513	AMW1211	1-(3-methylbutyl)-Cyclopentene	Hydrocarbons	C10H18	-
514	AMW3371	4-Formyl-3,5-dimethyl-1H-pyrrole-2-carbonitrile	Heterocyclic compounds	C8H8N2O	-
515	AMW3091	2,4-Imidazolidinedione, 5,5-dimethyl-	Aldehyde, Ketones, Esters	C5H8N2O2	-
516	AMW4049	(3R)-(+)-3-Acetamidopyrrolidine	Heterocyclic compounds	C6H12N2O	-

517	AMW3886	2-Furancarboxylic acid	Organic acid and Its derivatives	C5H4O3	acid, caramel, earthy
518	AMW1233	(3R,6S)-2,2,6-Trimethyl-6-vinyltetrahydro-2H-pyran-3-ol	Alcohol and amines	C10H18O2	woody
519	AMW3554	Hexadecane, 4-methyl-	Hydrocarbons	C17H36	-
520	AMW2680	2,4,6-Trimethyl-1-nonene	Hydrocarbons	C12H24	-
521	AMW5109	Propanedioic acid, phenyl-	Organic acid and Its derivatives	C9H8O4	-
522	AMW3767	Heptanonitrile	Nitrogen compounds	C7H13N	-
523	AMW0665	Cyclohexa-2,4-dienylmethanol	Alcohol and amines	C7H10O	-
524	AMW0335	1-chloro-Butane	Halogenated hydrocarbons	C4H9Cl	-
525	AMW3041	5-Methylenehydantoin	Nitrogen compounds	C4H4N2O2	-
526	AMW2561	1H-Indene, 2,3-dihydro-5-methyl-	Benzene and substituted derivatives	C10H12	-
527	AMW2323	n-Caprylic acid isobutyl ester	Aldehyde, Ketones, Esters	C12H24O2	fruity, green, oily, floral
528	AMW2764	Indolin-2-one, 1-methyl-3-t-butyl-	Aldehyde, Ketones, Esters	C13H17NO	-
529	AMW3675	Valeric anhydride	Organic acid and Its derivatives	C10H18O3	-
530	AMW3378	Heptane, 4-ethyl-	Hydrocarbons	C9H20	-
531	AMW1173	2-Piperidinemethanol	Alcohol and amines	C6H13NO	-
532	AMW0368	5-Hepten-2-one, 6-methyl-	Aldehyde, Ketones, Esters	C8H14O	herbal, green, citrus, musty, lemon grass
533	AMW2938	Tridecane, 7-methyl-	Hydrocarbons	C14H30	-
534	AMW1184	4(1H)-Pyridinone, 2,3-dihydro-1-methyl-	Aldehyde, Ketones, Esters	C6H9NO	-
535	AMW1100	1,3-Cyclohexadiene, 5-butyl-	Hydrocarbons	C10H16	-
536	AMW3964	l-Alanine, N-ethoxycarbonyl-, hexyl ester	Aldehyde, Ketones, Esters	C12H23NO4	-
537	AMW3864	2-Thiophenecarboxylic acid, 2-methylbutyl ester	Aldehyde, Ketones, Esters	C10H14O2S	-
538	AMW3250	4-Octen-3-one, 6-ethyl-7-hydroxy-	Aldehyde, Ketones, Esters	C10H18O2	-
539	AMW1669	2-Propanol, 1,3-bis(dimethylamino)-	Alcohol and amines	C7H18N2O	-
540	AMW5050	1,1'-Biphenyl, 4-methyl-	Benzene and substituted derivatives	C13H12	-
541	AMW3499	(Z)-(Z)-Hex-3-en-1-yl 2-methylbut-2-enoate	Aldehyde, Ketones, Esters	C11H18O2	green, floral, herbal, apple, pear

542	AMW4125	Butanedioic acid, 2-hydroxy-2-methyl-, (S)-	Organic acid and Its derivatives	C5H8O5	-
543	AMW0717	2-Undecanol	Alcohol and amines	C11H24O	fresh, waxy, clean, cloth, cotton, sarsaparilla
544	AMW4854	Benzene, pentyl-	Benzene and substituted derivatives	C11H16	-
545	AMW1178	11-Tetradecen-1-ol, (E)-	Alcohol and amines	C14H28O	-
546	AMW2637	Nonane, 5-(2-methylpropyl)-	Hydrocarbons	C13H28	-
547	AMW3601	Barbituric acid	Organic acid and Its derivatives	C4H4N2O3	-
548	AMW4160	Dodecane, 2,5-dimethyl-	Hydrocarbons	C14H30	-
549	AMW1432	(Z,E)-9,12-Tetradecadien-1-ol	Alcohol and amines	C14H26O	-
550	AMW2990	2-Pyrazoline, 1-isopropyl-3,4-dimethyl-	Heterocyclic compounds	C8H16N2	-
551	AMW4905	Decane, 2,2,3-trimethyl-	Hydrocarbons	C13H28	-
552	AMW3215	1-Cyclopropyl-2-(3,4-dimethoxyphenyl)ethanol	Alcohol and amines	C13H18O3	-
553	AMW5118	Furazandiamine	Alcohol and amines	C2H4N4O	-
554	AMW1291	Nonane, 2,2,4,4,6,8,8-heptamethyl-	Hydrocarbons	C16H34	-
555	AMW0065	Oxalic acid, isobutyl neopentyl ester	Aldehyde, Ketones, Esters	C11H20O4	-
556	AMW1731	2-methyl-5-(1-methylethenyl)-Cyclohexanol	Terpenoids	C10H18O	minty, menthol, spearmint, herbal
557	AMW0651	Mequinol	Alcohol and amines	C7H8O2	phenol
558	AMW3677	2-Methylbutanoic anhydride	Organic acid and Its derivatives	C10H18O3	-
559	AMW4720	2-Hexanone, 6-methoxy-	Aldehyde, Ketones, Esters	C7H14O2	-
560	AMW2860	Succinimide	Alcohol and amines	C4H5NO2	amine, ammoniacal, piperidine
561	AMW0317	CyclohexylAmine	Alcohol and amines	C6H13N	-
562	AMW4472	Isobutyric acid, 2,2,2-trichloroethyl ester	Aldehyde, Ketones, Esters	C6H9Cl3O2	-
563	AMW3233	(1R,2S,6S,7S,8S)-8-Isopropyl-1-methyl-3-methylenetricyclo[4.4.0.02,7]decane-rel-	Terpenoids	C15H24	-
564	AMW0075	Fumaric acid, ethyl 2-methylallyl ester	Aldehyde, Ketones, Esters	C10H14O4	-
565	AMW1343	Eucalyptol	Terpenoids	C10H18O	eucalyptus, herbal, camphor, medicinal
566	AMW3959	Carbonic acid, hexyl methyl ester	Aldehyde, Ketones, Esters	C8H16O3	-

567	AMW2496	Heptane, 2,2,4,6,6-pentamethyl-	Hydrocarbons	C12H26	-
568	AMW0050	Ethyl 4-(ethyloxy)-2-oxobut-3-enoate	Aldehyde, Ketones, Esters	C8H12O4	-
569	AMW3860	2(5H)-Furanone, 5-(1-methylethyl)-	Aldehyde, Ketones, Esters	C7H10O2	-
570	AMW2786	3-Methylpyridazine	Heterocyclic compounds	C5H6N2	-
571	AMW0994	Cyclopentaneacetic acid, 3-oxo-2-pentyl-, methyl ester	Aldehyde, Ketones, Esters	C13H22O3	floral, oily, jasmin, green, lactonic
572	AMW4068	Creatinine	Organic acid and Its derivatives	C4H7N3O	-
573	AMW4461	Phenol, 2-amino-4-methoxy-	Benzene and substituted derivatives	C7H9NO2	-
574	AMW1829	Octane, 1,1'-oxybis-	Ethers	C16H34O	-
575	AMW0210	BenzAldehyde, 4-methyl-	Aldehyde, Ketones, Esters	C8H8O	fruity, cherry, deep, phenol
576	AMW1579	Benzenemethanol, .alpha.-2-cyclohexen-1-yl-	Alcohol and amines	C13H16O	-
577	AMW3172	Pentanamide, 2-amino-4-methyl-, (S)-	Alcohol and amines	C6H14N2O	-
578	AMW2656	2-Naphthalenemethanol, decahydro-.alpha.,.alpha.,4a-trimethyl-8-methylene-, [2R-(2.alpha.,4a.alpha.,8a.beta.)]-	Terpenoids	C15H26O	woody, green
579	AMW0134	Ethyl {[(ethylthio)carbonyl]thio}acetate	Aldehyde, Ketones, Esters	C7H12O3S2	-
580	AMW1518	2,4-dimethyl-1-Decene	Hydrocarbons	C12H24	-
581	AMW4473	Heptane, 3-[(ethenyloxy)methyl]-	Ethers	C10H20O	-
582	AMW3082	2-Piperazinone	Aldehyde, Ketones, Esters	C4H8N2O	-
583	AMW1896	3,5-Dimethylpyrazole	Heterocyclic compounds	C5H8N2	-
584	AMW3187	Hexanol-1, 6-cyclohexyl-	Alcohol and amines	C12H24O	-
585	AMW1823	Pentadecane	Hydrocarbons	C15H32	waxy
586	AMW3185	1-(4-Aminophenyl)ethanol	Alcohol and amines	C8H11NO	-
587	AMW2902	1H-1,2,3-Triazole-4-carboxaldehyde	Aldehyde, Ketones, Esters	C3H3N3O	-
588	AMW2517	exo-2-Hydroxycineole acetate	Aldehyde, Ketones, Esters	C12H20O3	-
589	AMW0049	2-Methyl-furan-3-carboxylic acid N'-acetyl-hydrazide	Heterocyclic compounds	C8H10N2O3	-
590	AMW3658	1H-Cyclopropa[a]naphthalene, 1a,2,3,5,6,7,7a,7b-octahydro-	Terpenoids	C15H24	-

		1,1,7,7a-tetramethyl-, [1aR-(1a.alpha.,7.alpha.,7a.alpha.,7b.alpha.)]-			
591	AMW0821	(Z)-6-dodecen-γ-lactone	Aldehyde, Ketones, Esters	C12H20O2	sweet, fatty, waxy, dairy, creamy, fruity
592	AMW0154	2,4,6-Pyrimidinetriamine	Alcohol and amines	C4H7N5	-
593	AMW3303	Decane, 2,3,5,8-tetramethyl-	Hydrocarbons	C14H30	-
594	AMW1978	(Methylsulfamoyl)amine	Alcohol and amines	CH6N2O2S	-
595	AMW0490	2-Imidazolidinone	Aldehyde, Ketones, Esters	C3H6N2O	-
596	AMW4946	Pentanedioic acid	Organic acid and Its derivatives	C5H8O4	-
597	AMW3192	Cyclobutanecarboxylic acid, 2-dimethylaminoethyl ester	Aldehyde, Ketones, Esters	C9H17NO2	-
598	AMW2120	(2,2,6-Trimethyl-bicyclo[4.1.0]hept-1-yl)-methanol	Alcohol and amines	C11H20O	-
599	AMW3547	Ethanediamide	Alcohol and amines	C2H4N2O2	-
600	AMW4208	1-Propanol, dl-2-benzylamino-,	Alcohol and amines	C10H15NO	-
601	AMW1462	3-ethyl-Pyridine	Heterocyclic compounds	C7H9N	tobacco, oakmoss, leathery
602	AMW0966	1-(1-methylethenyl)-4-(1-methylethyl)-Benzene	Benzene and substituted derivatives	C12H16	-
603	AMW3473	1,3,5-Triazine-2,4,6-triamine	Heterocyclic compounds	C3H6N6	-
604	AMW2741	Maleic anhydride	Organic acid and Its derivatives	C4H2O3	-
605	AMW2777	2,4-Dimethyldodecane	Hydrocarbons	C14H30	-
606	AMW1944	2-Methyl-1-nonene-3-yne	Hydrocarbons	C10H16	-
607	AMW2684	2,4,6,(1H,3H,5H)-Pyrimidinetrione, 5-acetyl-	Aldehyde, Ketones, Esters	C6H6N2O4	-
608	AMW4070	4,5-Diamino-6-hydroxypyrimidine	Heterocyclic compounds	C4H6N4O	-
609	AMW0819	N-(n-Butoxymethyl)acrylamide	Alcohol and amines	C8H15NO2	-
610	AMW3323	Dodecane, 1-methoxy-	Ethers	C13H28O	-
611	AMW3154	2,5-Dimethoxy-4-ethylamphetamine	Alcohol and amines	C13H21NO2	-
612	AMW3629	Ethanone, 1-(1-methylcyclohexyl)-	Aldehyde, Ketones, Esters	C9H16O	-
613	AMW3982	2H-Pyran-2-one, 3-acetyl-4-hydroxy-6-methyl-	Organic acid and Its derivatives	C8H8O4	-

614	AMW4438	Ethanol, 2,2'-oxybis-, dipropanoate	Aldehyde, Ketones, Esters	C10H18O5	-
615	AMW2050	1-chloro-2,2-dimethyl-Propane	Halogenated hydrocarbons	C5H11Cl	-
616	AMW3403	1H-1,2,3,4-Tetrazole, 5-hydrazino-	Nitrogen compounds	CH4N6	-
617	AMW0066	Oxalic acid, hexyl neopentyl ester	Aldehyde, Ketones, Esters	C13H24O4	-
618	AMW3214	4-Heptanone, 2,2,3,3,5,5,6,6-octamethyl-	Aldehyde, Ketones, Esters	C15H30O	-
619	AMW2909	2-p-Tolylpyridine	Heterocyclic compounds	C12H11N	-
620	AMW2389	Propyl octanoate	Aldehyde, Ketones, Esters	C11H22O2	coconut
621	AMW4968	Benzeneacetic acid, .alpha.-methyl-	Organic acid and Its derivatives	C9H10O2	-
622	AMW3247	Phenol, 4-pentyl-	Benzene and substituted derivatives	C11H16O	-
623	AMW2862	Terpinyl formate	Aldehyde, Ketones, Esters	C11H18O2	-
624	AMW0856	1,14-Tetradecanediol	Alcohol and amines	C14H30O2	-
625	AMW0103	Ethyl 2-(5-methyl-5-vinyltetrahydrofuran-2-yl)propan-2-yl carbonate	Aldehyde, Ketones, Esters	C13H22O4	-
626	AMW3747	5-Pyrimidinol, 2-methyl-	Alcohol and amines	C5H6N2O	-
627	AMW4976	6-Dodecanone	Aldehyde, Ketones, Esters	C12H24O	-
628	AMW3694	2-Hexyl-5-pentylpyrrolidine	Heterocyclic compounds	C15H31N	-
629	AMW4221	Acrylanilide	Alcohol and amines	C9H9NO	-
630	AMW3803	Hexyl ethylphosphonofluoridate	Others	C8H18FO2 P	-
631	AMW5072	3-Hydroxy-4-methoxybenzoic acid	Organic acid and Its derivatives	C8H8O4	-
632	AMW1300	1H-Tetrazol-5-amine	Alcohol and amines	CH3N5	-
633	AMW3641	Diethylene glycol dibutyrate	Aldehyde, Ketones, Esters	C12H22O5	-
634	AMW3721	1,2,4-Triazine-5-ol, 3,6-dimethyl-	Alcohol and amines	C5H7N3O	-
635	AMW0451	4-[pyrrolidin-2-one-5-yl]-Butan-2-one	Aldehyde, Ketones, Esters	C8H13NO2	-
636	AMW3439	Hydratropic acid, isopropyl ester	Aldehyde, Ketones, Esters	C12H16O2	-
637	AMW2413	Tridecane, 5-methyl-	Hydrocarbons	C14H30	-

638	AMW1118	Ethanone, 1-(2-methyl-1-cyclopenten-1-yl)-	Aldehyde, Ketones, Esters	C8H12O	-
639	AMW0476	Benzophenone	Aldehyde, Ketones, Esters	C13H10O	balsamic, rose, metallic, powdery, geranium
640	AMW0038	Methyl trans-2-(3-cyclopropyl-7-norcaranyl)acetate	Aldehyde, Ketones, Esters	C13H20O2	-
641	AMW5037	3-Hydroxynonanoic acid	Organic acid and Its derivatives	C9H18O3	-
642	AMW0783	1H-Imidazole-2-methanol, 1-methyl-	Alcohol and amines	C5H8N2O	-
643	AMW1412	Sarcosine anhydride	Aldehyde, Ketones, Esters	C6H10N2O2	-
644	AMW1201	1-Ethyl-4,4-dimethyl-2,5-dioxoimidazolidine	Heterocyclic compounds	C7H12N2O2	-
645	AMW1273	2-methoxy-6-methyl-4H-Pyran-4-one	Aldehyde, Ketones, Esters	C7H8O3	-
646	AMW2765	2-Methyl-1-tetradecene	Hydrocarbons	C15H30	-
647	AMW2788	1,2,4,5-Tetrazine-3,6-diamine	Alcohol and amines	C2H4N6	-
648	AMW2883	Decane, 2,3,5-trimethyl-	Hydrocarbons	C13H28	-
649	AMW3532	1-Propoxy-3,3-diethyltriazene 2-oxide	Nitrogen compounds	C7H17N3O2	-
650	AMW5045	9-Oxononanoic acid	Organic acid and Its derivatives	C9H16O3	-
651	AMW4819	Fenchol	Terpenoids	C10H18O	camphor, borneol, pine, woody, dry, sweet, lemon
652	AMW4836	.alpha.-Santoline alcohol	Alcohol and amines	C10H18O	-
653	AMW4105	2',4'-Dihydroxy-3'-methylacetophenone	Aldehyde, Ketones, Esters	C9H10O3	-
654	AMW5108	8-Pentadecanone	Aldehyde, Ketones, Esters	C15H30O	-
655	AMW2498	4-Terpinenyl acetate	Aldehyde, Ketones, Esters	C12H20O2	-
656	AMW0252	2-Propenoic acid, oxiranylmethyl ester	Aldehyde, Ketones, Esters	C6H8O3	-
657	AMW2084	Vinyl lauryl ether	Ethers	C14H28O	-
658	AMW3020	2-Isopropenyl-4a,8-dimethyl-1,2,3,4,4a,5,6,8a-octahydronaphthalene	Terpenoids	C15H24	-
659	AMW3039	3,6-Dimethylpiperazine-2,5-dione	Aldehyde, Ketones, Esters	C6H10N2O	-

				2	
660	AMW2790	Furfurylmethylamphetamine	Alcohol and amines	C15H19NO	-
661	AMW0406	N,N-dimethyl-1-Dodecanamine	Alcohol and amines	C14H31N	-
662	AMW3755	dl-2-Aminobutyric acid	Organic acid and Its derivatives	C4H9NO2	-
663	AMW2334	Pentanoic acid, 2-ethylhexyl ester	Aldehyde, Ketones, Esters	C13H26O2	-
664	AMW3636	1-(3-Methyl-2-butenoxy)-4-(1-propenyl)benzene	Benzene and substituted derivatives	C14H18O	-
665	AMW1891	2,4,6-Octatriene, 2,6-dimethyl-	Terpenoids	C10H16	sweet, floral, nut skin, peppery, herbal, tropical
666	AMW0671	N,N-dibutyl-Acetamide	Alcohol and amines	C10H21NO	-
667	AMW3579	Ethanone, 1-(2,3,4-trimethylphenyl)-	Aldehyde, Ketones, Esters	C11H14O	-
668	AMW0053	Sulfurous acid, butyl pentyl ester	Aldehyde, Ketones, Esters	C9H20O3S	-
669	AMW2571	Tetradecane, 5-methyl-	Hydrocarbons	C15H32	-
670	AMW3511	Lacthydrazide	Nitrogen compounds	C3H8N2O2	-
671	AMW1292	9-Azabicyclo[3.3.1]nonan-3-one	Aldehyde, Ketones, Esters	C8H13NO	-
672	AMW3059	2-Propenoic acid, 2-methyl-, propyl ester	Aldehyde, Ketones, Esters	C7H12O2	-
673	AMW5116	2(3H)-Benzothiazolone	Aldehyde, Ketones, Esters	C7H5NOS	-
674	AMW2027	(E)-2-Tridecen-1-ol	Hydrocarbons	C13H26O	mild, waxy, clean, watery, cilantro, rue, citrus peel, laundered cloth, fatty
675	AMW0449	1,3-Cyclohexadiene-1-carboxaldehyde, 2,6,6-trimethyl-	Terpenoids	C10H14O	fresh, herbal, phenol, metallic, rosemary, tobacco, spicy
676	AMW0619	1-Nonanol	Alcohol and amines	C9H20O	fresh, clean, fatty, floral, rose, orange, dusty, wet, oily
677	AMW3396	Acetamide, N-(aminocarbonyl)-	Alcohol and amines	C3H6N2O2	-
678	AMW4017	Butane, 2,3-dimethyl-2,3-dinitro-	Nitrogen compounds	C6H12N2O4	-
679	AMW0559	2-Bromo dodecane	Halogenated hydrocarbons	C12H25Br	-
680	AMW1230	Pentadecane, 2,6,10-trimethyl-	Hydrocarbons	C18H38	-
681	AMW3440	l-Alanine, N-(2-furoyl)-, ethyl ester	Aldehyde, Ketones, Esters	C10H13NO4	-
682	AMW3812	Acetoxyacetic acid, 4-cyanophenyl ester	Aldehyde, Ketones, Esters	C11H9NO4	-

683	AMW4952	2-Decanone, 5,9-dimethyl-	Aldehyde, Ketones, Esters	C12H24O	-
684	AMW3898	Thiophene-2-acetic acid, 2-dimethylaminoethyl ester	Aldehyde, Ketones, Esters	C10H15NO2S	-
685	AMW4777	Pyrazine, (2-methylpropyl)-	Heterocyclic compounds	C8H12N2	Green, pepper, coffee, roasted
686	AMW0493	(2S,4aS,5S,8aR)-2,5-diallyldecahydroquinoline	Heterocyclic compounds	C15H25N	-
687	AMW0912	2,6-Dodecadien-1-al	Aldehyde, Ketones, Esters	C12H20O	citrus, mandarin, orange, melon
688	AMW2925	1-Decanol, 2-ethyl-	Alcohol and amines	C12H26O	-
689	AMW4182	2-Acetyl-7-hydroxybenzofuran	Heterocyclic compounds	C10H8O3	-
690	AMW2734	4H-Pyran-4-one, 2,6-dimethyl-	Aldehyde, Ketones, Esters	C7H8O2	-
691	AMW3149	Diethanolamine	Alcohol and amines	C4H11NO2	mild, rotten, fishy, ammoniacal
692	AMW4062	1H-Pyrrole-2,5-dione	Aldehyde, Ketones, Esters	C4H3NO2	-
693	AMW2444	Dimethyl phthalate	Aldehyde, Ketones, Esters	C10H10O4	-
694	AMW3406	Isobutyl 2-(4-methylcyclohex-3-enyl)propan-2-yl carbonate	Aldehyde, Ketones, Esters	C15H26O3	-
695	AMW3489	N-Methyl-L-prolinol	Alcohol and amines	C6H13NO	-
696	AMW2588	Pentadecane, 2-methyl-	Hydrocarbons	C16H34	-
697	AMW3773	3-Isopropyl-6,10-dimethylundecane-2-ol	Alcohol and amines	C16H34O	-
698	AMW0425	Dodecanal	Aldehyde, Ketones, Esters	C12H24O	soapy, waxy, aldehydic, citrus, green, floral
699	AMW3746	Sulfurous acid, isobutyl pentyl ester	Aldehyde, Ketones, Esters	C9H20O3S	-
700	AMW2272	5-Ethyl-5-(propan-2-yl)imidazolidine-2,4-dione	Aldehyde, Ketones, Esters	C8H14N2O2	-
701	AMW2315	Heptylcyclohexane	Hydrocarbons	C13H26	-
702	AMW2379	4-Methylpentyl 2-methylbutanoate	Aldehyde, Ketones, Esters	C11H22O2	-
703	AMW4345	Dichloroacetic acid, 2-tetrahydrofurylmethyl ester	Aldehyde, Ketones, Esters	C7H10Cl2O3	-
704	AMW0013	6Z-2,5,5,10-Tetramethyl-undeca-2,6,9-trien-8-one	Terpenoids	C15H24O	-
705	AMW2781	1-Pentanone, 1-(4-methylphenyl)-	Aldehyde, Ketones, Esters	C12H16O	-

706	AMW2312	2-Acetylthiazole	Heterocyclic compounds	C5H5NOS	nutty, popcorn, roasted, peanut, hazelnut
707	AMW2844	Oxalic acid, allyl isobutyl ester	Aldehyde, Ketones, Esters	C9H14O4	-
708	AMW4504	1,5-Dimethyl-2-pyrrolecarbonitrile	Heterocyclic compounds	C7H8N2	-
709	AMW2443	1H-Inden-5-ol, 2,3-dihydro-	Benzene and substituted derivatives	C9H10O	-
710	AMW2920	Pentadecane, 7-methyl-	Hydrocarbons	C16H34	-
711	AMW0055	Sulfurous acid, 2-ethylhexyl isobutyl ester	Organic acid and Its derivatives	C12H26O3S	-
712	AMW3047	Pentadecane, 6-methyl-	Hydrocarbons	C16H34	-
713	AMW5069	2,2-Dimethylpropionic acid, decyl ester	Aldehyde, Ketones, Esters	C15H30O2	-
714	AMW0168	4-Methoxyphenylaldehyde trimethylene acetal	Aldehyde, Ketones, Esters	C11H14O3	-
715	AMW3723	4-Pyridinamine, N,N,2,6-tetramethyl-	Alcohol and amines	C9H14N2	-
716	AMW2857	Hexadecane, 7-methyl-	Hydrocarbons	C17H36	-
717	AMW2546	(1R,3aS,5aS,8aR)-1,3a,4,5a-Tetramethyl-1,2,3,3a,5a,6,7,8-octahydrocyclopenta[c]pentalene	Terpenoids	C15H24	-
718	AMW1556	1-Dodecen-1-ol, acetate	Aldehyde, Ketones, Esters	C14H26O2	-
719	AMW3938	Heptyl ethylphosphonofluoridate	Aldehyde, Ketones, Esters	C9H20FO2P	-
720	AMW3610	1-Undecanol, acetate	Aldehyde, Ketones, Esters	C13H26O2	-
721	AMW0145	3-methyl-Undecane	Hydrocarbons	C12H26	-
722	AMW2941	Hexadecane, 7,9-dimethyl-	Hydrocarbons	C18H38	-
723	AMW1045	tetramethyl-Thiourea	Nitrogen compounds	C5H12N2S	-
724	AMW3870	2,4,5-Trioxoimidazolidine	Heterocyclic compounds	C3H2N2O3	-
725	AMW1140	.delta.-Nonalactone	Aldehyde, Ketones, Esters	C9H16O2	coconut, creamy, sweet, milky, coumarin
726	AMW4046	5-Cyano-1,2,3,4-tetrahydro-4,6-dimethyl-2-oxopyridine	Heterocyclic compounds	C8H10N2O	-
727	AMW1822	Tetradecane	Hydrocarbons	C14H30	mild, waxy
728	AMW4099	Undecanoic acid, ethyl ester	Aldehyde, Ketones, Esters	C13H26O2	soapy, waxy, fatty, cognac, coconut
729	AMW2098	Cedrol	Terpenoids	C15H26O	cedarwood, woody, dry, sweet

730	AMW4984	Benzoic acid, 4-methoxy-, methyl ester	Aldehyde, Ketones, Esters	C9H10O3	herbal, anisic, sweet
731	AMW0488	Indole	Heterocyclic compounds	C8H7N	animalic, floral, moth, mothball, fecal, naphthelene
732	AMW0399	1-Tetradecene	Hydrocarbons	C14H28	mild pleasant
733	AMW4161	2,6-Octadien-1-amine, 3,7-dimethyl-	Alcohol and amines	C10H19N	-
734	AMW0394	Ethanol, 2-(2-ethoxyethoxy)-	Alcohol and amines	C6H14O3	slightly, ethereal
735	AMW1053	Pentadecane, 4-methyl-	Hydrocarbons	C16H34	-
736	AMW2820	N,N'-Trimethyleneurea	Nitrogen compounds	C4H8N2O	-
737	AMW2401	Hydrazine, (phenylmethyl)-	Nitrogen compounds	C7H10N2	-
738	AMW1232	1-Methylimidazole-5-carboxaldehyde	Aldehyde, Ketones, Esters	C5H6N2O	-
739	AMW2769	Ethyl tridecanoate	Aldehyde, Ketones, Esters	C15H30O2	-
740	AMW0511	Nonanoic acid, ethyl ester	Aldehyde, Ketones, Esters	C11H22O2	fruity, rose, waxy, rummy, wine, natural, tropical
741	AMW2421	Phenol, 2,4,6-tris(1-methylethyl)-	Benzene and substituted derivatives	C15H24O	-
742	AMW3330	2-Amino-4-methyl-oxazole	Heterocyclic compounds	C4H6N2O	-
743	AMW2374	Vanillin	Aldehyde, Ketones, Esters	C8H8O3	sweet, vanilla, creamy, chocolate
744	AMW4947	1-Tridecene	Hydrocarbons	C13H26	-
745	AMW1604	3-methyl-6-(1-methylethylidene)-Cyclohexene	Terpenoids	C10H16	-
746	AMW1248	Cyclohexanol, 3-(3,3-dimethylbutyl)-	Hydrocarbons	C12H24O	-
747	AMW3119	4,6-Octadiyn-3-one, 2-methyl-	Aldehyde, Ketones, Esters	C9H10O	-
748	AMW4213	3,4,5-Trihydroxyphthalaldehyde	Aldehyde, Ketones, Esters	C8H6O5	-
749	AMW3266	1,2-Ethanediol, monobenzoate	Aldehyde, Ketones, Esters	C9H10O3	-
750	AMW3765	3(2H)-Furanone, 2-(1-hydroxy-1-methyl-2-oxopropyl)-2,5-dimethyl-	Aldehyde, Ketones, Esters	C10H14O4	-
751	AMW0814	Pyrazine, 2,5-dimethyl-3-(3-methylbutyl)-	Heterocyclic compounds	C11H18N2	fruity
752	AMW4995	Taurine	Organic acid and Its derivatives	C2H7NO3S	-
753	AMW4996	4-Piperidinecarboxamide	Alcohol and amines	C6H12N2O	-
754	AMW4953	Butanedioic acid, methylene-	Organic acid and Its derivatives	C5H6O4	-

755	AMW0670	Hexadecane, 2-methyl-	Hydrocarbons	C17H36	-
756	AMW2418	1-Methyl-4-(6-methylhept-5-en-2-yl)cyclohexa-1,3-diene	Terpenoids	C15H24	-
757	AMW0537	Tetradecanal	Aldehyde, Ketones, Esters	C14H28O	fatty, waxy, amber, incense, dry, citrus, peel, musky
758	AMW0794	1-(2-methyl-2-cyclopenten-1-yl)-Ethanone	Aldehyde, Ketones, Esters	C8H12O	-
759	AMW3720	1,1,2,2-Tetrakis(allyloxy)ethane	Ethers	C14H22O4	-
760	AMW2778	4-tert-Butyl-2-nitrophenol, acetate	Aldehyde, Ketones, Esters	C12H15NO4	-
761	AMW3574	Oxalic acid, cyclobutyl hexyl ester	Aldehyde, Ketones, Esters	C12H20O4	-
762	AMW4307	4(1H)-Pyrimidinone, 2-methyl-	Aldehyde, Ketones, Esters	C5H6N2O	-
763	AMW1485	2,6,10,10-tetramethyl-1-Oxaspiro[4.5]deca-3,6-diene	Heterocyclic compounds	C13H20O	-
764	AMW5115	Benzene, decyl-	Benzene and substituted derivatives	C16H26	-
765	AMW3008	Propanedioic acid	Organic acid and Its derivatives	C3H4O4	-
766	AMW5018	Isosativene	Hydrocarbons	C15H24	-
767	AMW5089	1-Iodo-2-methylundecane	Halogenated hydrocarbons	C12H25I	-
768	AMW4463	2-Pyrazoline, 1-methyl-4-propyl-	Heterocyclic compounds	C7H14N2	-
769	AMW4442	Cyclohexanecarboxylic acid, 1-amino-	Organic acid and Its derivatives	C7H13NO2	-
770	AMW1088	Cyclotetradecane	Hydrocarbons	C14H28	-
771	AMW2363	6-Octenal, 7-methyl-3-methylene-	Terpenoids	C10H16O	juicy, aldehydic, lemon grass, floral, lemon, bois, rose
772	AMW0438	methyl-Carbamic acid,3-methylphenyl ester	Aldehyde, Ketones, Esters	C9H11NO2	-
773	AMW3986	N-[3-[N-Aziridyl]propylidene]tetrahydrofurfurylamine	Heterocyclic compounds	C10H18N2O	-
774	AMW4376	2-Methoxybenzyl alcohol, 2-methylbutyl ether	Ethers	C13H20O2	-
775	AMW3756	2,5-Piperazinedione, 3-methyl-	Aldehyde, Ketones, Esters	C5H8N2O2	-
776	AMW1294	4-(phenylmethoxy)-Benzaldehyde	Aldehyde, Ketones, Esters	C14H12O2	-
777	AMW3284	2-Cyanoguanidine	Nitrogen compounds	C2H4N4	-
778	AMW2099	Propanoic acid, 2-methyl-, 3-hydroxy-2,2,4-trimethylpentyl	Aldehyde, Ketones, Esters	C12H24O3	-

		ester			
779	AMW0487	(E)-Hex-3-enyl (E)-2-methylbut-2-enoate	Aldehyde, Ketones, Esters	$C_{11}H_{18}O_2$	-
780	AMW4956	Heptane, 2-(hexyloxy)-	Ethers	$C_{13}H_{28}O$	-
781	AMW4784	Benzene, (1-chloroethyl)-	Benzene and substituted derivatives	C_8H_9Cl	-
782	AMW4337	4-Amino-3,5-dimethyl-1,2,4-triazole	Heterocyclic compounds	$C_4H_8N_4$	-
783	AMW0915	Diallyl disulphide	Ethers	$C_6H_{10}S_2$	alliaceous, onion, garlic, metallic
784	AMW0231	Geranyl acetate	Aldehyde, Ketones, Esters	$C_{12}H_{20}O_2$	lemon
785	AMW0582	6,7-Dodecanedione	Aldehyde, Ketones, Esters	$C_{12}H_{22}O_2$	-
786	AMW4334	Oxalic acid, cyclohexyl ethyl ester	Aldehyde, Ketones, Esters	$C_{10}H_{16}O_4$	-
787	AMW2873	3,5-Diamino-1,2,4-triazole	Heterocyclic compounds	$C_2H_5N_5$	-
788	AMW0133	1-Ethyl-1H-pyrazole-3,4-diamine	Alcohol and amines	$C_5H_{10}N_4$	-
789	AMW1321	(1-pentylhexyl)-Benzene	Benzene and substituted derivatives	$C_{17}H_{28}$	-
790	AMW3174	1-Propanone, 2,2-dimethyl-1-(4-methylphenyl)-3-(1-piperidyl)-	Aldehyde, Ketones, Esters	$C_{17}H_{25}NO$	-
791	AMW0839	Pentadecane, 2,6,10,14-tetramethyl-	Hydrocarbons	$C_{19}H_{40}$	-
792	AMW1881	Benzoic acid	Organic acid and Its derivatives	$C_7H_6O_2$	faint, balsamic, urine
793	AMW5172	Hexadecane, 1-bromo-	Halogenated hydrocarbons	$C_{16}H_{33}Br$	-
794	AMW4012	2-Isopropenyl-3-methylpyrazine	Heterocyclic compounds	$C_8H_{10}N_2$	-
795	AMW2329	Butanedioic acid, dimethyl ester	Aldehyde, Ketones, Esters	$C_6H_{10}O_4$	estery, fruity, green, floral, rummy, tutti-frutti, bubble, gum, papaya
796	AMW2434	Propanoic acid, 2-methyl-, 2-phenylethyl ester	Aldehyde, Ketones, Esters	$C_{12}H_{16}O_2$	floral, fruity, rose, tea, rose, peach, pastry
797	AMW3799	2-(Diethylaminophenylmethyl)cyclohexanol	Alcohol and amines	$C_{17}H_{27}NO$	-
798	AMW1028	Tetradecanoic acid, 10,13-dimethyl-, methyl ester	Aldehyde, Ketones, Esters	$C_{17}H_{34}O_2$	-
799	AMW0600	2,6-Octadien-1-ol, 3,7-dimethyl-, acetate, (Z)-	Aldehyde, Ketones, Esters	$C_{12}H_{20}O_2$	floral, rose, soapy, citrus, dewy, pear
800	AMW1338	Cyclohexanone, 2-isobutyl-	Aldehyde, Ketones, Esters	$C_{10}H_{18}O$	-
801	AMW3995	Thiophene-3-acetic acid hydrazide	Nitrogen compounds	$C_6H_8N_2OS$	-
802	AMW0719	1-(1-cyclohexen-1-yl)-1-Propanone	Aldehyde, Ketones, Esters	$C_9H_{14}O$	-

803	AMW0599	3,5,9-Undecatrien-2-one, 6,10-dimethyl-	Aldehyde, Ketones, Esters	C13H20O	sweet, waxy, citrus, floral, balsamic, dry, dusty, powdery, spicy
804	AMW0104	(4-Methylphenyl) methanol, neopentyl ether	Ethers	C13H20O	-
805	AMW1004	Tetradecane, 4-methyl-	Hydrocarbons	C15H32	-
806	AMW5107	Methyl dihydrojasmonate,cis-	Aldehyde, Ketones, Esters	C13H22O3	jasmine
807	AMW4166	Isobutyl (5-isopropyl-2-methylphenyl) carbonate	Aldehyde, Ketones, Esters	C15H22O3	-
808	AMW5081	1,6-Dioxacyclododecane-7,12-dione	Aldehyde, Ketones, Esters	C10H16O4	-
809	AMW4498	1,11-Undecanediol	Alcohol and amines	C11H24O2	-
810	AMW3318	Oxalic acid, hexyl 2-phenylethyl ester	Aldehyde, Ketones, Esters	C16H22O4	-
811	AMW1095	N,N-dimethyl-1-Heptadecanamine	Alcohol and amines	C19H41N	-
812	AMW5017	2-Butanone, 4-(2,6,6-trimethyl-1,3-cyclohexadien-1-yl)-	Aldehyde, Ketones, Esters	C13H20O	floral
813	AMW1224	Copaene	Terpenoids	C15H24	woody, spicy, honey
814	AMW0415	Hexadecanoic acid, methyl ester	Aldehyde, Ketones, Esters	C17H34O2	oily, waxy, fatty, orris
815	AMW4815	Ethanol, 2-(hexyloxy)-	Alcohol and amines	C8H18O2	-
816	AMW4340	Octanoic acid, 2-tetrahydrofurylmethyl ester	Aldehyde, Ketones, Esters	C13H24O3	-
817	AMW3431	(3R,4aS,8aS)-8a-Methyl-5-methylene-3-(prop-1-en-2-yl)-1,2,3,4,4a,5,6,8a-octahydronaphthalene	Terpenoids	C15H22	-
818	AMW2229	Phenol, 2-methoxy-4-(1-propenyl)-, acetate	Aldehyde, Ketones, Esters	C12H14O3	sweet, spicy, powdery, floral, carnation, balsamic
819	AMW0249	Tris-(hydroxymethyl)-phosphine oxide	Others	C3H9O4P	-
820	AMW0423	1-chloro-Dodecane	Halogenated hydrocarbons	C12H25Cl	-
821	AMW2394	Tricyclo[2.2.1.0(2,6)]heptane, 1,7-dimethyl-7-(4-methyl-3-pentenyl)-, (-)-	Hydrocarbons	C15H24	sweet, woody
822	AMW1519	Ethanone, 1-(2-aminophenyl)-	Aldehyde, Ketones, Esters	C8H9NO	grape, sweet
823	AMW3742	N-(N-Allylformamide)ethyleneimine	Alcohol and amines	C6H10N2O	-
824	AMW4209	4-Pyridinol, 3-methoxy-2-methyl-	Alcohol and amines	C7H9NO2	-
825	AMW1375	2,4,5-Trihydroxypyrimidine	Heterocyclic compounds	C4H4N2O3	-
826	AMW4216	Cyclohexane, 1,1'-(oxydi-2,1-ethanediyl)bis[4-methyl-	Ethers	C18H34O	-

827	AMW2774	1,2,4,5-Tetrazine, 3,6-dimethyl-	Heterocyclic compounds	C4H6N4	-
828	AMW2798	2-Furancarboxylic acid, tetrahydro-3-methyl-5-oxo-	Organic acid and Its derivatives	C6H8O4	-
829	AMW4961	Bicyclo[2.2.1]heptane-2,3-dione, 1,7,7-trimethyl-, (1S)-	Aldehyde, Ketones, Esters	C10H14O2	-
830	AMW1090	Cyclohexadecane	Hydrocarbons	C16H32	-
831	AMW0061	Oxalic acid, 6-ethyloct-3-yl propyl ester	Aldehyde, Ketones, Esters	C15H28O4	-
832	AMW4607	3-Hexanol, 2-methyl-	Alcohol and amines	C7H16O	-
833	AMW3880	Furo[2,3-b]pyridine, 2-methyl-	Heterocyclic compounds	C8H7NO	-
834	AMW4197	3-Buten-2-one, 4-(2,2-dimethyl-1-aziridinyl)-	Aldehyde, Ketones, Esters	C8H13NO	-
835	AMW1935	3,5-Octadien-2-ol	Alcohol and amines	C8H14O	-
836	AMW2867	Heptane, 2,5,5-trimethyl-	Hydrocarbons	C10H22	-
837	AMW3189	Ethyl mandelate	Aldehyde, Ketones, Esters	C10H12O3	-
838	AMW5027	.beta.-Humulene	Terpenoids	C15H24	-
839	AMW5173	8-Octadecanone	Aldehyde, Ketones, Esters	C18H36O	-
840	AMW1402	4-acetyl-2,3,4,5,5-pentamethyl-2-Cyclopenten-1-one	Aldehyde, Ketones, Esters	C12H18O2	-
841	AMW2377	Diethyl malonate	Aldehyde, Ketones, Esters	C7H12O4	sweet, fruity, green, apple
842	AMW4367	4-Acetoxy-3-methoxystyrene	Aldehyde, Ketones, Esters	C11H12O3	-
843	AMW2351	Ethanone, 1-(2,4-dihydroxyphenyl)-	Aldehyde, Ketones, Esters	C8H8O3	-
844	AMW1716	2,4-Imidazolidinedione, 1-methyl-	Nitrogen compounds	C4H6N2O2	-
845	AMW4411	(-)-delta.-Panasinsine	Terpenoids	C15H24	-
846	AMW2463	2,3,3,4,7-Pentamethyl-2,3-dihydro-benzofuran	Heterocyclic compounds	C13H18O	-
847	AMW1237	2-Furancarboxylic acid, hexyl ester	Aldehyde, Ketones, Esters	C11H16O3	floral, waxy, green, sweet, pear, apple
848	AMW1290	4-Quinolinecarboxaldehyde	Aldehyde, Ketones, Esters	C10H7NO	-
849	AMW3800	Myrtene acid bromide	Halogenated hydrocarbons	C10H13BrO	-
850	AMW1689	6,8-Nonadien-2-one, 6-methyl-5-(1-methylethylidene)-	Aldehyde, Ketones, Esters	C13H20O	-
851	AMW3524	3,4-Diamino-1,2,4(4H)-triazole	Heterocyclic compounds	C2H5N5	-
852	AMW0097	5-Butyl-5-ethylheptadecane	Hydrocarbons	C23H48	-

853	AMW4750	1,2-Cyclopentanedione, 3-methyl-	Aldehyde, Ketones, Esters	C6H8O2	Caramellic
854	AMW4660	Acetohydroxamic acid	Alcohol and amines	C2H5NO2	-
855	AMW3181	Hexadecane, 5-butyl-	Hydrocarbons	C20H42	-
856	AMW5059	1,3-Benzodioxole, 4-methoxy-6-(2-propenyl)-	Ethers	C11H12O3	spicy warm
857	AMW0881	1-iodo-Decane	Halogenated hydrocarbons	C10H21I	-
858	AMW3819	N-Ethyl-2-isopropoxycarbonylazetidine	Heterocyclic compounds	C9H17NO2	-
859	AMW1827	Heptadecane	Hydrocarbons	C17H36	alkane
860	AMW2179	1,3-dimethyl-2,4(1H,3H)-Pyrimidinedione	Aldehyde, Ketones, Esters	C6H8N2O2	-
861	AMW4722	2-Aminopyridine	Heterocyclic compounds	C5H6N2	-
862	AMW0745	3-Chloro-2,4-pentanedione	Halogenated hydrocarbons	C5H7ClO2	-
863	AMW1963	.delta.-Dodecalactone	Aldehyde, Ketones, Esters	C12H22O2	peachy, oily, creamy, soapy
864	AMW1575	n-Hexadecanoic Acid	Organic acid and Its derivatives	C16H32O2	waxy, fatty
865	AMW3991	2',4',5'-Trimethoxyacetophenone	Aldehyde, Ketones, Esters	C11H14O4	-
866	AMW0320	Cyclohexanone	Aldehyde, Ketones, Esters	C6H10O	minty, acetone
867	AMW3805	Cyclopropanecarboxylic acid, 4-nitrophenyl ester	Aldehyde, Ketones, Esters	C10H9NO4	-
868	AMW5085	1-Pentadecene, 2-methyl-	Hydrocarbons	C16H32	-
869	AMW1043	Pentadecanal	Aldehyde, Ketones, Esters	C15H30O	fresh, waxy
870	AMW3217	4-(Ethylamino)-6-hydroxypyrimidine	Heterocyclic compounds	C6H9N3O	-
871	AMW4075	1,7-Dimethyl-4-(1-methylethyl)cyclodecane	Hydrocarbons	C15H30	-
872	AMW3381	1,3-Dimethylimidazolidine-2,4-dione	Aldehyde, Ketones, Esters	C5H8N2O2	-
873	AMW0054	Sulfurous acid, butyl octyl ester	Aldehyde, Ketones, Esters	C12H26O3S	-
874	AMW4353	Fumaric acid, ethyl tetrahydrofurfuryl ester	Aldehyde, Ketones, Esters	C11H16O5	-
875	AMW4305	N-Butyryl-L-homoserine lactone	Aldehyde, Ketones, Esters	C8H13NO3	-
876	AMW2197	2-methoxy-Phenol	Benzene and substituted derivatives	C7H8O2	nutty
877	AMW3372	1-Dodecanol, 2-hexyl-	Alcohol and amines	C18H38O	-
878	AMW0069	d-Proline, N-methoxycarbonyl-, pentyl ester	Aldehyde, Ketones, Esters	C12H21NO	-

				4	
879	AMW0739	Di(n-hexyl)sulfone	Others	C12H26O2S	-
880	AMW2138	.alpha.-Terpinyl acetate	Aldehyde, Ketones, Esters	C12H20O2	herbal, bergamot, lavender, lime, citrus
881	AMW3038	2-Pyridinecarboxylic acid	Organic acid and Its derivatives	C6H5NO2	-
882	AMW3711	5H-Tetrazole-5-thione, 1-[2-(dimethylamino)ethyl]-1,2-dihydro-	Alcohol and amines	C5H11N5S	-
883	AMW5054	Benzenepropanal, 3-(1,1-dimethylethyl)-.alpha.-methyl-	Aldehyde, Ketones, Esters	C14H20O	-
884	AMW5168	(E,E)-7,11,15-Trimethyl-3-methylene-hexadeca-1,6,10,14-tetraene	Terpenoids	C20H32	-
885	AMW1815	2-Nonanol	Alcohol and amines	C9H20O	rose
886	AMW1869	1-[4-(1-methylethyl)phenyl]-Ethanone	Aldehyde, Ketones, Esters	C11H14O	spicy, woody, herbal, orris
887	AMW2723	l-Leucine, n-propargyloxycarbonyl-, isobutyl ester	Aldehyde, Ketones, Esters	C14H23NO4	-
888	AMW5154	1,2-Benzenedicarboxylic acid, bis(2-methylpropyl) ester	Aldehyde, Ketones, Esters	C16H22O4	-
889	AMW3954	Benzeneethanamine	Alcohol and amines	C8H11N	ammoniacal, fishy
890	AMW2635	1,5-Cyclooctadiene, 3,4-dimethyl-	Hydrocarbons	C10H16	-
891	AMW1495	.alpha.-methyl-Benzenepropanal	Aldehyde, Ketones, Esters	C10H12O	-
892	AMW5000	Phenol, 4-(1,1-dimethylpropyl)-	Benzene and substituted derivatives	C11H16O	-
893	AMW1142	Benzoic acid, 4-methyl, 3-methylbutyl ester	Aldehyde, Ketones, Esters	C13H18O2	-
894	AMW3196	Azulene, 1,4-dimethyl-7-(1-methylethyl)-	Terpenoids	C15H18	-
895	AMW3786	4a(2H)-Naphthalenemethanol, octahydro-	Alcohol and amines	C11H20O	-
896	AMW0502	BenzeneacetAldehyde	Aldehyde, Ketones, Esters	C8H8O	floral, honey, rose, cherry
897	AMW2948	Tetradecane, 4-ethyl-	Hydrocarbons	C16H34	-
898	AMW1657	2-Tridecanone	Aldehyde, Ketones, Esters	C13H26O	fatty, waxy, dairy, milky, coconut, nutty, herbal, earthy
899	AMW5061	.beta.-iso-Methyl ionone	Terpenoids	C14H22O	floral
900	AMW0193	Formamide, N-phenyl-	Alcohol and amines	C7H7NO	-

901	AMW2708	Phenol, 3,5-diethyl-	Benzene and substituted derivatives	C10H14O	-
902	AMW3528	Phthalic acid, cyclobutyl ethyl ester	Aldehyde, Ketones, Esters	C14H16O4	-
903	AMW4185	3,3,5,5-Tetramethylcyclohexanol	Alcohol and amines	C10H20O	-
904	AMW3314	Sulfurous acid, 2-ethylhexyl pentyl ester	Aldehyde, Ketones, Esters	C13H28O3S	-
905	AMW4441	5,11-Diethyl-8-methyl-7,9-dioxapentadecane	Hydrocarbons	C18H38O2	-
906	AMW2716	Hexadecane, 2,6,10-trimethyl-	Terpenoids	C19H40	-
907	AMW2904	3-Acetyl-2,5-dimethyl furan	Heterocyclic compounds	C8H10O2	sweet, musty, nutty, earthy, cocoa, corn, leathery
908	AMW0056	Sulfurous acid, 2-ethylhexyl isohexyl ester	Organic acid and Its derivatives	C14H30O3S	-
909	AMW2574	Heptadecane, 4-methyl-	Hydrocarbons	C18H38	-
910	AMW2459	Pentadecane, 5-methyl-	Hydrocarbons	C16H34	-
911	AMW3283	9-methylheptadecane	Hydrocarbons	C18H38	-
912	AMW3631	1H-[1,2,4]Triazolo[4,3-b][1,2,4]triazole-3,6-diamine	Alcohol and amines	C3H5N7	-
913	AMW0483	4-(2,6,6-Trimethylcyclohexa-1,3-dienyl)but-3-en-2-one	Aldehyde, Ketones, Esters	C13H18O	-
914				C11H12N2	
	AMW3829	Propanoic acid, 2,2-dimethyl-, 2,4-dinitrophenyl ester	Aldehyde, Ketones, Esters	O6	-
915	AMW3788	4H-1,2,4-Triazol-3-amine, 4-propyl-	Alcohol and amines	C5H10N4	-
916	AMW2610	2(3H)-Furanone, 5-acetyldihydro-	Aldehyde, Ketones, Esters	C6H8O3	-
917				C14H19NO	
	AMW3409	Piperoxan	Heterocyclic compounds	2	-
918	AMW0198	Benzene, 1-methoxy-4-propyl-	Benzene and substituted derivatives	C10H14O	sweet, anisic, licorice, sassafrass, fennel
919	AMW0059	Sulfurous acid, 2-ethylhexyl hexyl ester	Aldehyde, Ketones, Esters	C14H30O3S	-
920	AMW0936	4(1H)-Pyrimidinone, 1-methyl-	Aldehyde, Ketones, Esters	C5H6N2O	-
921	AMW1660	Octadecane	Hydrocarbons	C18H38	alkane
922				C8H12Cl2O	
	AMW2784	4-Chlorobutanoic anhydride	Organic acid and Its derivatives	3	-
923	AMW0535	Decane	Hydrocarbons	C10H22	alkane

924	AMW0094	Nonanoic acid, 3-methylphenyl ester	Aldehyde, Ketones, Esters	C16H24O2	-
925	AMW1185	Tridecane, 1-iodo-	Halogenated hydrocarbons	C13H27I	-
926	AMW1141	3-Hydroxydecanoic acid	Organic acid and Its derivatives	C10H20O3	-
927	AMW3134	1,4-Benzenediamine, N,N-dimethyl-	Alcohol and amines	C8H12N2	-
928	AMW3851	Formamide, N,N'-1,3-propanediylbis-	Alcohol and amines	C5H10N2O2	-
929	AMW3375	2-Propenoic acid, (1-methyl-1,2-ethanediyl)bis[oxy(methyl-2,1-ethanediyl)] ester	Aldehyde, Ketones, Esters	C15H24O6	-
930	AMW1543	cis-9-Hexadecenal	Aldehyde, Ketones, Esters	C16H30O	-
931	AMW4428	1,3,5-Trimethyl-2-cyclopentylbenzene	Benzene and substituted derivatives	C14H20	-
932	AMW0669	Heptadecane, 2-methyl-	Hydrocarbons	C18H38	-
933	AMW1514	N-methyl-N-nitroso-1-Dodecanamine	Alcohol and amines	C13H28N2O	-
934	AMW4135	l-Proline, N-methoxycarbonyl-, isohexyl ester	Aldehyde, Ketones, Esters	C13H23NO4	-
935	AMW4210	Butyl 2-(2-(2-butoxyethoxy)ethoxy)acetate	Aldehyde, Ketones, Esters	C14H28O5	-
936	AMW0569	5-Fluoro-1,3-benzoxazol-2(3H)-one	Aldehyde, Ketones, Esters	C7H4FNO2	-
937	AMW1830	Nonadecane	Hydrocarbons	C19H40	sweet, rosy
938	AMW2182	2,5-Diethylphenol	Benzene and substituted derivatives	C10H14O	-
939	AMW2958	(2S,6R,7S,8E)-(+)-2,7-Epoxy-4,8-megastigmadiene	Heterocyclic compounds	C13H20O	-
940	AMW4423	2-Ethylbutyric acid, 2,6-dimethoxyphenyl ester	Aldehyde, Ketones, Esters	C14H20O4	-
941	AMW3594	Carbonic acid, bis(2-ethylhexyl) ester	Aldehyde, Ketones, Esters	C17H34O3	-
942	AMW1452	2-deoxy-D-erythro-Pentose	Aldehyde, Ketones, Esters	C5H10O4	-
943	AMW0591	2-ethenyl-6-methyl-Pyrazine	Heterocyclic compounds	C7H8N2	hazelnut
944	AMW1397	Hexadecane, 2,6,11,15-tetramethyl-	Hydrocarbons	C20H42	-
945	AMW0435	1-Octadecene	Hydrocarbons	C18H36	-

946	AMW1358	3-Ethyl-7-hydroxyphthalide	Aldehyde, Ketones, Esters	$C_{10}H_{10}O_3$	-
947	AMW0169	(1R,2S,4r)-4-((E)-prop-1-en-1-yl)cyclopentane-1,2-diol	Alcohol and amines	$C_8H_{14}O_2$	-
948	AMW0063	Oxalic acid, cyclohexylmethyl ethyl ester	Organic acid and Its derivatives	$C_{11}H_{18}O_4$	-
949	AMW3231	1,15-Hexadecadiene	Hydrocarbons	$C_{16}H_{30}$	-
950	AMW2835	4H-1,3-Dioxin-4-one, 2-(1,1-dimethylethyl)-6-methyl-, (R)-	Aldehyde, Ketones, Esters	$C_9H_{14}O_3$	-
951	AMW0840	1-iodo-Tetradecane	Halogenated hydrocarbons	$C_{14}H_{29}I$	-
952	AMW2826	1,1,4,5,6-Pentamethyl-2,3-dihydro-1H-indene	Benzene and substituted derivatives	$C_{14}H_{20}$	-
953	AMW1596	.beta.-Methoxy-.alpha.-phenylphenethyl alcohol	Alcohol and amines	$C_{15}H_{16}O_2$	-
954	AMW3844	9-Oxa-bicyclo[3.3.1]nonane-2,6-dione	Aldehyde, Ketones, Esters	$C_8H_{10}O_3$	-
955	AMW3716	.alpha.-Cuprenene	Terpenoids	$C_{15}H_{24}$	-
956	AMW0623	Azacyclohexane, 3-[1-pyrrolidyl]-	Heterocyclic compounds	$C_9H_{18}N_2$	-
957	AMW4301	Benzyl alcohol, .alpha.-(1-(dimethylamino)ethyl)-	Alcohol and amines	$C_{11}H_{17}NO$	-
958	AMW3391	1,4-Benzenedicarboxylic acid, dimethyl ester	Aldehyde, Ketones, Esters	$C_{10}H_{10}O_4$	-
959	AMW1980	Tridecane, 7-hexyl-	Hydrocarbons	$C_{19}H_{40}$	-
960	AMW3137	3,6-Heptanedione	Aldehyde, Ketones, Esters	$C_7H_{12}O_2$	-
961	AMW2989	1-(1,3-Dimethyl-1H-pyrazol-4-yl)ethanone	Aldehyde, Ketones, Esters	$C_7H_{10}N_2O$	-
962	AMW4510	2-[2-(4-Methyl-furazan-3-yloxy)-ethyl]-2H-tetrazol-5-ylamine	Alcohol and amines	$C_6H_9N_7O_2$	-
963	AMW4478	5-.alpha.-Aminopropyltetrazole	Heterocyclic compounds	$C_4H_9N_5$	-
964	AMW2481	4-Acetyl-1-methylcyclohexene	Hydrocarbons	$C_9H_{14}O$	-
965	AMW3775	Bicyclo[3.3.0]octan-3-one, 6-hydroxy-6-methyl-	Aldehyde, Ketones, Esters	$C_9H_{14}O_2$	-
966	AMW1131	4-tert-Butylcyclohexyl acetate	Aldehyde, Ketones, Esters	$C_{12}H_{22}O_2$	woody, cedary, floral, oily, herbal, balsamic, green, fruity
967	AMW1469	m-Aminophenylacetylene	Alcohol and amines	C_8H_7N	-
968	AMW2858	Pentadecane, 8-hexyl-	Hydrocarbons	$C_{21}H_{44}$	-
969	AMW3548	Tridecane, 6-propyl-	Hydrocarbons	$C_{16}H_{34}$	-
970	AMW0078	4-Butylbenzoic acid, 2-dimethylaminoethyl ester	Aldehyde, Ketones, Esters	$C_{15}H_{23}NO$	-

				2	
971	AMW0144	Decane, 3-chloro-	Halogenated hydrocarbons	C10H21Cl	-
972	AMW3205	4',6'-Dimethoxy-2',3'-dimethylacetophenone	Aldehyde, Ketones, Esters	C12H16O3	-
973	AMW0105	Heptadecanal	Aldehyde, Ketones, Esters	C17H34O	-
974	AMW0298	Isopropyl acetate	Aldehyde, Ketones, Esters	C5H10O2	etherial, fruity, sweet, banana, chemical
975	AMW0156	Benzonitrile	Benzene and substituted derivatives	C7H5N	almond
976	AMW4172	Thiazole, 2-(phenylthio)-	Heterocyclic compounds	C9H7NS2	-
977	AMW2177*050	2-ethyl-Thiophene	Heterocyclic compounds	C6H8S	styrene
978	AMW1721*194	1,1-diethyl-Hydrazine	Nitrogen compounds	C4H12N2	-
979	AMW5151*300	2-Hexadecene, 2,6,10,14-tetramethyl-	Hydrocarbons	C20H40	-
980	AMW4727	3-Thiophenecarboxaldehyde	Aldehyde, Ketones, Esters	C5H4OS	-
981	AMW2597	Benzene, (2-methylpropyl)-	Benzene and substituted derivatives	C10H14	-
982	AMW3941	Octane, 2-bromo-	Halogenated hydrocarbons	C8H17Br	-
983	AMW3624	Cyclopentane, 1-butyl-2-ethyl-	Hydrocarbons	C11H22	-
984	AMW3961	Ethylene glycol di-n-butyrate	Aldehyde, Ketones, Esters	C10H18O4	-
985	AMW0797	Bicyclo[3.1.1]hept-2-ene, 2,6-dimethyl-6-(4-methyl-3-pentenyl)-	Terpenoids	C15H24	-
986	AMW2694	Succinic acid, 1,1,1-trifluoroprop-2-yl 4-octyl ester	Aldehyde, Ketones, Esters	C15H25F3O4	-
987	AMW4513	1-Pentene, 2,3-dimethyl-	Hydrocarbons	C7H14	-
988	AMW2881	Phenylglyoxylic acid, isopropyl ester	Aldehyde, Ketones, Esters	C11H12O3	-
989	AMW0970	Cyclohexanone, 2,2,6-trimethyl-	Aldehyde, Ketones, Esters	C9H16O	pungent, thujone, labdanum, honey, cistus
990	AMW0699	3,5-di-tert-Butyl-4-hydroxybenzAldehyde	Aldehyde, Ketones, Esters	C15H22O2	-
991	AMW1769	Propanoic acid, pentyl ester	Aldehyde, Ketones, Esters	C8H16O2	sweet, fruity, apricot, pineapple, tropical, banana
992	AMW2688	2-Pyrrolidinemethanol, 1-acetyl-, acetate (ester)	Aldehyde, Ketones, Esters	C9H15NO3	-
993	AMW1580	3-Hydroxy-4-methylbenzAldehyde	Aldehyde, Ketones, Esters	C8H8O2	-

994	AMW1862	3-methyl-Tridecane	Hydrocarbons	C14H30	-
995	AMW2936	3-Methyl-1H-pyrazole-4-carbaldehyde	Aldehyde, Ketones, Esters	C5H6N2O	-
996	AMW4290	Phenol, 4,6-di(1,1-dimethylethyl)-2-methyl-	Benzene and substituted derivatives	C15H24O	-
997	AMW1705	Isopropyl phenyl ketone	Aldehyde, Ketones, Esters	C10H12O	green
998	AMW4047	3-Hydroxy-1-methylpyridinium hydroxide	Heterocyclic compounds	C6H9NO2	-
999	AMW2673	Pyridine, 3-methoxy-	Heterocyclic compounds	C6H7NO	-
1000	AMW4187	N-(Piperidin-3-yl)methanesulfonamide	Alcohol and amines	C6H14N2O2S	-
1001	AMW3430	1,2,4-Triazine-3,5(2H,4H)-dione	Aldehyde, Ketones, Esters	C3H3N3O2	-
1002	AMW2280	3-Formylamino succinimide	Alcohol and amines	C5H6N2O3	-
1003	AMW1104	1-Piperidineethanol	Alcohol and amines	C7H15NO	-
1004	AMW2387	Cyclohexene, 3-(1,5-dimethyl-4-hexenyl)-6-methylene-, [S-(R*,S*)]-	Terpenoids	C15H24	herbal, fruity, woody
1005	AMW3762	Cyclododecanol	Alcohol and amines	C12H24O	-
1006	AMW0122	(2E)-6Acetoxy-2-methylhexenal	Aldehyde, Ketones, Esters	C9H14O3	-
1007	AMW4299	cis-2,4-Dimethylthiane, S,S-dioxide	Heterocyclic compounds	C7H14O2S	-
1008	AMW4783	Heptane, 4-ethyl-2,2,6,6-tetramethyl-	Hydrocarbons	C13H28	-
1009	AMW4076	Resorcinol monoacetate	Aldehyde, Ketones, Esters	C8H8O3	-
1010	AMW3361	3-Butene-1,2-diol, 1-(2-furanyl)-	Alcohol and amines	C8H10O3	-
1011	AMW3710	l-Alanine, N-ethoxycarbonyl-, pentyl ester	Aldehyde, Ketones, Esters	C11H21NO4	-
1012	AMW5090	N-Thio-valero-morpholine	Heterocyclic compounds	C9H17NOS	-
1013	AMW0419	1-Undecanol	Alcohol and amines	C11H24O	fresh, waxy, rose, soapy, clean, cloth, floral, citrus
1014	AMW0453	1-Hexyl-1-nitrocyclohexane	Nitrogen compounds	C12H23NO2	-
1015	AMW3026	3-Methylbut-2-enoic acid, 4-nitrophenyl ester	Aldehyde, Ketones, Esters	C11H11NO	-

1016	AMW1828	Hexadecanal	Aldehyde, Ketones, Esters	C16H32O	cardboard
1017	AMW4007	Cyclohexane, undecyl-	Hydrocarbons	C17H34	-
1018	AMW3453	10-Methylnonadecane	Hydrocarbons	C20H42	-
1019	AMW1826	n-Pentadecanol	Alcohol and amines	C15H32O	-
1020	AMW2245*110	Benzene, 1,2,4,5-tetramethyl-	Benzene and substituted derivatives	C10H14	rancid, sweet
1021	AMW2188*374	(2-methyl-1-propenyl)-Cyclohexane	Hydrocarbons	C10H18	-
1022	AMW1744*107	2,4,6-trimethyl-Decane	Hydrocarbons	C13H28	-
1023	AMW1223*071	2,3,4-Trimethylpyrrole	Heterocyclic compounds	C7H11N	-
1024	AMW1947*179	cis-2-(2-Pentenyl)furan	Heterocyclic compounds	C9H12O	-
1025	AMW5083	1-Penten-3-one, 1-(2,6,6-trimethyl-1-cyclohexen-1-yl)-	Terpenoids	C14H22O	orris, woody, powdery, floral, violet, tropical, tobacco, creamy
1026	AMW2534	1,2,4,5-Tetrazine	Heterocyclic compounds	C2H2N4	-
1027	AMW1501	3-(Ehtylthio)propanal	Aldehyde, Ketones, Esters	C5H10OS	-
1028	AMW2123	Propanamide	Alcohol and amines	C3H7NO	-
1029	AMW4132	Ethanone, 2-hydroxy-1-phenyl-	Aldehyde, Ketones, Esters	C8H8O2	-
1030	AMW2690	4-Isopropylcyclohexylamine	Alcohol and amines	C9H19N	-
1031	AMW0403	2-methyl-Cyclopentanone	Aldehyde, Ketones, Esters	C6H10O	roasted, beefy
1032	AMW3714	Benzene, 1-ethyl-4-methoxy-	Ethers	C9H12O	anisic
1033	AMW0633	Pyrazine, trimethyl-	Heterocyclic compounds	C7H10N2	nut skin, earthy, powdery, cocoa, baked, potato, roasted, peanut, hazelnut, musty
1034	AMW3167	(1-Propoxy-pentyl)-cyclopropane	Ethers	C11H22O	-
1035	AMW2076	3-Hexen-2-one	Aldehyde, Ketones, Esters	C6H10O	-
1036	AMW1684	Phenylethyl Alcohol	Alcohol and amines	C8H10O	fruity, rose, sweet, apple
1037	AMW4728	Pyrazine, 2-ethyl-3-methyl-	Heterocyclic compounds	C7H10N2	Green, must, nut, potato, roast
1038	AMW4786	2-Furaldehyde diethyl acetal	Aldehyde, Ketones, Esters	C9H14O3	fruity, earthy, mushroom
1039	AMW0883	1,11-Dodecadiyne	Hydrocarbons	C12H18	-

1040	AMW3311	2,2-Dimethyl-3-morpholin-4-ylpropan-1-amine	Alcohol and amines	C9H20N2O	-
1041	AMW2142	3-methyl-3-Buten-2-one	Aldehyde, Ketones, Esters	C5H8O	-
1042	AMW0736	ethyl-Cyclohexane	Hydrocarbons	C8H16	-
1043	AMW3469	Amyl crotonate	Aldehyde, Ketones, Esters	C9H16O2	-
1044	AMW3075	Glutaraldehyde	Aldehyde, Ketones, Esters	C5H8O2	-
1045	AMW4119	Cyclohexanol, 1-ethenyl-	Alcohol and amines	C8H14O	-
1046	AMW4738	2-Hexen-1-ol, acetate, (E)-	Aldehyde, Ketones, Esters	C8H14O2	Sweet herbaceous, fruity, banana peel, unripe
1047	AMW0117	Hexadecyl pentyl ether	Ethers	C21H44O	-
1048	AMW0726	Ethanol, 2-(diethylamino)-, N-oxide	Alcohol and amines	C6H15NO2	-
1049	AMW1277	2-methyl-Benzofuran	Heterocyclic compounds	C9H8O	burnt, phenol
1050	AMW3754	2-Methyl-pyrrolidine-2-carboxylic acid	Organic acid and Its derivatives	C6H11NO2	-
1051	AMW2282*166	Carvone	Terpenoids	C10H14O	minty, licorice
1052	AMW4919*166	(-)-Carvone	Terpenoids	C10H14O	sweet, spearmint, herbal, minty
1053	AMW2456*365	Decane, 2,3,8-trimethyl-	Hydrocarbons	C13H28	-
1054	AMW0925*092	2-Octen-1-ol	Alcohol and amines	C8H16O	green, vegetable
1055	AMW1017*092	(Z)-2-Octen-1-ol	Alcohol and amines	C8H16O	sweet, floral
1056	AMW1457*087	Benzene, 1-methyl-3-(1-methylethyl)-	Benzene and substituted derivatives	C10H14	-
1057	AMW2289*087	p-Cymene	Benzene and substituted derivatives	C10H14	woody, citrus
1058	AMW4729*232	2-Hexenoic acid, (E)-	Organic acid and Its derivatives	C6H10O2	Sweaty, body odour, fruity
1059	AMW4745*232	2-Hexenoic acid	Organic acid and Its derivatives	C6H10O2	Musty, fatty, sweaty
1060	AMW0507*387	BenzAldehyde, 4-methoxy-	Aldehyde, Ketones, Esters	C8H8O2	sweet, powdery, mimosa, floral, hawthorn, balsamic
1061	AMW2365*287	Bicyclo[2.2.1]heptan-2-ol, 1,7,7-trimethyl-, acetate, (1S-endo)-	Aldehyde, Ketones, Esters	C12H20O2	sweet, balsamic, woody, fresh, pine, needle, herbal
1062	AMW2373*287	Bornyl acetate	Aldehyde, Ketones, Esters	C12H20O2	woody, pine, herbal, cedary, spice
1063	AMW3044*339	trans-Ocimenol	Terpenoids	C10H18O	-
1064	AMW1099*069	3,5-Octadien-2-one, (E,E)-	Aldehyde, Ketones, Esters	C8H12O	fruity, green, grassy

1065	AMW0497*112	1-(4-methylphenyl)-Ethanone	Aldehyde, Ketones, Esters	C9H10O	green, pea, bell pepper, galbanum
1066	AMW2839*353	2,3-Dimethyldodecane	Hydrocarbons	C14H30	-
1067	AMW2922*353	Tridecane, 4-methyl-	Hydrocarbons	C14H30	-
1068	AMW1937*096	(Z)-3-Nonen-2-ol	Alcohol and amines	C9H18O	-
1069	AMW0557*088	Decane, 5-methyl-	Hydrocarbons	C11H24	-
1070	AMW1060*088	Decane, 4-methyl-	Hydrocarbons	C11H24	pungent
1071	AMW1941*088	2-methyl-Decane	Hydrocarbons	C11H24	-
1072	AMW4882*367	Decane, 2,5,6-trimethyl-	Hydrocarbons	C13H28	-
1073	AMW2273*113	.alpha.-Terpineol	Terpenoids	C10H18O	pine, iris, teil
1074	AMW4686*211	δ-valerolactone	Aldehyde, Ketones, Esters	C5H8O2	estery, fruity, sweet
1075	AMW2010*345	7-methyl-1-Undecene	Hydrocarbons	C12H24	-
1076	AMW4862*345	4-Undecene, 5-methyl-, (E)-	Hydrocarbons	C12H24	-
1077	AMW0234*125	2,6-Octadien-1-ol, 3,7-dimethyl-, (Z)-	Terpenoids	C10H18O	lemon, fresh
1078	AMW4915*125	3,6-Octadien-1-ol, 3,7-dimethyl-, (Z)-	Terpenoids	C10H18O	rose
1079	AMW2601*363	(3aS,8aS)-6,8a-Dimethyl-3-(propan-2-ylidene)-1,2,3,3a,4,5,8,8a-octahydroazulene	Terpenoids	C15H24	-
1080	AMW4481*263	(3Z,5E)-1,3,5-Undecatriene	Hydrocarbons	C11H18	-
1081	AMW2233*393	4-ethyl-1,2-dimethyl-Benzene	Hydrocarbons	C10H14	-
1082	AMW3158*393	Benzene, 2-ethyl-1,3-dimethyl-	Benzene and substituted derivatives	C10H14	-
1083	AMW2383*338	Isoborneol	Terpenoids	C10H18O	balsamic, camphor, herbal, woody
1084	AMW2520*338	Bicyclo[2.2.1]heptan-2-ol, 1,7,7-trimethyl-, (1S-endo)-	Terpenoids	C10H18O	pine, woody, camphor
1085	AMW4721*344	Propanoic acid, 2-methyl-, 1-methylbutyl ester	Aldehyde, Ketones, Esters	C9H18O2	-
1086	AMW0705*347	5,6-dimethyl-Decane	Hydrocarbons	C12H26	-
1087	AMW3333*107	Decane, 2,5,9-trimethyl-	Hydrocarbons	C13H28	-
1088	AMW0311*236	Phenol, 3,5-dimethyl-	Benzene and substituted derivatives	C8H10O	balsamic, coffee
1089	AMW2607*359	1H-3a,7-Methanoazulene, octahydro-3,8,8-trimethyl-6-	Terpenoids	C15H24	-

		methylene-, [3R-(3.alpha.,3a.beta.,7.beta.,8a.alpha.)]-			
1090	AMW1741*068	2,4,6-trimethyl-Octane	Hydrocarbons	C11H24	-
1091	AMW2457*254	2,6-Nonadienal, (E,E)-	Aldehyde, Ketones, Esters	C9H14O	fresh, citrus, green, cucumber, melon
1092	AMW2350*273	Ethanol, 2-(2-butoxyethoxy)-	Alcohol and amines	C8H18O3	-
1093	AMW3011*221	p-Aminotoluene	Alcohol and amines	C7H9N	-
1094	AMW4924*366	Undecane, 2,3-dimethyl-	Hydrocarbons	C13H28	-
1095	AMW1707*366	Dodecane, 4-methyl-	Hydrocarbons	C13H28	-
1096	AMW3470*366	Decane, 5-propyl-	Hydrocarbons	C13H28	-
1097	AMW2569*366	Dodecane, 2-methyl-	Hydrocarbons	C13H28	-
1098	AMW4813*311	Benzene, 1,3-dimethyl-5-(1-methylethyl)-	Benzene and substituted derivatives	C11H16	-
1099	AMW1341*311	2,4-dimethyl-1-(1-methylethyl)-Benzene	Benzene and substituted derivatives	C11H16	-
1100	AMW2511*372	Nonane, 2,3-dimethyl-	Hydrocarbons	C11H24	-
1101	AMW3273*326	2-Carene	Terpenoids	C10H16	-
1102	AMW1080*326	(+)-4-Carene	Terpenoids	C10H16	-
1103	AMW0279*099	1-methyl-4-propyl-Benzene	Benzene and substituted derivatives	C10H14	-
1104	AMW2180*105	1-ethyl-2,4-dimethyl-Benzene	Benzene and substituted derivatives	C10H14	-
1105	AMW1298*051	3-Furanmethanol	Alcohol and amines	C5H6O2	-
1106	AMW0608*100	1,3-diethyl-Benzene	Benzene and substituted derivatives	C10H14	-
1107	AMW0214*100	Benzene, 1,4-diethyl-	Benzene and substituted derivatives	C10H14	-
1108	AMW4593*214	3-Hexen-1-ol, (E)-	Alcohol and amines	C6H12O	green floral
1109	AMW2285*329	.alpha.-Phellandrene 1	Terpenoids	C10H16	citrus, herbal, terpene, green, woody, peppery
1110	AMW2288*329	1,3-Cyclohexadiene, 1-methyl-4-(1-methylethyl)-	Terpenoids	C10H16	woody, terpene, lemon, herbal, medicinal, citrus
1111	AMW4603*319	Heptane, 3-ethyl-	Hydrocarbons	C9H20	-
1112	AMW1757*117	3,3,6-trimethyl-Decane	Hydrocarbons	C13H28	-
1113	AMW1742*117	2,6,7-trimethyl-Decane	Hydrocarbons	C13H28	-
1114	AMW1756*117	3,3,5-trimethyl-Decane	Hydrocarbons	C13H28	-

1115	AMW3150*117	Decane, 3,3,8-trimethyl-	Hydrocarbons	C13H28	-
1116	AMW0760*335	3-Ethyl-3-methylheptane	Hydrocarbons	C10H22	-
1117	AMW2159*122	6-methyl-5-Undecene	Hydrocarbons	C12H24	-
1118	AMW2012*122	(Z)-4-methyl-4-Undecene	Hydrocarbons	C12H24	-
1119	AMW1804*377	Hexanamide	Alcohol and amines	C6H13NO	-
1120	AMW4764*330	Cyclooctane, 1,4-dimethyl-, cis-	Hydrocarbons	C10H20	-
1121	AMW0189*118	3-Nonen-1-ol, (Z)-	Alcohol and amines	C9H18O	fresh, waxy, green, melon, rind, tropical, mushroom
1122	AMW4681*226	2-Heptenal, (Z)-	Aldehyde, Ketones, Esters	C7H12O	fried, roasted meat
1123	AMW2317*381	3-Hexen-1-ol, acetate, (Z)-	Aldehyde, Ketones, Esters	C8H14O2	fresh, green, sweet, fruity, banana, apple, grassy
1124	AMW4675*332	4-Heptanone, 2,6-dimethyl-	Aldehyde, Ketones, Esters	C9H18O	Green
1125	AMW1534*237	3,4,5-trimethyl-2-Cyclopenten-1-one	Aldehyde, Ketones, Esters	C8H12O	-
1126	AMW1746*346	3-ethyl-2,7-dimethyl-Octane	Hydrocarbons	C12H26	-
1127	AMW3385*351	Pentyl pentanoate	Aldehyde, Ketones, Esters	C10H20O2	ripe fruit, apple
1128	AMW2397*368	Undecane, 6-ethyl-	Hydrocarbons	C13H28	-
1129	AMW2963*368	Undecane, 3,9-dimethyl-	Hydrocarbons	C13H28	-
1130	AMW2726*368	Undecane, 4-ethyl-	Hydrocarbons	C13H28	-
1131	AMW5046*316	Naphthalene, 1,2,4a,5,6,8a-hexahydro-4,7-dimethyl-1-(1-methylethyl)-	Benzene and substituted derivatives	C15H24	-
1132	AMW2207*318	2,3,4-trimethyl-Hexane	Hydrocarbons	C9H20	-
1133	AMW1110*318	2,3-dimethyl-Heptane	Hydrocarbons	C9H20	-
1134	AMW1546*111	Terpinen-4-ol	Terpenoids	C10H18O	turpentine, nutmeg, musty
1135	AMW0778*369	Undecane, 5,7-dimethyl-	Hydrocarbons	C13H28	-
1136	AMW1843*045	1H-Pyrrole, 2-methyl-	Heterocyclic compounds	C5H7N	-
1137	AMW3521*253	3-Cyclohexen-1-one, 2,5,5-trimethyl-	Aldehyde, Ketones, Esters	C9H14O	-
1138	AMW4751*324	Cyclohexene, 1-methyl-4-(1-methylethenyl)-, (S)-	Terpenoids	C10H16	Fresh, citrus, oranges
1139	AMW2619*327	Bicyclo[3.1.0]hex-2-ene, 4-methyl-1-(1-methylethyl)-	Terpenoids	C10H16	-

1140	AMW4937*233	Fumaric Acid	Organic acid and Its derivatives	C4H4O4	-
1141	AMW0874*102	Benzene, 1-ethenyl-3,5-dimethyl-	Benzene and substituted derivatives	C10H12	-
1142	AMW2153*102	1-Phenyl-1-butene	Benzene and substituted derivatives	C10H12	-
1143	AMW0418*102	Benzene, 1-methyl-3-(1-methylethenyl)-	Benzene and substituted derivatives	C10H12	-
1144	AMW0474*102	Benzene, 1-methyl-4-(1-methylethenyl)-	Benzene and substituted derivatives	C10H12	phenol, spicy, clove, guaiacol
1145	AMW3171*288	(-)-Neomenthyl acetate	Aldehyde, Ketones, Esters	C12H22O2	-
1146	AMW2187*288	Menthyl acetate	Aldehyde, Ketones, Esters	C12H22O2	tea, minty, fruity
1147	AMW2908*207	4H-1,2,4-Triazol-3-amine, 4-methyl-	Alcohol and amines	C3H6N4	-
1148	AMW0564*079	Pyrazine, 2-ethyl-5-methyl-	Heterocyclic compounds	C7H10N2	coffee, beany, nutty, grassy, roasted
1149	AMW0627*184	3-methyl-1H-Pyrazole	Heterocyclic compounds	C4H6N2	-
1150	AMW2390*119	Undecane, 2,4-dimethyl-	Hydrocarbons	C13H28	-
1151	AMW0758*119	Undecane, 4,7-dimethyl-	Hydrocarbons	C13H28	-
1152	AMW2426*119	Undecane, 2,5-dimethyl-	Hydrocarbons	C13H28	-
1153	AMW2557*119	Undecane, 3,6-dimethyl-	Hydrocarbons	C13H28	-
1154	AMW2705*119	Undecane, 2,7-dimethyl-	Hydrocarbons	C13H28	-
1155	AMW2813*119	Undecane, 3,7-dimethyl-	Hydrocarbons	C13H28	-
1156	AMW3124*119	Undecane, 2,10-dimethyl-	Hydrocarbons	C13H28	-
1157	AMW0775*126	Undecane, 6,6-dimethyl-	Hydrocarbons	C13H28	-
1158	AMW0302*083	Phenol, 3-methyl-	Benzene and substituted derivatives	C7H8O	smoky, petroleum
1159	AMW2551*278	1H-Indene, 2,3-dihydro-1,1,4,5-tetramethyl-	Benzene and substituted derivatives	C13H18	-
1160	AMW0996*073	cis-2,6-Dimethyl-2,6-octadiene	Hydrocarbons	C10H18	-
1161	AMW1018*073	2,6-Dimethyl-2-trans-6-octadiene	Terpenoids	C10H18	-
1162	AMW4753*348	2,2,7,7-Tetramethyloctane	Hydrocarbons	C12H26	-
1163	AMW0763*074	Nonane, 2,6-dimethyl-	Hydrocarbons	C11H24	-
1164	AMW0764*074	Nonane, 3,7-dimethyl-	Hydrocarbons	C11H24	-
1165	AMW0844*085	cis-3-Decene	Hydrocarbons	C10H20	-

1166	AMW0854*085	5-Decene	Hydrocarbons	C10H20	-
1167	AMW4707*085	3-Decene	Hydrocarbons	C10H20	-
1168	AMW0853*085	4-Decene	Hydrocarbons	C10H20	-
1169	AMW0873*085	(Z)-2-Decene	Hydrocarbons	C10H20	-
1170	AMW1054*224	3-ethyl-Cyclohexene	Hydrocarbons	C8H14	-
1171	AMW1093*317	.gamma.-Muurolene	Terpenoids	C15H24	herbal, woody, spice
1172	AMW2911*317	Naphthalene, 1,2,4a,5,8,8a-hexahydro-4,7-dimethyl-1-(1-methylethyl)-, (1.alpha.,4a.beta.,8a.alpha.)-(.+/-.)-	Terpenoids	C15H24	-
1173	AMW2492*317	Naphthalene, 1,2,4a,5,6,8a-hexahydro-4,7-dimethyl-1-(1-methylethyl)-, (1.alpha.,4a.alpha.,8a.alpha.)-	Terpenoids	C15H24	-
1174	AMW4890*252	Creosol	Benzene and substituted derivatives	C8H10O2	smoky
1175	AMW4943*283	3-Tridecene, (Z)-	Hydrocarbons	C13H26	-
1176	AMW0729*255	2,7-dimethyl-2,6-Octadiene	Hydrocarbons	C10H18	-
1177	AMW2535*308	Undecane, 4,8-dimethyl-	Hydrocarbons	C13H28	-
1178	AMW2872*308	Undecane, 5,6-dimethyl-	Hydrocarbons	C13H28	-
1179	AMW0757*308	Undecane, 3,8-dimethyl-	Hydrocarbons	C13H28	-
1180	AMW4891*272	1-Octanol, 3,7-dimethyl-	Alcohol and amines	C10H22O	waxy, soapy, aldehydic, leathery, musty, citrus, green
1181	AMW0310*084	Mesitylene	Benzene and substituted derivatives	C9H12	-
1182	AMW2135*142	trans-.beta.-Ionone	Terpenoids	C13H20O	dry, powdery, floral, woody, orris
1183	AMW1014*386	8-Heptadecene	Hydrocarbons	C17H34	-
1184	AMW1700*132	Dodecane, 4,6-dimethyl-	Hydrocarbons	C14H30	-
1185	AMW0767*109	3,6-dimethyl-Decane	Hydrocarbons	C12H26	-
1186	AMW0769*109	3,8-dimethyl-Decane	Hydrocarbons	C12H26	-
1187	AMW4871*104	Phenol, 3-ethyl-	Benzene and substituted derivatives	C8H10O	phenolic, smoky
1188	AMW2407*357	(1R,5R)-2-Methyl-5-((R)-6-methylhept-5-en-2-yl)bicyclo[3.1.0]hex-2-ene	Terpenoids	C15H24	-

1189	AMW2994*282	trans-1,10-Dimethyl-trans-9-decalinol	Alcohol and amines	C12H22O	-
1190	AMW3971*312	cis-Muurola-4(15),5-diene	Terpenoids	C15H24	-
1191	AMW2042*098	1-ethenyl-3-ethyl-Benzene	Benzene and substituted derivatives	C10H12	-
1192	AMW0874*103	2-ethenyl-1,3-dimethyl-Benzene	Benzene and substituted derivatives	C10H12	-
1193	AMW1922*022	(E)-2-methyl-3-Hexene	Hydrocarbons	C7H14	-
1194	AMW1199*022	(Z)-4-Methyl-2-hexene	Hydrocarbons	C7H14	-
1195	AMW1200*022	(E)-4-methyl-2-Hexene	Hydrocarbons	C7H14	-
1196	AMW1159*022	4-Methyl-2-hexene,c&t	Hydrocarbons	C7H14	-
1197	AMW4955*257	Naphthalene, 1-methyl-	Benzene and substituted derivatives	C11H10	naphthyl, chemical, medicinal, camphor
1198	AMW1520*266	(+)-Dihydrocarvone	Terpenoids	C10H16O	herbal, minty, menthol
1199	AMW0959*285	2-Buten-1-one, 1-(2,6,6-trimethyl-1,3-cyclohexadien-1-yl)-, (E)-	Terpenoids	C13H18O	apple, rose, honey, tobacco, sweet
1200	AMW2539*274	trans-Isoeugenol	Benzene and substituted derivatives	C10H12O2	floral, clove
1201	AMW2523*293	Tridecane, 2,5-dimethyl-	Hydrocarbons	C15H32	-
1202	AMW2376*378	1-Octene, 2-methyl-	Hydrocarbons	C9H18	-
1203	AMW4910*382	2-Cyclohexen-1-ol, 2-methyl-5-(1-methylethenyl)-, (1S-trans)-	Alcohol and amines	C10H16O	-
1204	AMW0714*350	4-Ethyloctanoic acid	Organic acid and Its derivatives	C10H20O2	goaty, lamb, fatty
1205	AMW1875*168	7-Tetradecenal, (Z)-	Hydrocarbons	C14H26O	-
1206	AMW5170*151	9-Hexadecenoic acid	Organic acid and Its derivatives	C16H30O2	-
1207	AMW1209*151	Palmitoleic Acid	Organic acid and Its derivatives	C16H30O2	-
1208	AMW3246*206	3-Isoxazolamine, 5-methyl-	Alcohol and amines	C4H6N2O	-
1209	AMW0872*261	2-Propanol, 1-(2-methoxy-1-methylethoxy)-	Alcohol and amines	C7H16O3	-
1210	AMW0307*095	1,3-diethenyl-Benzene	Benzene and substituted derivatives	C10H10	-
1211	AMW4983*258	Quinoline, 5-methyl-	Benzene and substituted derivatives	C10H9N	-
1212	AMW2695*259	3-Vinyl-1,2-dithiacyclohex-5-ene	Heterocyclic compounds	C6H8S2	-

1213	AMW2486*289	4-Decenoic acid, ethyl ester, (Z)-	Aldehyde, Ketones, Esters	C12H22O2	-
1214	AMW2259*144	2,4-Di-tert-butylphenol	Benzene and substituted derivatives	C14H22O	phenol
1215	AMW5057*144	Phenol, 2,5-bis(1,1-dimethylethyl)-	Benzene and substituted derivatives	C14H22O	-
1216	AMW2531*243	2-Propyl-1-pentanol	Alcohol and amines	C8H18O	-
1217	AMW3353*222	1-(3H-Imidazol-4-yl)-ethanone	Heterocyclic compounds	C5H6N2O	-
1218	AMW4974*277	Naphthalene, 1,2-dihydro-1,5,8-trimethyl-	Benzene and substituted derivatives	C13H16	-
1219	AMW0093*277	1, 1, 5-Trimethyl-1, 2-dihydronaphthalene	Benzene and substituted derivatives	C13H16	-
1220	AMW0590*101	Pyrazine, 2-ethyl-3,5-dimethyl-	Heterocyclic compounds	C8H12N2	burnt, almond, roasted, nutty, coffee
1221	AMW1436*161	(-)-.beta.-Bourbonene	Terpenoids	C15H24	herbal, woody, floral, balsamic
1222	AMW5060*290	4-isopropyl-1,6-dimethyl-1,2,3,4-tetrahydronaphthalene	Benzene and substituted derivatives	C15H22	-
1223	AMW2482*290	trans-Calamenene	Terpenoids	C15H22	-
1224	AMW2191*337	Piperitones	Terpenoids	C10H16O	herbal, minty, camphor, medicinal
1225	AMW1850*050	Thiophene, 2,4-dimethyl-	Heterocyclic compounds	C6H8S	fried, onion
1226	AMW1839*050	3,4-dimethyl-Thiophene	Heterocyclic compounds	C6H8S	savory, roasted, onion
1227	AMW4532*194	Hydrazine, 1,2-diethyl-	Nitrogen compounds	C4H12N2	-
1228	AMW0980*300	3,7,11,15-Tetramethylhexadec-2-ene	Hydrocarbons	C20H40	-
1229	AMW1444*110	Benzene, 1,2,3,5-tetramethyl-	Benzene and substituted derivatives	C10H14	-
1230	AMW1364*110	1,2,3,4-tetramethyl-Benzene	Benzene and substituted derivatives	C10H14	-
1231	AMW0920*071	2,3,5-trimethyl-1H-Pyrrole	Heterocyclic compounds	C7H11N	-
1232	AMW0892*179	Furan, 2-(1-pentenyl)-, (E)-	Heterocyclic compounds	C9H12O	roasted
1233	AMW4724*179	trans-2-(2-Pentenyl)furan	Heterocyclic compounds	C9H12O	tomato-like

Note: "Index" is the Myvi ID of the substance (* indicates that the substance is isomeric with other substances of the same number). The aroma description of the substance is taken from (http://www.thegoodscentscompany.com) or (http://perflavory.com/) or (http://www.odour.org.uk/odour/index.html) or (http://foodflavorlab.cn/#/home) or literature, etc. "-" indicates that the flavor was not queried.

Table S2 509 significantly different compounds screened in CK, L and Y groups.

Number	Index	Compounds	Class I	Formula	VIP	P-value
1	AMW0940*166	D-Carvone	Terpenoids	C10H14O	1.530	0.000
2	AMW4914*387	Benzaldehyde, 2-methoxy-	Aldehyde, Ketones, Esters	C8H8O2	1.523	0.000
3	AMW4851*339	cis-Ocimenol	Terpenoids	C10H18O	1.484	0.002
4	AMW1219*069	3,5-Octadien-2-one	Aldehyde, Ketones, Esters	C8H12O	1.264	0.036
5	AMW3258*367	Nonane, 2-methyl-5-propyl-	Hydrocarbons	C13H28	1.401	0.001
6	AMW4684*211	2(3H)-Furanone, dihydro-4-methyl-	Aldehyde, Ketones, Esters	C5H8O2	1.275	0.000
7	AMW1115*345	5-methyl-5-Undecene	Hydrocarbons	C12H24	1.493	0.001
8	AMW0233*125	Geraniol	Terpenoids	C10H18O	1.412	0.008
9	AMW0999*269	2-Decenal, (Z)-	Aldehyde, Ketones, Esters	C10H18O	1.304	0.030
10	AMW2654*263	(3E,5Z)-1,3,5-Undecatriene	Hydrocarbons	C11H18	1.469	0.002
11	AMW2356*338	endo-Borneol	Terpenoids	C10H18O	1.459	0.003
12	AMW4849*270	Cyclohexanol, 1-methyl-4-(1-methylethenyl)-	Terpenoids	C10H18O	1.242	0.041
13	AMW0703*347	Undecane, 5-methyl-	Hydrocarbons	C12H26	1.345	0.016
14	AMW1743*107	2,6,8-trimethyl-Decane	Hydrocarbons	C13H28	1.379	0.000
15	AMW2559*236	Phenol, 3,4-dimethyl-	Benzene and substituted derivatives	C8H10O	1.214	0.003
16	AMW2512*359	1H-3a,7-Methanoazulene, 2,3,4,7,8,8a-hexahydro-3,6,8,8-tetramethyl-, [3R-(3.alpha.,3a.beta.,7.beta.,8a.alpha.)]-	Terpenoids	C15H24	1.501	0.001
17	AMW1739*068	2,2,6-trimethyl-Octane	Hydrocarbons	C11H24	1.194	0.000
18	AMW2814*366	Dodecane, 6-methyl-	Hydrocarbons	C13H28	1.379	0.013
19	AMW1740*372	2,3,6-trimethyl-Octane	Hydrocarbons	C11H24	1.320	0.000
20	AMW0571*326	3-Carene	Terpenoids	C10H16	1.084	0.036
21	AMW1190*248	1-(6-methyl-3-pyridinyl)-Ethanone	Heterocyclic compounds	C8H9NO	1.182	0.027
22	AMW0001*117	2,8,8-trimethyl-Decane	Hydrocarbons	C13H28	1.462	0.003
23	AMW1629*335	3-methyl-Nonane	Hydrocarbons	C10H22	1.303	0.000
24	AMW1703*122	4-methyl-4-Undecene	Hydrocarbons	C12H24	1.433	0.002

25	AMW2736*330	Cyclooctane, 1,4-dimethyl-, trans-	Terpenoids	C10H20	1.330	0.021
26	AMW4452*118	2-Nonen-1-ol, (E)-	Alcohol and amines	C9H18O	1.349	0.000
27	AMW3031*248	5-Acetyl-2-methylpyridine	Heterocyclic compounds	C8H9NO	1.182	0.027
28	AMW2349*351	Pentanoic acid, 2-methylbutyl ester	Aldehyde, Ketones, Esters	C10H20O2	1.272	0.032
29	AMW0771*368	Decane, 3-ethyl-3-methyl-	Hydrocarbons	C13H28	1.382	0.012
30	AMW0862*111	3-Cyclohexen-1-ol, 4-methyl-1-(1-methylethyl)-, (R)-	Terpenoids	C10H18O	1.390	0.000
31	AMW0777*369	Undecane, 4,6-dimethyl-	Hydrocarbons	C13H28	1.298	0.001
32	AMW0772*369	Undecane, 4,4-dimethyl-	Hydrocarbons	C13H28	1.298	0.001
33	AMW3081*352	n-Amyl isovalerate	Aldehyde, Ketones, Esters	C10H20O2	1.394	0.000
34	AMW4936*233	Maleic acid	Organic acid and Its derivatives	C4H4O4	1.357	0.000
35	AMW2965*207	1H-1,2,4-Triazol-3-amine, 5-methyl-	Alcohol and amines	C3H6N4	1.279	0.002
36	AMW3412*184	Fomepizole	Heterocyclic compounds	C4H6N2	1.257	0.000
37	AMW0761*074	Nonane, 4,5-dimethyl-	Hydrocarbons	C11H24	1.501	0.000
38	AMW0177*317	.alpha.-Muurolene	Terpenoids	C15H24	1.291	0.010
39	AMW2488*252	2-Methoxy-5-methylphenol	Benzene and substituted derivatives	C8H10O2	1.369	0.017
40	AMW4939*283	5-Tridecene, (E)-	Hydrocarbons	C13H26	1.323	0.001
41	AMW1242*255	2,7-dimethyl-1,6-Octadiene	Hydrocarbons	C10H18	1.168	0.030
42	AMW4834*270	3-Cyclohexen-1-ol, 1-methyl-4-(1-methylethyl)-	Terpenoids	C10H18O	1.242	0.041
43	AMW1236*269	(E)-2-Decenal	Aldehyde, Ketones, Esters	C10H18O	1.304	0.030
44	AMW0660*272	2,7-dimethyl-1-Octanol	Alcohol and amines	C10H22O	1.259	0.001
45	AMW3055*386	1-Heptadecene	Hydrocarbons	C17H34	1.220	0.037
46	AMW0283*132	3,5-Dimethyldodecane	Hydrocarbons	C14H30	1.290	0.001
47	AMW0762*074	Nonane, 2,5-dimethyl-	Hydrocarbons	C11H24	1.501	0.000
48	AMW2201*257	Naphthalene, 2-methyl-	Benzene and substituted derivatives	C11H10	1.331	0.035
49	AMW1665*266	Cyclohexanone, 2-methyl-5-(1-methylethenyl)-, trans-	Terpenoids	C10H16O	1.291	0.003
50	AMW2378*293	Tridecane, 4,8-dimethyl-	Hydrocarbons	C15H32	1.271	0.002

51	AMW3359*352	Butanoic acid, 3-methyl-, 3-methylbutyl ester	Aldehyde, Ketones, Esters	C10H20O2	1.394	0.000
52	AMW0971*151	Z-11-Hexadecenoic acid	Organic acid and Its derivatives	C16H30O2	1.173	0.004
53	AMW0215*095	1,4-diethenyl-Benzene	Benzene and substituted derivatives	C10H10	1.233	0.003
54	AMW2893*368	Undecane, 3-ethyl-	Hydrocarbons	C13H28	1.382	0.012
55	AMW2627*259	3-Vinyl-1,2-dithiacyclohex-4-ene	Heterocyclic compounds	C6H8S2	1.370	0.006
56	AMW1002*222	Ethanone, 1-(1H-pyrazol-4-yl)-	Aldehyde, Ketones, Esters	C5H6N2O	1.415	0.035
57	AMW0581*161	1H-Cyclopenta[1,3]cyclopropa[1,2]benzene, octahydro-7-methyl-3-methylene-4-(1-methylethyl)-, [3aS-(3a.alpha.,3b.beta.,4.beta.,7.alpha.,7aS*)]-	Terpenoids	C15H24	1.512	0.001
58	AMW2724*337	3-Cyclohexen-1-one, 2-isopropyl-5-methyl-	Terpenoids	C10H16O	1.507	0.000
59	AMW2585	L-Fenchone	Terpenoids	C10H16O	1.090	0.046
60	AMW0396	2-Decanol	Alcohol and amines	C10H22O	1.382	0.006
61	AMW0785	1,4-Dihydro-4-oxopyridazine	Heterocyclic compounds	C4H4N2O	1.047	0.035
62	AMW1936	(Z)-3-Octenoic acid, methyl ester	Aldehyde, Ketones, Esters	C9H16O2	1.233	0.042
63	AMW2257	Aminothiazole	Heterocyclic compounds	C3H4N2S	1.415	0.027
64	AMW4876	2-Heptenal, 2-propyl-	Aldehyde, Ketones, Esters	C10H18O	1.176	0.001
65	AMW2670	Benzaldehyde, 3,4-dimethyl-	Aldehyde, Ketones, Esters	C9H10O	1.295	0.008
66	AMW2445	Decane, 2,5-dimethyl-	Hydrocarbons	C12H26	1.235	0.045
67	AMW3405	2-Furanmethanol, tetrahydro-, acetate	Aldehyde, Ketones, Esters	C7H12O3	1.076	0.010
68	AMW5049	Benzene, 1,2-dimethoxy-4-(1-propenyl)-	Benzene and substituted derivatives	C11H14O2	1.112	0.026
69	AMW4913	3-Acetamidofuran	Heterocyclic compounds	C6H7NO2	1.510	0.001
70	AMW1245	3,4-Dimethyl-3-pyrrolin-2-one	Aldehyde, Ketones, Esters	C6H9NO	1.510	0.000
71	AMW3727	Hydroxylamine, O-(phenylmethyl)-	Alcohol and amines	C7H9NO	1.285	0.023
72	AMW2779	4(3H)-Pyrimidinone, 3-methyl-	Aldehyde, Ketones, Esters	C5H6N2O	1.343	0.025
73	AMW3329	2,5-Dihydroxy-4-methoxyacetophenone	Aldehyde, Ketones, Esters	C9H10O4	1.290	0.031
74	AMW2841	1,4-Dioxane-2,6-dione	Aldehyde, Ketones, Esters	C4H4O4	1.421	0.020
75	AMW0022	(E)-2-Butenoic acid, 2-(methylenecyclopropyl)prop-2-yl ester	Aldehyde, Ketones, Esters	C11H16O2	1.497	0.001

76	AMW2344	N-(1-Cyano-1-methylethyl)isobutyramide	Alcohol and amines	C8H14N2O	1.511	0.001
77	AMW0446	Paramethadione	Aldehyde, Ketones, Esters	C7H11NO3	1.236	0.000
78	AMW0026	5-Ethoxy-2-ethoxymethyl-3,4-dihydro-2H-pyrrole	Heterocyclic compounds	C9H17NO2	1.464	0.002
79	AMW2246	3,6-dimethyl-1,4-Dioxane-2,5-dione	Aldehyde, Ketones, Esters	C6H8O4	1.306	0.038
80	AMW3301	5-Nonanol	Alcohol and amines	C9H20O	1.162	0.019
81	AMW3942	N-Methyl-2-isopropoxycarbonylazetidine	Aldehyde, Ketones, Esters	C8H15NO2	1.440	0.000
82	AMW0562	6-methyl-3(2H)-Pyridazinone	Aldehyde, Ketones, Esters	C5H6N2O	1.488	0.001
83	AMW0654	1,3-dimethoxy-Benzene	Ethers	C8H10O2	1.393	0.011
84	AMW4371	Cyclohexanamine, N-hydroxy-	Alcohol and amines	C6H13NO	1.017	0.046
85	AMW3875	Butan-2-ol, 3,3-dimethyl-1-(pyrrolidin-1-yl)-	Alcohol and amines	C10H21NO	1.472	0.002
86	AMW4319	4,4'-Bitriazolyl	Heterocyclic compounds	C4H4N6	1.285	0.034
87	AMW0887	6-Nonynoic acid, methyl ester	Aldehyde, Ketones, Esters	C10H16O2	1.457	0.001
88	AMW1414	5-methyl-2-(1-methylethyl)-2-Cyclohexen-1-one	Aldehyde, Ketones, Esters	C10H16O	1.411	0.003
89	AMW0765	2,3-Dimethyldecane	Hydrocarbons	C12H26	1.473	0.002
90	AMW1264	2-phenoxy-1-Propanol	Alcohol and amines	C9H12O2	1.475	0.001
91	AMW2343	3,4-Dimethoxytoluene	Ethers	C9H12O2	1.436	0.005
92	AMW3838	3H-Pyrazole, 3,4-diamino-	Heterocyclic compounds	C3H6N4	1.404	0.009
93	AMW1794	Hexanoic acid, butyl ester	Aldehyde, Ketones, Esters	C10H20O2	1.100	0.025
94	AMW0192	propyl-Benzene	Benzene and substituted derivatives	C9H12	1.562	0.000
95	AMW4870	4-Acetylbutyric acid	Organic acid and Its derivatives	C6H10O3	1.443	0.004
96	AMW1384	4-hydroxybenzenepropanoic acid	Organic acid and Its derivatives	C9H10O3	1.302	0.028
97	AMW3268	2,4-Dimethylsulfolane	Heterocyclic compounds	C6H12O2S	1.351	0.019
98	AMW1506	2,5-Dihydroxy-4-isopropyl-2,4,6-cycloheptatrien-1-one	Aldehyde, Ketones, Esters	C10H12O3	1.316	0.027
99	AMW3722	(S)-(-)-2-(Methoxymethyl)-1-pyrrolidinecarboxaldehyde	Aldehyde, Ketones, Esters	C7H13NO2	1.363	0.002
100	AMW1748	trans-4-tert-butylcycloheptanol	Alcohol and amines	C11H22O	1.288	0.027
101	AMW3146	2-tert-Butoxytetrahydrofuran	Heterocyclic compounds	C8H16O2	1.235	0.027

102	AMW4465	N-Cyclopropanecarbonylcyclopropanecarbohydrazide	Nitrogen compounds	C8H12N2O2	1.270	0.001
103	AMW2934	2-Amino-2-oxoethyl acetate	Aldehyde, Ketones, Esters	C4H7NO3	1.422	0.005
104	AMW2714	Dodecyl isobutyl ether	Ethers	C16H34O	1.328	0.011
105	AMW3220	Octane, 2-iodo-	Halogenated hydrocarbons	C8H17I	1.369	0.000
106	AMW1383	Phenol, 4-(2-propenyl)-	Benzene and substituted derivatives	C9H10O	1.343	0.023
107	AMW2652	2-Cyclopenten-1-one, 2-hydroxy-3-methyl-	Aldehyde, Ketones, Esters	C6H8O2	1.380	0.001
108	AMW4774	Thiophene, 2-(methylthio)-	Heterocyclic compounds	C5H6S2	1.260	0.001
109	AMW1195	Furaneol	Aldehyde, Ketones, Esters	C6H8O3	1.477	0.000
110	AMW0228	Caprolactam	Alcohol and amines	C6H11NO	1.282	0.024
111	AMW3948	Carbonic acid, neopentyl 2-ethylhexyl ester	Aldehyde, Ketones, Esters	C14H28O3	1.396	0.009
112	AMW3944	2,2-Dimethylpropionic acid, 4-methylpentyl ester	Aldehyde, Ketones, Esters	C11H22O2	1.411	0.000
113	AMW1572	2-methyl-2-Undecene	Hydrocarbons	C12H24	1.264	0.029
114	AMW0410	isothiocyanato-Cyclohexane	Nitrogen compounds	C7H11NS	1.356	0.003
115	AMW2681	4-Methyleneisophorone	Aldehyde, Ketones, Esters	C10H14O	1.389	0.012
116	AMW4336	Nitric acid, octyl ester	Aldehyde, Ketones, Esters	C8H17NO3	1.398	0.005
117	AMW0148	4-Amino-4,5(1H)-dihydro-1,2,4-triazole-5-one	Aldehyde, Ketones, Esters	C2H4N4O	1.467	0.002
118	AMW4035	N-(2-Cyano-1-methylvinyl)acetamide	Alcohol and amines	C6H8N2O	1.121	0.049
119	AMW0712	1,9-Decadiene	Hydrocarbons	C10H18	1.344	0.000
120	AMW0405	2-Undecanone	Aldehyde, Ketones, Esters	C11H22O	1.513	0.000
121	AMW2633	2-Undecene, 3-methyl-, (E)-	Hydrocarbons	C12H24	1.281	0.030
122	AMW4694	3,4-Diethyl-3-hexene	Hydrocarbons	C10H20	1.125	0.005
123	AMW0303	m-Chloroaniline	Alcohol and amines	C6H6ClN	1.424	0.005
124	AMW3454	(4aS,9aR)-3,5,5,9-Tetramethyl-2,4a,5,6,7,9a-hexahydro-1H-benzo[7]annulene	Heterocyclic compounds	C15H24	1.435	0.004
125	AMW1948	Undecane, 2-methyl-	Hydrocarbons	C12H26	1.387	0.011
126	AMW1667	1,3-Dihydro-2H-indol-2-one	Aldehyde, Ketones, Esters	C8H7NO	1.341	0.013

127	AMW1841	N,N-diethyl-Urea	Nitrogen compounds	C5H12N2O	1.415	0.007
128	AMW3934	1H,5H,7H,11H-Dipyrazolo[1,2-a:1',2'-d][1,2,4,5]tetrazine, tetrahydro-	Heterocyclic compounds	C8H16N4	1.329	0.002
129	AMW0180	3-Ethylcyclopentanone	Aldehyde, Ketones, Esters	C7H12O	1.264	0.000
130	AMW3138	5-Hydroxy-2-pyrimidinecarbonitrile	Nitrogen compounds	C5H3N3O	1.473	0.000
131	AMW4328	8-Azabicyclo[3.2.1]octan-3-ol, 8-methyl-, endo-	Alcohol and amines	C8H15NO	1.422	0.006
132	AMW0135	N-(2-Pyridinylmethyl)-1-butanamine, N-acetyl-	Alcohol and amines	C12H18N2O	1.330	0.015
133	AMW4506	Dimethyl-1,2,3-oxadiazol-3-one	Aldehyde, Ketones, Esters	C4H6N2O2	1.376	0.012
134	AMW3695	6-Undecanol	Alcohol and amines	C11H24O	1.332	0.000
135	AMW2579	Propanoic acid, 2-hydroxyethyl ester	Aldehyde, Ketones, Esters	C5H10O3	1.550	0.000
136	AMW0749	Naphthalene, decahydro-4a-methyl-1-methylene-7-(1-methylethenyl)-, [4aR-(4a.alpha.,7.alpha.,8a.beta.)]-	Terpenoids	C15H24	1.457	0.003
137	AMW2576	Dodecane, 5-methyl-	Hydrocarbons	C13H28	1.171	0.034
138	AMW3919	1,4-Dimethyl-2-piperidone	Aldehyde, Ketones, Esters	C7H13NO	1.498	0.001
139	AMW3485	Acetonitrile, 2,2'-iminobis-	Nitrogen compounds	C4H5N3	1.269	0.040
140	AMW3007	Oxalic acid, butyl cyclobutyl ester	Aldehyde, Ketones, Esters	C10H16O4	1.273	0.002
141	AMW1578	3,4-Dimethylcyclohexanol	Alcohol and amines	C8H16O	1.462	0.000
142	AMW0409	nitro-Cyclohexane	Nitrogen compounds	C6H11NO2	1.296	0.043
143	AMW0555	4-methyl-1-Decene	Hydrocarbons	C11H22	1.392	0.008
144	AMW0622	2,2,4-trimethyl-1,3-Pentanediol	Alcohol and amines	C8H18O2	1.315	0.024
145	AMW2068	N,N-dibutyl-Formamide	Alcohol and amines	C9H19NO	1.269	0.044
146	AMW1302	2-Phenylpropenal	Aldehyde, Ketones, Esters	C9H8O	1.235	0.000
147	AMW1235	2-butyl-1-Octanol	Alcohol and amines	C12H26O	1.373	0.000
148	AMW1063	2-Methyl-3-furanthiol	Alcohol and amines	C5H6OS	1.490	0.000
149	AMW4866	Pyrrolidine, 1-acetyl-	Heterocyclic compounds	C6H11NO	1.341	0.019
150	AMW0064	Oxalic acid, neopentyl propyl ester	Aldehyde, Ketones, Esters	C10H18O4	1.238	0.028
151	AMW4708	2-Isopropylpyrazine	Heterocyclic compounds	C7H10N2	1.169	0.001

152	AMW2341	Dodecane, 3-methyl-	Hydrocarbons	C13H28	1.385	0.000
153	AMW1114	Dodecane, 2,6,11-trimethyl-	Hydrocarbons	C15H32	1.378	0.000
154	AMW2803	Pentanoic acid, 2-methyl-, anhydride	Organic acid and Its derivatives	C12H22O3	1.355	0.001
155	AMW4922	8-Hydroxy-2-octanone	Aldehyde, Ketones, Esters	C8H16O2	1.277	0.041
156	AMW4691	1-Pentanol, 4-amino-	Alcohol and amines	C5H13NO	1.397	0.000
157	AMW1001	1,3-Dimethyl-1H-pyrazole-4-carbaldehyde	Aldehyde, Ketones, Esters	C6H8N2O	1.384	0.010
158	AMW3990	Carbonic acid, monoamide, N-(2-pentyl)-N-butyl-, propargyl ester	Aldehyde, Ketones, Esters	C13H23NO2	1.407	0.000
159	AMW0500	Diphenylamine	Alcohol and amines	C12H11N	1.379	0.013
160	AMW0774	Nonane, 5-methyl-5-propyl-	Hydrocarbons	C13H28	1.319	0.015
161	AMW3142	Nonane, 5-propyl-	Hydrocarbons	C12H26	1.344	0.000
162	AMW2853	Hexahydro-1,3,5-trinitroso-1,3,5-triazine	Heterocyclic compounds	C3H6N6O3	1.347	0.000
163	AMW3533	Bicyclo(3.1.1)heptane-2,3-diol, 2,6,6-trimethyl-	Alcohol and amines	C10H18O2	1.343	0.001
164	AMW1225	4-Amino-2(1H)-pyridinone	Aldehyde, Ketones, Esters	C5H6N2O	1.075	0.030
165	AMW4377	l-Alanine, n-propargyloxycarbonyl-, pentyl ester	Aldehyde, Ketones, Esters	C12H19NO4	1.171	0.015
166	AMW3776	4H-1,2,4-Triazole, 4-ethyl-	Heterocyclic compounds	C4H7N3	1.382	0.001
167	AMW0277	Phenylglyoxal	Aldehyde, Ketones, Esters	C8H6O2	1.309	0.000
168	AMW3876	Oxirane, dodecyl-	Heterocyclic compounds	C14H28O	1.402	0.007
169	AMW4267	Vinyl 2-ethylhexanoate	Aldehyde, Ketones, Esters	C10H18O2	1.237	0.003
170	AMW3014	Acetic acid, [(1,1-dimethylethyl)thio]-	Organic acid and Its derivatives	C6H12O2S	1.333	0.000
171	AMW2364	4-Methylpentyl 4-methylpentanoate	Aldehyde, Ketones, Esters	C12H24O2	1.470	0.000
172	AMW5110	1-Tetradecanamine	Alcohol and amines	C14H31N	1.357	0.008
173	AMW4131	2,2-Dimethyl-3-methoxy-cyclopropane-1-carboxylic acid	Organic acid and Its derivatives	C7H12O3	1.012	0.002
174	AMW4111	Pyrazole-4-carboxaldehyde, 1-ethyl-5-methyl-	Aldehyde, Ketones, Esters	C7H10N2O	1.512	0.000
175	AMW4941	3-Hexanol, 1,5-dimethoxy-2,4-dimethyl-	Alcohol and amines	C10H22O3	1.259	0.000

176	AMW3996	(2S,4aS,5R,8aR)-2,5-Dipropyldecahydroquinoline	Heterocyclic compounds	C15H29N	1.344	0.002
177	AMW4469	Hexyl 4-methoxyphenyl ether	Ethers	C13H20O2	1.306	0.004
178	AMW3622	Cyclohexanemethanol, 4-methyl-, cis-	Alcohol and amines	C8H16O	1.034	0.011
179	AMW2260	4-t-Butylcyclohexathiazole	Heterocyclic compounds	C11H17NS	1.439	0.000
180	AMW3804	1-(1-Cyclopropyl-pentyl)piperidine	Heterocyclic compounds	C13H25N	1.422	0.000
181	AMW3281	Oxalic acid, allyl ethyl ester	Aldehyde, Ketones, Esters	C7H10O4	1.354	0.004
182	AMW2429	2H-Pyran-2-one, tetrahydro-6-propyl-	Aldehyde, Ketones, Esters	C8H14O2	1.356	0.000
183	AMW1489	1-Nonene, 4,6,8-trimethyl-	Hydrocarbons	C12H24	1.455	0.001
184	AMW4763	3(2H)-Furanone, 4-hydroxy-5-methyl-	Aldehyde, Ketones, Esters	C5H6O3	1.430	0.002
185	AMW3997	4-Methylpiperidine-2-carboxylic acid, methyl ester	Aldehyde, Ketones, Esters	C8H15NO2	1.357	0.000
186	AMW0946	Nonaneperoxoic acid, 1,1-dimethylethyl ester	Aldehyde, Ketones, Esters	C13H26O3	1.290	0.012
187	AMW2589	Pentanoic acid, 2-methylpropyl ester	Aldehyde, Ketones, Esters	C9H18O2	1.464	0.000
188	AMW3768	Urea, 2-propenyl-	Nitrogen compounds	C4H8N2O	1.269	0.031
189	AMW1931	2(3H)-Furanone, 5-ethyldihydro-	Aldehyde, Ketones, Esters	C6H10O2	1.449	0.002
190	AMW0159	Benzaldehyde	Aldehyde, Ketones, Esters	C7H6O	1.565	0.000
191	AMW5097	1-Naphthalenol, 1,2,3,4-tetrahydro-2,5,8-trimethyl-	Benzene and substituted derivatives	C13H18O	1.358	0.010
192	AMW3500	Precocene I	Heterocyclic compounds	C12H14O2	1.476	0.000
193	AMW4949	Phenol, p-tert-butyl-	Benzene and substituted derivatives	C10H14O	1.371	0.001
194	AMW2898	3,5-Dimethylpyrazole-1-carboxamide	Alcohol and amines	C6H9N3O	1.462	0.000
195	AMW0734	3,5-Heptanedione, 4-ethyl-2,2,6,6-tetramethyl-	Aldehyde, Ketones, Esters	C13H24O2	1.367	0.021
196	AMW2623	6,8-Dioxabicyclo[3.2.1]octane, 7-ethyl-5-methyl-, (1R-exo)-	Heterocyclic compounds	C9H16O2	1.373	0.012
197	AMW0062	Oxalic acid, ethyl 2-isopropylphenyl ester	Aldehyde, Ketones, Esters	C13H16O4	1.053	0.031
198	AMW4895	4H-Pyran-4-one, 3,5-dihydroxy-2-methyl-	Aldehyde, Ketones, Esters	C6H6O4	1.303	0.001
199	AMW0102	Ethyl 3-methylbut-3-enyl carbonate	Aldehyde, Ketones, Esters	C8H14O3	1.328	0.000
200	AMW4405	Tetradecane, 2,5-dimethyl-	Hydrocarbons	C16H34	1.266	0.003
201	AMW3715	1-Methyl-2,4,5-trioxoimidazolidine	Heterocyclic compounds	C4H4N2O3	1.310	0.001

202	AMW0911	cis-7-decenal	Aldehyde, Ketones, Esters	C10H18O	1.234	0.003
203	AMW1693	2,3-dichloro-Benzenamine	Alcohol and amines	C6H5Cl2N	1.194	0.000
204	AMW1603	Cyclohexene, 1-methyl-4-(1-methylethylidene)-	Terpenoids	C10H16	1.436	0.004
205	AMW4749	1,3-Dithiane	Heterocyclic compounds	C4H8S2	1.039	0.012
206	AMW3148	2-Cyano-2-isopropyl-3-methylbutanoic acid	Organic acid and Its derivatives	C9H15NO2	1.331	0.000
207	AMW2157	1,8-Ethylenenaphthalene	Benzene and substituted derivatives	C12H10	1.430	0.000
208	AMW3216	1H-Pyrrole, 3-ethyl-2,4,5-trimethyl-	Heterocyclic compounds	C9H15N	1.148	0.011
209	AMW3347	Decane, 3-bromo-	Halogenated hydrocarbons	C10H21Br	1.291	0.000
210	AMW4363	4-Amino-3-hydroxytetrahydrothiophene 1,1-dioxide	Heterocyclic compounds	C4H9NO3S	1.427	0.005
211	AMW3091	2,4-Imidazolidinedione, 5,5-dimethyl-	Aldehyde, Ketones, Esters	C5H8N2O2	1.351	0.000
212	AMW4049	(3R)-(+)-3-Acetamidopyrrolidine	Heterocyclic compounds	C6H12N2O	1.422	0.001
213	AMW3041	5-Methylenehydantoin	Nitrogen compounds	C4H4N2O2	1.349	0.000
214	AMW2323	n-Caprylic acid isobutyl ester	Aldehyde, Ketones, Esters	C12H24O2	1.325	0.001
215	AMW3675	Valeric anhydride	Organic acid and Its derivatives	C10H18O3	1.343	0.005
216	AMW3378	Heptane, 4-ethyl-	Hydrocarbons	C9H20	1.487	0.000
217	AMW2938	Tridecane, 7-methyl-	Hydrocarbons	C14H30	1.330	0.001
218	AMW1184	4(1H)-Pyridinone, 2,3-dihydro-1-methyl-	Aldehyde, Ketones, Esters	C6H9NO	1.415	0.000
219	AMW3964	l-Alanine, N-ethoxycarbonyl-, hexyl ester	Aldehyde, Ketones, Esters	C12H23NO4	1.300	0.007
220	AMW1669	2-Propanol, 1,3-bis(dimethylamino)-	Alcohol and amines	C7H18N2O	1.084	0.007
221	AMW5050	1,1'-Biphenyl, 4-methyl-	Benzene and substituted derivatives	C13H12	1.488	0.001
222	AMW3499	(Z)-(Z)-Hex-3-en-1-yl 2-methylbut-2-enoate	Aldehyde, Ketones, Esters	C11H18O2	1.223	0.009
223	AMW4125	Butanedioic acid, 2-hydroxy-2-methyl-, (S)-	Organic acid	C5H8O5	1.316	0.001
224	AMW0717	2-Undecanol	Alcohol and amines	C11H24O	1.283	0.005
225	AMW4160	Dodecane, 2,5-dimethyl-	Hydrocarbons	C14H30	1.282	0.003
226	AMW5118	Furazandiamine	Alcohol and amines	C2H4N4O	1.401	0.000

227	AMW1291	Nonane, 2,2,4,4,6,8,8-heptamethyl-	Hydrocarbons	C16H34	1.255	0.004
228	AMW0065	Oxalic acid, isobutyl neopentyl ester	Aldehyde, Ketones, Esters	C11H20O4	1.474	0.000
229	AMW3860	2(5H)-Furanone, 5-(1-methylethyl)-	Aldehyde, Ketones, Esters	C7H10O2	1.246	0.016
230	AMW2786	3-Methylpyridazine	Heterocyclic compounds	C5H6N2	1.164	0.014
231	AMW0994	Cyclopentaneacetic acid, 3-oxo-2-pentyl-, methyl ester	Aldehyde, Ketones, Esters	C13H22O3	1.453	0.002
232	AMW4068	Creatinine	Organic acid and Its derivatives	C4H7N3O	1.362	0.000
233	AMW4461	Phenol, 2-amino-4-methoxy-	Benzene and substituted derivatives	C7H9NO2	1.034	0.038
234	AMW1829	Octane, 1,1'-oxybis-	Ethers	C16H34O	1.347	0.005
235	AMW1579	Benzenemethanol, .alpha.-2-cyclohexen-1-yl-	Alcohol and amines	C13H16O	1.041	0.000
236	AMW1823	Pentadecane	Hydrocarbons	C15H32	1.407	0.000
237	AMW2902	1H-1,2,3-Triazole-4-carboxaldehyde	Aldehyde, Ketones, Esters	C3H3N3O	1.261	0.000
238	AMW2517	exo-2-Hydroxycineole acetate	Aldehyde, Ketones, Esters	C12H20O3	1.141	0.008
239	AMW0821	(Z)-6-dodecen-γ-lactone	Aldehyde, Ketones, Esters	C12H20O2	1.416	0.003
240	AMW0154	2,4,6-Pyrimidinetriamine	Alcohol and amines	C4H7N5	1.281	0.000
241	AMW3303	Decane, 2,3,5,8-tetramethyl-	Hydrocarbons	C14H30	1.390	0.000
242	AMW0490	2-Imidazolidinone	Aldehyde, Ketones, Esters	C3H6N2O	1.279	0.001
243	AMW2120	(2,2,6-Trimethyl-bicyclo[4.1.0]hept-1-yl)-methanol	Alcohol and amines	C11H20O	1.288	0.001
244	AMW3547	Ethanediamide	Alcohol and amines	C2H4N2O2	1.181	0.009
245	AMW1462	3-ethyl-Pyridine	Heterocyclic compounds	C7H9N	1.555	0.000
246	AMW3473	1,3,5-Triazine-2,4,6-triamine	Heterocyclic compounds	C3H6N6	1.035	0.050
247	AMW2777	2,4-Dimethyldodecane	Hydrocarbons	C14H30	1.410	0.001
248	AMW0819	N-(n-Butoxymethyl)acrylamide	Alcohol and amines	C8H15NO2	1.324	0.000
249	AMW3323	Dodecane, 1-methoxy-	Ethers	C13H28O	1.226	0.031
250	AMW3154	2,5-Dimethoxy-4-ethylamphetamine	Alcohol and amines	C13H21NO 2	1.170	0.036
251	AMW3629	Ethanone, 1-(1-methylcyclohexyl)-	Aldehyde, Ketones, Esters	C9H16O	1.349	0.000

252	AMW3982	2H-Pyran-2-one, 3-acetyl-4-hydroxy-6-methyl-	Organic acid and Its derivatives	C8H8O4	1.417	0.001
253	AMW4438	Ethanol, 2,2'-oxybis-, dipropanoate	Aldehyde, Ketones, Esters	C10H18O5	1.397	0.000
254	AMW3403	1H-1,2,3,4-Tetrazole, 5-hydrazino-	Nitrogen compounds	CH4N6	1.309	0.001
255	AMW0066	Oxalic acid, hexyl neopentyl ester	Aldehyde, Ketones, Esters	C13H24O4	1.326	0.001
256	AMW3214	4-Heptanone, 2,2,3,3,5,5,6,6-octamethyl-	Aldehyde, Ketones, Esters	C15H30O	1.250	0.001
257	AMW2389	Propyl octanoate	Aldehyde, Ketones, Esters	C11H22O2	1.468	0.000
258	AMW0103	Ethyl 2-(5-methyl-5-vinyltetrahydrofuran-2-yl)propan-2-yl carbonate	Aldehyde, Ketones, Esters	C13H22O4	1.269	0.003
259	AMW4976	6-Dodecanone	Aldehyde, Ketones, Esters	C12H24O	1.281	0.001
260	AMW3694	2-Hexyl-5-pentylpyrrolidine	Heterocyclic compounds	C15H31N	1.377	0.001
261	AMW5072	3-Hydroxy-4-methoxybenzoic acid	Organic acid and Its derivatives	C8H8O4	1.349	0.000
262	AMW0451	4-[pyrrolidin-2-one-5-yl]-Butan-2-one	Aldehyde, Ketones, Esters	C8H13NO2	1.169	0.007
263	AMW3439	Hydratropic acid, isopropyl ester	Aldehyde, Ketones, Esters	C12H16O2	1.228	0.000
264	AMW2413	Tridecane, 5-methyl-	Hydrocarbons	C14H30	1.314	0.000
265	AMW0476	Benzophenone	Aldehyde, Ketones, Esters	C13H10O	1.371	0.008
266	AMW5037	3-Hydroxynonanoic acid	Organic acid and Its derivatives	C9H18O3	1.268	0.014
267	AMW0783	1H-Imidazole-2-methanol, 1-methyl-	Alcohol and amines	C5H8N2O	1.341	0.000
268	AMW1412	Sarcosine anhydride	Aldehyde, Ketones, Esters	C6H10N2O2	1.172	0.000
269	AMW1273	2-methoxy-6-methyl-4H-Pyran-4-one	Aldehyde, Ketones, Esters	C7H8O3	1.473	0.000
270	AMW4836	.alpha.-Santoline alcohol	Alcohol and amines	C10H18O	1.195	0.027
271	AMW4105	2',4'-Dihydroxy-3'-methylacetophenone	Aldehyde, Ketones, Esters	C9H10O3	1.029	0.000
272	AMW5108	8-Pentadecanone	Aldehyde, Ketones, Esters	C15H30O	1.307	0.002
273	AMW2084	Vinyl lauryl ether	Ethers	C14H28O	1.233	0.010
274	AMW3039	3,6-Dimethylpiperazine-2,5-dione	Aldehyde, Ketones, Esters	C6H10N2O2	1.223	0.005
275	AMW3755	dl-2-Aminobutyric acid	Organic acid and Its derivatives	C4H9NO2	1.544	0.000

276	AMW3511	Lacthydrazide	Nitrogen compounds	C3H8N2O2	1.421	0.003
277	AMW1292	9-Azabicyclo[3.3.1]nonan-3-one	Aldehyde, Ketones, Esters	C8H13NO	1.200	0.015
278	AMW5116	2(3H)-Benzothiazolone	Aldehyde, Ketones, Esters	C7H5NOS	1.304	0.012
279	AMW2027	(E)-2-Tridecen-1-ol	Hydrocarbons	C13H26O	1.310	0.001
280	AMW3396	Acetamide, N-(aminocarbonyl)-	Alcohol and amines	C3H6N2O2	1.238	0.004
281	AMW0559	2-Bromo dodecane	Halogenated hydrocarbons	C12H25Br	1.236	0.019
282	AMW3812	Acetoxyacetic acid, 4-cyanophenyl ester	Aldehyde, Ketones, Esters	C11H9NO4	1.250	0.002
283	AMW3898	Thiophene-2-acetic acid, 2-dimethylaminoethyl ester	Aldehyde, Ketones, Esters	C10H15NO2S	1.206	0.004
284	AMW0912	2,6-Dodecadien-1-al	Aldehyde, Ketones, Esters	C12H20O	1.201	0.000
285	AMW3149	Diethanolamine	Alcohol and amines	C4H11NO2	1.410	0.000
286	AMW3406	Isobutyl 2-(4-methylcyclohex-3-enyl)propan-2-yl carbonate	Aldehyde, Ketones, Esters	C15H26O3	1.249	0.000
287	AMW2588	Pentadecane, 2-methyl-	Hydrocarbons	C16H34	1.187	0.021
288	AMW3773	3-Isopropyl-6,10-dimethylundecane-2-ol	Alcohol and amines	C16H34O	1.406	0.022
289	AMW3746	Sulfurous acid, isobutyl pentyl ester	Aldehyde, Ketones, Esters	C9H20O3S	1.279	0.002
290	AMW2272	5-Ethyl-5-(propan-2-yl)imidazolidine-2,4-dione	Aldehyde, Ketones, Esters	C8H14N2O2	1.288	0.001
291	AMW2315	Heptylcyclohexane	Hydrocarbons	C13H26	1.014	0.008
292	AMW4345	Dichloroacetic acid, 2-tetrahydrofurylmethyl ester	Aldehyde, Ketones, Esters	C7H10Cl2O3	1.241	0.041
293	AMW2443	1H-Inden-5-ol, 2,3-dihydro-	Benzene and substituted derivatives	C9H10O	1.356	0.004
294	AMW2920	Pentadecane, 7-methyl-	Hydrocarbons	C16H34	1.290	0.001
295	AMW0055	Sulfurous acid, 2-ethylhexyl isobutyl ester	Organic acid and Its derivatives	C12H26O3S	1.226	0.004
296	AMW5069	2,2-Dimethylpropionic acid, decyl ester	Aldehyde, Ketones, Esters	C15H30O2	1.253	0.001
297	AMW1556	1-Dodecen-1-ol, acetate	Aldehyde, Ketones, Esters	C14H26O2	1.143	0.000
298	AMW3938	Heptyl ethylphosphonofluoridate	Aldehyde, Ketones, Esters	C9H20FO2	1.200	0.036

				P		
299	AMW2941	Hexadecane, 7,9-dimethyl-	Hydrocarbons	C18H38	1.329	0.001
300	AMW3870	2,4,5-Trioxoimidazolidine	Heterocyclic compounds	C3H2N2O3	1.246	0.002
301	AMW1140	.delta.-Nonalactone	Aldehyde, Ketones, Esters	C9H16O2	1.138	0.006
302	AMW1822	Tetradecane	Hydrocarbons	C14H30	1.223	0.007
303	AMW0399	1-Tetradecene	Hydrocarbons	C14H28	1.208	0.005
304	AMW0394	Ethanol, 2-(2-ethoxyethoxy)-	Alcohol and amines	C6H14O3	1.390	0.001
305	AMW2401	Hydrazine, (phenylmethyl)-	Nitrogen compounds	C7H10N2	1.564	0.000
306	AMW2769	Ethyl tridecanoate	Aldehyde, Ketones, Esters	C15H30O2	1.290	0.003
307	AMW4947	1-Tridecene	Hydrocarbons	C13H26	1.489	0.000
308	AMW3765	3(2H)-Furanone, 2-(1-hydroxy-1-methyl-2-oxopropyl)-2,5-dimethyl-	Aldehyde, Ketones, Esters	C10H14O4	1.211	0.005
309	AMW4995	Taurine	Organic acid and Its derivatives	C2H7NO3S	1.229	0.002
310	AMW4996	4-Piperidinecarboxamide	Alcohol and amines	C6H12N2O	1.199	0.004
311	AMW4953	Butanedioic acid, methylene-	Organic acid and Its derivatives	C5H6O4	1.306	0.000
312	AMW0537	Tetradecanal	Aldehyde, Ketones, Esters	C14H28O	1.212	0.005
313	AMW3720	1,1,2,2-Tetrakis(allyloxy)ethane	Ethers	C14H22O4	1.443	0.001
314	AMW3574	Oxalic acid, cyclobutyl hexyl ester	Aldehyde, Ketones, Esters	C12H20O4	1.213	0.004
315	AMW5115	Benzene, decyl-	Benzene and substituted derivatives	C16H26	1.414	0.001
316	AMW3008	Propanedioic acid	Organic acid and Its derivatives	C3H4O4	1.483	0.000
317	AMW5018	Isosativene	Hydrocarbons	C15H24	1.235	0.000
318	AMW5089	1-Iodo-2-methylundecane	Halogenated hydrocarbons	C12H25I	1.204	0.004
319	AMW4442	Cyclohexanecarboxylic acid, 1-amino-	Organic acid and Its derivatives	C7H13NO2	1.232	0.004
320	AMW1088	Cyclotetradecane	Hydrocarbons	C14H28	1.261	0.004
321	AMW2363	6-Octenal, 7-methyl-3-methylene-	Terpenoids	C10H16O	1.478	0.000
322	AMW3986	N-[3-[N-Aziridyl]propylidene]tetrahydrofurfurylamine	Heterocyclic compounds	C10H18N2O	1.321	0.001

323	AMW3756	2,5-Piperazinedione, 3-methyl-	Aldehyde, Ketones, Esters	C5H8N2O2	1.225	0.003
324	AMW1294	4-(phenylmethoxy)-Benzaldehyde	Aldehyde, Ketones, Esters	C14H12O2	1.165	0.004
325	AMW3284	2-Cyanoguanidine	Nitrogen compounds	C2H4N4	1.350	0.032
326	AMW0487	(E)-Hex-3-enyl (E)-2-methylbut-2-enoate	Aldehyde, Ketones, Esters	C11H18O2	1.321	0.005
327	AMW4956	Heptane, 2-(hexyloxy)-	Ethers	C13H28O	1.384	0.000
328	AMW4337	4-Amino-3,5-dimethyl-1,2,4-triazole	Heterocyclic compounds	C4H8N4	1.260	0.003
329	AMW0582	6,7-Dodecanedione	Aldehyde, Ketones, Esters	C12H22O2	1.274	0.002
330	AMW4334	Oxalic acid, cyclohexyl ethyl ester	Aldehyde, Ketones, Esters	C10H16O4	1.262	0.003
331	AMW2873	3,5-Diamino-1,2,4-triazole	Heterocyclic compounds	C2H5N5	1.245	0.002
332	AMW1321	(1-pentylhexyl)-Benzene	Benzene and substituted derivatives	C17H28	1.157	0.000
333	AMW3174	1-Propanone, 2,2-dimethyl-1-(4-methylphenyl)-3-(1-piperidyl)-	Aldehyde, Ketones, Esters	C17H25NO	1.175	0.004
334	AMW3799	2-(Diethylaminophenylmethyl)cyclohexanol	Alcohol and amines	C17H27NO	1.188	0.003
335	AMW0600	2,6-Octadien-1-ol, 3,7-dimethyl-, acetate, (Z)-	Aldehyde, Ketones, Esters	C12H20O2	1.461	0.000
336	AMW1004	Tetradecane, 4-methyl-	Hydrocarbons	C15H32	1.096	0.000
337	AMW5081	1,6-Dioxacyclododecane-7,12-dione	Aldehyde, Ketones, Esters	C10H16O4	1.117	0.006
338	AMW3318	Oxalic acid, hexyl 2-phenylethyl ester	Aldehyde, Ketones, Esters	C16H22O4	1.186	0.003
339	AMW5017	2-Butanone, 4-(2,6,6-trimethyl-1,3-cyclohexadien-1-yl)-	Aldehyde, Ketones, Esters	C13H20O	1.080	0.001
340	AMW4815	Ethanol, 2-(hexyloxy)-	Alcohol and amines	C8H18O2	1.173	0.000
341	AMW4340	Octanoic acid, 2-tetrahydrofurylmethyl ester	Aldehyde, Ketones, Esters	C13H24O3	1.290	0.024
342	AMW0249	Tris-(hydroxymethyl)-phosphine oxide	Others	C3H9O4P	1.422	0.000
343	AMW2394	Tricyclo[2.2.1.0(2,6)]heptane, 1,7-dimethyl-7-(4-methyl-3-pentenyl)-, (-)-	Hydrocarbons	C15H24	1.274	0.018
344	AMW4209	4-Pyridinol, 3-methoxy-2-methyl-	Alcohol and amines	C7H9NO2	1.371	0.001
345	AMW1375	2,4,5-Trihydroxypyrimidine	Heterocyclic compounds	C4H4N2O3	1.188	0.003
346	AMW4216	Cyclohexane, 1,1'-(oxydi-2,1-ethanediyl)bis[4-methyl-	Ethers	C18H34O	1.275	0.016
347	AMW2774	1,2,4,5-Tetrazine, 3,6-dimethyl-	Heterocyclic compounds	C4H6N4	1.422	0.001
348	AMW4961	Bicyclo[2.2.1]heptane-2,3-dione, 1,7,7-trimethyl-, (1S)-	Aldehyde, Ketones, Esters	C10H14O2	1.316	0.000

349	AMW2867	Heptane, 2,5,5-trimethyl-	Hydrocarbons	C10H22	1.518	0.000
350	AMW3189	Ethyl mandelate	Aldehyde, Ketones, Esters	C10H12O3	1.375	0.001
351	AMW1402	4-acetyl-2,3,4,5,5-pentamethyl-2-Cyclopenten-1-one	Aldehyde, Ketones, Esters	C12H18O2	1.363	0.008
352	AMW1716	formylglycolide	Nitrogen compounds	C4H6N2O2	1.357	0.011
353	AMW3524	3,4-Diamino-1,2,4(4H)-triazole	Heterocyclic compounds	C2H5N5	1.101	0.002
354	AMW0097	5-Butyl-5-ethylheptadecane	Hydrocarbons	C23H48	1.321	0.004
355	AMW3181	Hexadecane, 5-butyl-	Hydrocarbons	C20H42	1.076	0.018
356	AMW2179	1,3-dimethyl-2,4(1H,3H)-Pyrimidinedione	Aldehyde, Ketones, Esters	C6H8N2O2	1.156	0.009
357	AMW1575	n-Hexadecanoic Acid	Organic acid and Its derivatives	C16H32O2	1.084	0.001
358	AMW0320	Cyclohexanone	Aldehyde, Ketones, Esters	C6H10O	1.539	0.000
359	AMW4075	1,7-Dimethyl-4-(1-methylethyl)cyclodecane	Hydrocarbons	C15H30	1.408	0.010
360	AMW0739	Di(n-hexyl)sulfone	Others	C12H26O2S	1.206	0.002
361	AMW2138	.alpha.-Terpinyl acetate	Aldehyde, Ketones, Esters	C12H20O2	1.550	0.000
362	AMW3711	5H-Tetrazole-5-thione, 1-[2-(dimethylamino)ethyl]-1,2-dihydro-	Alcohol and amines	C5H11N5S	1.105	0.008
363	AMW3196	Azulene, 1,4-dimethyl-7-(1-methylethyl)-	Terpenoids	C15H18	1.152	0.002
364	AMW2948	Tetradecane, 4-ethyl-	Hydrocarbons	C16H34	1.250	0.001
365	AMW1657	2-Tridecanone	Aldehyde, Ketones, Esters	C13H26O	1.278	0.005
366	AMW2708	Phenol, 3,5-diethyl-	Benzene and substituted derivatives	C10H14O	1.509	0.000
367	AMW3314	Sulfurous acid, 2-ethylhexyl pentyl ester	Aldehyde, Ketones, Esters	C13H28O3S	1.101	0.009
368	AMW4441	5,11-Diethyl-8-methyl-7,9-dioxapentadecane	Hydrocarbons	C18H38O2	1.021	0.000
369	AMW2716	Hexadecane, 2,6,10-trimethyl-	Terpenoids	C19H40	1.215	0.000
370	AMW0056	Sulfurous acid, 2-ethylhexyl isohexyl ester	Organic acid and Its derivatives	C14H30O3S	1.192	0.002
371	AMW2574	Heptadecane, 4-methyl-	Hydrocarbons	C18H38	1.117	0.005
372	AMW2459	Pentadecane, 5-methyl-	Hydrocarbons	C16H34	1.225	0.004
373	AMW0483	4-(2,6,6-Trimethylcyclohexa-1,3-dienyl)but-3-en-2-one	Aldehyde, Ketones, Esters	C13H18O	1.007	0.001
374	AMW3829	Propanoic acid, 2,2-dimethyl-, 2,4-dinitrophenyl ester	Aldehyde, Ketones, Esters	C11H12N2	1.174	0.006

				O6		
375	AMW3788	4H-1,2,4-Triazol-3-amine, 4-propyl-	Alcohol and amines	C5H10N4	1.345	0.006
376	AMW2610	2(3H)-Furanone, 5-acetyldihydro-	Aldehyde, Ketones, Esters	C6H8O3	1.344	0.004
377	AMW3409	Piperoxan	Heterocyclic compounds	C14H19NO2	1.112	0.006
378	AMW0059	Sulfurous acid, 2-ethylhexyl hexyl ester	Aldehyde, Ketones, Esters	C14H30O3S	1.466	0.001
379	AMW0936	4(1H)-Pyrimidinone, 1-methyl-	Aldehyde, Ketones, Esters	C5H6N2O	1.519	0.000
380	AMW1660	Octadecane	Hydrocarbons	C18H38	1.118	0.003
381	AMW1185	Tridecane, 1-iodo-	Halogenated hydrocarbons	C13H27I	1.105	0.004
382	AMW3134	1,4-Benzenediamine, N,N-dimethyl-	Alcohol and amines	C8H12N2	1.519	0.000
383	AMW3375	2-Propenoic acid, (1-methyl-1,2-ethanediyl)bis[oxy(methyl-2,1-ethanediyl)] ester	Aldehyde, Ketones, Esters	C15H24O6	1.136	0.002
384	AMW1543	cis-9-Hexadecenal	Aldehyde, Ketones, Esters	C16H30O	1.147	0.002
385	AMW0669	Heptadecane, 2-methyl-	Hydrocarbons	C18H38	1.126	0.002
386	AMW1514	N-methyl-N-nitroso-1-Dodecanamine	Alcohol and amines	C13H28N2O	1.139	0.016
387	AMW4135	l-Proline, N-methoxycarbonyl-, isohexyl ester	Aldehyde, Ketones, Esters	C13H23NO4	1.094	0.005
388	AMW4210	Butyl 2-(2-(2-butoxyethoxy)ethoxy)acetate	Aldehyde, Ketones, Esters	C14H28O5	1.257	0.001
389	AMW0569	5-Fluoro-1,3-benzoxazol-2(3H)-one	Aldehyde, Ketones, Esters	C7H4FNO2	1.168	0.014
390	AMW2958	(2S,6R,7S,8E)-(+)-2,7-Epoxy-4,8-megastigmadiene	Heterocyclic compounds	C13H20O	1.284	0.001
391	AMW4423	2-Ethylbutyric acid, 2,6-dimethoxyphenyl ester	Aldehyde, Ketones, Esters	C14H20O4	1.192	0.001
392	AMW3594	Carbonic acid, bis(2-ethylhexyl) ester	Aldehyde, Ketones, Esters	C17H34O3	1.088	0.003
393	AMW1397	Hexadecane, 2,6,11,15-tetramethyl-	Hydrocarbons	C20H42	1.077	0.005
394	AMW0435	1-Octadecene	Hydrocarbons	C18H36	1.087	0.002
395	AMW0623	Azacyclohexane, 3-[1-pyrrolidyl]-	Heterocyclic compounds	C9H18N2	1.438	0.003
396	AMW4301	Benzyl alcohol, .alpha.-(1-(dimethylamino)ethyl)-	Alcohol and amines	C11H17NO	1.309	0.003

397	AMW4510	2-[2-(4-Methyl-furazan-3-yloxy)-ethyl]-2H-tetrazol-5-ylamine	Alcohol and amines	C6H9N7O2	1.187	0.038
398	AMW4478	5-.alpha.-Aminopropyltetrazole	Heterocyclic compounds	C4H9N5	1.046	0.050
399	AMW3775	Bicyclo[3.3.0]octan-3-one, 6-hydroxy-6-methyl-	Aldehyde, Ketones, Esters	C9H14O2	1.536	0.000
400	AMW3548	Tridecane, 6-propyl-	Hydrocarbons	C16H34	1.078	0.030
401	AMW3624	Cyclopentane, 1-butyl-2-ethyl-	Hydrocarbons	C11H22	1.176	0.000
402	AMW2936	3-Methyl-1H-pyrazole-4-carbaldehyde	Aldehyde, Ketones, Esters	C5H6N2O	1.181	0.002
403	AMW1705	Isopropyl phenyl ketone	Aldehyde, Ketones, Esters	C10H12O	1.038	0.000
404	AMW4047	3-Hydroxy-1-methylpyridinium hydroxide	Heterocyclic compounds	C6H9NO2	1.154	0.045
405	AMW3430	1,2,4-Triazine-3,5(2H,4H)-dione	Aldehyde, Ketones, Esters	C3H3N3O2	1.067	0.037
406	AMW1104	1-Piperidineethanol	Alcohol and amines	C7H15NO	1.243	0.001
407	AMW2387	Cyclohexene, 3-(1,5-dimethyl-4-hexenyl)-6-methylene-, [S-(R*,S*)]-	Terpenoids	C15H24	1.202	0.003
408	AMW3762	Cyclododecanol	Alcohol and amines	C12H24O	1.188	0.020
409	AMW0122	(2E)-6Acetoxy-2-methylhexenal	Aldehyde, Ketones, Esters	C9H14O3	1.235	0.007
410	AMW4299	cis-2,4-Dimethylthiane, S,S-dioxide	Heterocyclic compounds	C7H14O2S	1.228	0.002
411	AMW4783	Heptane, 4-ethyl-2,2,6,6-tetramethyl-	Hydrocarbons	C13H28	1.254	0.001
412	AMW4076	Resorcinol monoacetate	Aldehyde, Ketones, Esters	C8H8O3	1.172	0.005
413	AMW3710	l-Alanine, N-ethoxycarbonyl-, pentyl ester	Aldehyde, Ketones, Esters	C11H21NO4	1.137	0.017
414	AMW5090	N-Thio-valero-morpholine	Heterocyclic compounds	C9H17NOS	1.222	0.001
415	AMW0419	1-Undecanol	Alcohol and amines	C11H24O	1.030	0.035
416	AMW0453	1-Hexyl-1-nitrocyclohexane	Nitrogen compounds	C12H23NO2	1.196	0.007
417	AMW3026	3-Methylbut-2-enoic acid, 4-nitrophenyl ester	Aldehyde, Ketones, Esters	C11H11NO4	1.063	0.048
418	AMW1828	Hexadecanal	Aldehyde, Ketones, Esters	C16H32O	1.059	0.007
419	AMW1826	n-Pentadecanol	Alcohol and amines	C15H32O	1.298	0.001

420	AMW2245*110	Benzene, 1,2,4,5-tetramethyl-	Benzene and substituted derivatives	C10H14	1.303	0.000
421	AMW1744*107	2,4,6-trimethyl-Decane	Hydrocarbons	C13H28	1.379	0.000
422	AMW1223*071	2,3,4-Trimethylpyrrole	Heterocyclic compounds	C7H11N	1.217	0.000
423	AMW1501	3-(Ehtylthio)propanal	Aldehyde, Ketones, Esters	C5H10OS	1.381	0.000
424	AMW2123	Propanamide	Alcohol and amines	C3H7NO	1.528	0.000
425	AMW4132	Ethanone, 2-hydroxy-1-phenyl-	Aldehyde, Ketones, Esters	C8H8O2	1.234	0.000
426	AMW2690	4-Isopropylcyclohexylamine	Alcohol and amines	C9H19N	1.370	0.000
427	AMW0403	2-methyl-Cyclopentanone	Aldehyde, Ketones, Esters	C6H10O	1.418	0.000
428	AMW3714	Benzene, 1-ethyl-4-methoxy-	Ethers	C9H12O	1.539	0.000
429	AMW0633	Pyrazine, trimethyl-	Heterocyclic compounds	C7H10N2	1.140	0.003
430	AMW3167	(1-Propoxy-pentyl)-cyclopropane	Ethers	C11H22O	1.458	0.000
431	AMW2076	3-Hexen-2-one	Aldehyde, Ketones, Esters	C6H10O	1.375	0.000
432	AMW1684	Phenylethyl Alcohol	Alcohol and amines	C8H10O	1.541	0.000
433	AMW4728	Pyrazine, 2-ethyl-3-methyl-	Heterocyclic compounds	C7H10N2	1.423	0.001
434	AMW4786	2-Furaldehyde diethyl acetal	Aldehyde, Ketones, Esters	C9H14O3	1.169	0.009
435	AMW0736	ethyl-Cyclohexane	Hydrocarbons	C8H16	1.344	0.010
436	AMW3469	Amyl crotonate	Aldehyde, Ketones, Esters	C9H16O2	1.375	0.003
437	AMW3075	Glutaraldehyde	Aldehyde, Ketones, Esters	C5H8O2	1.434	0.000
438	AMW4119	Cyclohexanol, 1-ethenyl-	Alcohol and amines	C8H14O	1.427	0.001
439	AMW4738	2-Hexen-1-ol, acetate, (E)-	Aldehyde, Ketones, Esters	C8H14O2	1.353	0.003
440	AMW0726	Ethanol, 2-(diethylamino)-, N-oxide	Alcohol and amines	C6H15NO2	1.403	0.000
441	AMW1277	2-methyl-Benzofuran	Heterocyclic compounds	C9H8O	1.309	0.000
442	AMW2282*166	Carvone	Terpenoids	C10H14O	1.530	0.000
443	AMW4919*166	(-)-Carvone	Terpenoids	C10H14O	1.530	0.000
444	AMW0507*387	BenzAldehyde, 4-methoxy-	Aldehyde, Ketones, Esters	C8H8O2	1.523	0.000
445	AMW3044*339	trans-Ocimenol	Terpenoids	C10H18O	1.484	0.002

446	AMW1099*069	3,5-Octadien-2-one, (E,E)-	Aldehyde, Ketones, Esters	C8H12O	1.264	0.036
447	AMW4882*367	Decane, 2,5,6-trimethyl-	Hydrocarbons	C13H28	1.401	0.001
448	AMW4686*211	δ-valerolactone	Aldehyde, Ketones, Esters	C5H8O2	1.275	0.000
449	AMW2010*345	7-methyl-1-Undecene	Hydrocarbons	C12H24	1.493	0.001
450	AMW4862*345	4-Undecene, 5-methyl-, (E)-	Hydrocarbons	C12H24	1.493	0.001
451	AMW0234*125	2,6-Octadien-1-ol, 3,7-dimethyl-, (Z)-	Terpenoids	C10H18O	1.412	0.008
452	AMW4915*125	3,6-Octadien-1-ol, 3,7-dimethyl-, (Z)-	Terpenoids	C10H18O	1.412	0.008
453	AMW4481*263	(3Z,5E)-1,3,5-Undecatriene	Hydrocarbons	C11H18	1.469	0.002
454	AMW2383*338	Isoborneol	Terpenoids	C10H18O	1.459	0.003
455	AMW2520*338	Bicyclo[2.2.1]heptan-2-ol, 1,7,7-trimethyl-, (1S-endo)-	Terpenoids	C10H18O	1.459	0.003
456	AMW0705*347	5,6-dimethyl-Decane	Hydrocarbons	C12H26	1.345	0.016
457	AMW3333*107	Decane, 2,5,9-trimethyl-	Hydrocarbons	C13H28	1.379	0.000
458	AMW0311*236	Phenol, 3,5-dimethyl-	Benzene and substituted derivatives	C8H10O	1.214	0.003
459	AMW2607*359	1H-3a,7-Methanoazulene, octahydro-3,8,8-trimethyl-6-methylene-, [3R-(3.alpha.,3a.beta.,7.beta.,8a.alpha.)]-	Terpenoids	C15H24	1.501	0.001
460	AMW1741*068	2,4,6-trimethyl-Octane	Hydrocarbons	C11H24	1.194	0.000
461	AMW4924*366	Undecane, 2,3-dimethyl-	Hydrocarbons	C13H28	1.379	0.013
462	AMW1707*366	Dodecane, 4-methyl-	Hydrocarbons	C13H28	1.379	0.013
463	AMW3470*366	Decane, 5-propyl-	Hydrocarbons	C13H28	1.379	0.013
464	AMW2569*366	Dodecane, 2-methyl-	Hydrocarbons	C13H28	1.379	0.013
465	AMW2511*372	Nonane, 2,3-dimethyl-	Hydrocarbons	C11H24	1.320	0.000
466	AMW3273*326	2-Carene	Terpenoids	C10H16	1.084	0.036
467	AMW1080*326	(+)-4-Carene	Terpenoids	C10H16	1.084	0.036
468	AMW1757*117	3,3,6-trimethyl-Decane	Hydrocarbons	C13H28	1.462	0.003
469	AMW1742*117	2,6,7-trimethyl-Decane	Hydrocarbons	C13H28	1.462	0.003
470	AMW1756*117	3,3,5-trimethyl-Decane	Hydrocarbons	C13H28	1.462	0.003
471	AMW3150*117	Decane, 3,3,8-trimethyl-	Hydrocarbons	C13H28	1.462	0.003

472	AMW0760*335	3-Ethyl-3-methylheptane	Hydrocarbons	C10H22	1.303	0.000
473	AMW2159*122	6-methyl-5-Undecene	Hydrocarbons	C12H24	1.433	0.002
474	AMW2012*122	(Z)-4-methyl-4-Undecene	Hydrocarbons	C12H24	1.433	0.002
475	AMW4764*330	Cyclooctane, 1,4-dimethyl-, cis-	Hydrocarbons	C10H20	1.330	0.021
476	AMW0189*118	3-Nonen-1-ol, (Z)-	Alcohol and amines	C9H18O	1.349	0.000
477	AMW3385*351	valerolactone	Aldehyde, Ketones, Esters	C10H20O2	1.272	0.032
478	AMW2397*368	Undecane, 6-ethyl-	Hydrocarbons	C13H28	1.382	0.012
479	AMW2963*368	Undecane, 3,9-dimethyl-	Hydrocarbons	C13H28	1.382	0.012
480	AMW2726*368	Undecane, 4-ethyl-	Hydrocarbons	C13H28	1.382	0.012
481	AMW1546*111	Terpinen-4-ol	Terpenoids	C10H18O	1.390	0.000
482	AMW0778*369	Undecane, 5,7-dimethyl-	Hydrocarbons	C13H28	1.298	0.001
483	AMW4937*233	Fumaric Acid	Organic acid and Its derivatives	C4H4O4	1.357	0.000
484	AMW2908*207	4H-1,2,4-Triazol-3-amine, 4-methyl-	Alcohol and amines	C3H6N4	1.279	0.002
485	AMW0627*184	3-methyl-1H-Pyrazole	Heterocyclic compounds	C4H6N2	1.257	0.000
486	AMW0763*074	Nonane, 2,6-dimethyl-	Hydrocarbons	C11H24	1.501	0.000
487	AMW0764*074	Nonane, 3,7-dimethyl-	Hydrocarbons	C11H24	1.501	0.000
488	AMW1093*317	.gamma.-Muurolene	Terpenoids	C15H24	1.291	0.010
489	AMW2911*317	Naphthalene, 1,2,4a,5,8,8a-hexahydro-4,7-dimethyl-1-(1-methylethyl)-, (1.alpha.,4a.beta.,8a.alpha.)-(.+/-.)-	Terpenoids	C15H24	1.291	0.010
490	AMW2492*317	Naphthalene, 1,2,4a,5,6,8a-hexahydro-4,7-dimethyl-1-(1-methylethyl)-, (1.alpha.,4a.alpha.,8a.alpha.)-	Terpenoids	C15H24	1.291	0.010
491	AMW4890*252	Creosol	Benzene and substituted derivatives	C8H10O2	1.369	0.017
492	AMW4943*283	3-Tridecene, (Z)-	Hydrocarbons	C13H26	1.323	0.001
493	AMW0729*255	2,7-dimethyl-2,6-Octadiene	Hydrocarbons	C10H18	1.168	0.030
494	AMW4891*272	1-Octanol, 3,7-dimethyl-	Alcohol and amines	C10H22O	1.259	0.001
495	AMW1014*386	8-Heptadecene	Hydrocarbons	C17H34	1.220	0.037
496	AMW1700*132	Dodecane, 4,6-dimethyl-	Hydrocarbons	C14H30	1.290	0.001
497	AMW4955*257	Naphthalene, 1-methyl-	Benzene and substituted derivatives	C11H10	1.331	0.035

498	AMW1520*266	(+)-Dihydrocarvone	Terpenoids	C10H16O	1.291	0.003
499	AMW2523*293	Tridecane, 2,5-dimethyl-	Hydrocarbons	C15H32	1.271	0.002
500	AMW5170*151	9-Hexadecenoic acid	Organic acid and Its derivatives	C16H30O2	1.173	0.004
501	AMW1209*151	(Z)-hexadec-9-enoic acid	Organic acid and Its derivatives	C16H30O2	1.173	0.004
502	AMW0307*095	1,3-diethenyl-Benzene	Benzene and substituted derivatives	C10H10	1.233	0.003
503	AMW2695*259	3-Vinyl-1,2-dithiacyclohex-5-ene	Heterocyclic compounds	C6H8S2	1.370	0.006
504	AMW3353*222	1-(3H-Imidazol-4-yl)-ethanone	Heterocyclic compounds	C5H6N2O	1.415	0.035
505	AMW1436*161	(-)-.beta.-Bourbonene	Terpenoids	C15H24	1.512	0.001
506	AMW2191*337	piperitones	Terpenoids	C10H16O	1.507	0.000
507	AMW1444*110	Benzene, 1,2,3,5-tetramethyl-	Benzene and substituted derivatives	C10H14	1.303	0.000
508	AMW1364*110	1,2,3,4-tetramethyl-Benzene	Benzene and substituted derivatives	C10H14	1.303	0.000
509	AMW0920*071	2,3,5-trimethyl-1H-Pyrrole	Heterocyclic compounds	C7H11N	1.217	0.000

Note: VIP is Variable importance projection. P-value is significance test P-value.

Table S3 Annotated list of CK vs. L differential metabolites.

Index	Compounds	Class I	Formula	VIP	P	FC	Log2FC	Type
AMW0862*111	3-Cyclohexen-1-ol, 4-methyl-1-(1-methylethyl)-, (R)-	Terpenoids	C10H18O	1.442	0.000	0.456	-1.131	down
AMW1252*374	3-(2-methylpropyl)-Cyclohexene	Hydrocarbons	C10H18	1.183	0.056	0.303	-1.723	down
AMW4876	2-Heptenal, 2-propyl-	Aldehyde, Ketones, Esters	C10H18O	1.436	0.003	0.471	-1.087	down
AMW2164	2,6-Difluorobenzyl bromide	Others	C7H5BrF2	1.059	0.105	2.140	1.098	up
AMW1458	4-methyl-3-Pentenal	Aldehyde, Ketones, Esters	C6H10O	1.442	0.000	3.986	1.995	up
AMW0159	Benzaldehyde	Aldehyde, Ketones, Esters	C7H6O	1.431	0.000	2.223	1.152	up
AMW1462	3-ethyl-Pyridine	Heterocyclic compounds	C7H9N	1.446	0.000	2.823	1.497	up
AMW2862	Terpinyl formate	Aldehyde, Ketones, Esters	C11H18O2	1.443	0.000	0.441	-1.183	down
AMW0783	1H-Imidazole-2-methanol, 1-methyl-	Alcohol and amines	C5H8N2O	1.444	0.000	0.450	-1.154	down
AMW0252	2-Propenoic acid, oxiranylmethyl ester	Aldehyde, Ketones, Esters	C6H8O3	1.445	0.001	5.071	2.342	up
AMW2315	Heptylcyclohexane	Hydrocarbons	C13H26	1.392	0.030	2.178	1.123	up
AMW3047	Pentadecane, 6-methyl-	Hydrocarbons	C16H34	1.010	0.190	0.419	-1.255	down
AMW2401	Hydrazine, (phenylmethyl)-	Nitrogen compounds	C7H10N2	1.444	0.001	0.124	-3.011	down
AMW5018	Isosativene	Hydrocarbons	C15H24	1.435	0.005	0.461	-1.116	down
AMW5017	2-Butanone, 4-(2,6,6-trimethyl-1,3-cyclohexadien-1-yl)-	Aldehyde, Ketones, Esters	C13H20O	1.440	0.000	2.116	1.081	up
AMW2867	Heptane, 2,5,5-trimethyl-	Hydrocarbons	C10H22	1.429	0.002	2.897	1.534	up
AMW0320	Cyclohexanone	Aldehyde, Ketones, Esters	C6H10O	1.435	0.000	2.108	1.076	up
AMW2138	.alpha.-Terpinyl acetate	Aldehyde, Ketones, Esters	C12H20O2	1.431	0.008	2.147	1.103	up
AMW0502	Benzene acetaldehyde	Aldehyde, Ketones, Esters	C8H8O	1.436	0.000	0.287	-1.802	down
AMW2708	Phenol, 3,5-diethyl-	Benzene and substituted derivatives	C10H14O	1.431	0.002	2.365	1.242	up
AMW3631	1H-[1,2,4]Triazolo[4,3-b][1,2,4]triazole-3,6-diamine	Alcohol and amines	C3H5N7	1.364	0.004	0.457	-1.131	down
AMW0936	4(1H)-Pyrimidinone, 1-methyl-	Aldehyde, Ketones, Esters	C5H6N2O	1.428	0.011	2.164	1.113	up
AMW3134	1,4-Benzenediamine, N,N-dimethyl-	Alcohol and amines	C8H12N2	1.421	0.004	2.032	1.023	up
AMW2958	(2S,6R,7S,8E)-(+)-2,7-Epoxy-4,8-megastigmadiene	Heterocyclic compounds	C13H20O	1.413	0.001	2.097	1.068	up

AMW3775	Bicyclo[3.3.0]octan-3-one, 6-hydroxy-6-methyl-	Aldehyde, Ketones, Esters	C9H14O2	1.439	0.006	3.682	1.881	up
AMW3624	Cyclopentane, 1-butyl-2-ethyl-	Hydrocarbons	C11H22	1.272	0.005	0.169	-2.561	down
AMW1769	Propanoic acid, pentyl ester	Aldehyde, Ketones, Esters	C8H16O2	1.407	0.001	0.461	-1.117	down
AMW2188*374	(2-methyl-1-propenyl)-Cyclohexane	Hydrocarbons	C10H18	1.183	0.056	0.303	-1.723	down
AMW1947*179	cis-2-(2-Pentenyl)furan	Heterocyclic compounds	C9H12O	1.441	0.000	0.138	-2.859	down
AMW2534	1,2,4,5-Tetrazine	Heterocyclic compounds	C2H2N4	1.446	0.000	8.184	3.033	up
AMW1501	3-(Ehtylthio)propanal	Aldehyde, Ketones, Esters	C5H10OS	1.446	0.000	0.175	-2.518	down
AMW2123	Propanamide	Alcohol and amines	C3H7NO	1.420	0.001	2.612	1.385	up
AMW3714	Benzene, 1-ethyl-4-methoxy-	Ethers	C9H12O	1.445	0.001	0.190	-2.393	down
AMW1684	Phenylethyl Alcohol	Alcohol and amines	C8H10O	1.445	0.003	0.188	-2.413	down
AMW2142	3-methyl-3-Buten-2-one	Aldehyde, Ketones, Esters	C5H8O	1.446	0.001	5.361	2.422	up
AMW3075	Glutaraldehyde	Aldehyde, Ketones, Esters	C5H8O2	1.161	0.014	4.580	2.195	up
AMW4119	Cyclohexanol, 1-ethenyl-	Alcohol and amines	C8H14O	1.099	0.022	2.780	1.475	up
AMW4738	2-Hexen-1-ol, acetate, (E)-	Aldehyde, Ketones, Esters	C8H14O2	1.147	0.054	3.152	1.656	up
AMW0117	Hexadecyl pentyl ether	Ethers	C21H44O	1.441	0.006	0.109	-3.197	down
AMW0726	Ethanol, 2-(diethylamino)-, N-oxide	Alcohol and amines	C6H15NO2	1.442	0.007	5.825	2.542	up
AMW1546*111	Terpinen-4-ol	Terpenoids	C10H18O	1.442	0.000	0.456	-1.131	down
AMW0892*179	Furan, 2-(1-pentenyl)-, (E)-	Heterocyclic compounds	C9H12O	1.441	0.000	0.138	-2.859	down
AMW4724*179	trans-2-(2-Pentenyl)furan	Heterocyclic compounds	C9H12O	1.441	0.000	0.138	-2.859	down

Note: FC is Fold Change. type is metabolite up- and down-regulation type.

Table S4 Annotated list of CK vs. Y differential metabolites.

Index	Compounds	Class I	VIP	P	FC	Log_2FC	Type
AMW0862*111	3-Cyclohexen-1-ol, 4-methyl-1-(1-methylethyl)-, (R)-	Terpenoids	1.317	0.000	0.464	-1.109	down
AMW3081*352	n-Amyl isovalerate	Aldehyde, Ketones, Esters	1.265	0.003	2.390	1.257	up
AMW2965*207	1H-1,2,4-Triazol-3-amine, 5-methyl-	Alcohol and amines	1.194	0.015	2.438	1.285	up
AMW0283*132	3,5-Dimethyldodecane	Hydrocarbons	1.207	0.023	2.146	1.102	up
AMW2201*257	Naphthalene, 2-methyl-	Benzene and substituted derivatives	1.293	0.033	2.726	1.447	up
AMW2378*293	Tridecane, 4,8-dimethyl-	Hydrocarbons	1.181	0.021	2.287	1.193	up
AMW3359*352	Butanoic acid, 3-methyl-, 3-methylbutyl ester	Aldehyde, Ketones, Esters	1.265	0.003	2.390	1.257	up
AMW1002*222	Ethanone, 1-(1H-pyrazol-4-yl)-	Aldehyde, Ketones, Esters	1.205	0.108	2.273	1.184	up
AMW4465	N-Cyclopropanecarbonylcyclopropanecarbohydrazide	Nitrogen compounds	1.191	0.017	3.117	1.640	up
AMW0405	2-Undecanone	Aldehyde, Ketones, Esters	1.320	0.000	0.421	-1.247	down
AMW3007	Oxalic acid, butyl cyclobutyl ester	Aldehyde, Ketones, Esters	1.187	0.016	2.482	1.311	up
AMW1302	2-Phenylpropenal	Aldehyde, Ketones, Esters	1.323	0.001	5.471	2.452	up
AMW2803	Pentanoic acid, 2-methyl-, anhydride	Organic acid and Its derivatives	1.230	0.007	2.793	1.482	up
AMW3142	Nonane, 5-propyl-	Hydrocarbons	1.247	0.013	2.268	1.181	up
AMW0277	Phenylglyoxal	Aldehyde, Ketones, Esters	1.321	0.001	0.411	-1.283	down
AMW2364	4-Methylpentyl 4-methylpentanoate	Aldehyde, Ketones, Esters	1.294	0.001	2.233	1.159	up
AMW0159	BenzAldehyde	Aldehyde, Ketones, Esters	1.321	0.000	4.394	2.135	up
AMW4949	Phenol, p-tert-butyl-	Benzene and substituted derivatives	1.159	0.025	3.807	1.929	up
AMW0102	Ethyl 3-methylbut-3-enyl carbonate	Aldehyde, Ketones, Esters	1.323	0.000	0.356	-1.491	down
AMW3715	1-Methyl-2,4,5-trioxoimidazolidine	Heterocyclic compounds	1.221	0.019	2.006	1.004	up
AMW3148	2-Cyano-2-isopropyl-3-methylbutanoic acid	Organic acid and Its derivatives	1.232	0.014	2.572	1.363	up
AMW3347	Decane, 3-bromo-	Halogenated hydrocarbons	1.224	0.009	4.198	2.070	up
AMW4049	(3R)-(+)-3-Acetamidopyrrolidine	Heterocyclic compounds	1.265	0.004	5.805	2.537	up
AMW3041	5-Methylenehydantoin	Nitrogen compounds	1.257	0.009	2.634	1.397	up

AMW2323	n-Caprylic acid isobutyl ester	Aldehyde, Ketones, Esters	1.229	0.013	2.451	1.293	up
AMW2938	Tridecane, 7-methyl-	Hydrocarbons	1.219	0.010	2.598	1.377	up
AMW1184	4(1H)-Pyridinone, 2,3-dihydro-1-methyl-	Aldehyde, Ketones, Esters	1.309	0.000	2.254	1.172	up
AMW3250	4-Octen-3-one, 6-ethyl-7-hydroxy-	Aldehyde, Ketones, Esters	1.248	0.011	2.023	1.017	up
AMW4125	Butanedioic acid, 2-hydroxy-2-methyl-, (S)-	Organic acid and Its derivatives	1.234	0.014	2.065	1.046	up
AMW0717	2-Undecanol	Alcohol and amines	1.164	0.044	2.041	1.029	up
AMW2902	1H-1,2,3-Triazole-4-carboxaldehyde	Aldehyde, Ketones, Esters	1.163	0.019	4.708	2.235	up
AMW2517	exo-2-Hydroxycineole acetate	Aldehyde, Ketones, Esters	1.125	0.052	2.407	1.267	up
AMW3303	Decane, 2,3,5,8-tetramethyl-	Hydrocarbons	1.224	0.002	5.919	2.565	up
AMW0490	2-Imidazolidinone	Aldehyde, Ketones, Esters	1.192	0.028	2.387	1.255	up
AMW4946	Pentanedioic acid	Organic acid and Its derivatives	1.081	0.128	3.128	1.645	up
AMW2120	(2,2,6-Trimethyl-bicyclo[4.1.0]hept-1-yl)-methanol	Alcohol and amines	1.185	0.021	3.614	1.854	up
AMW1462	3-ethyl-Pyridine	Heterocyclic compounds	1.322	0.000	11.224	3.488	up
AMW0819	N-(n-Butoxymethyl)acrylamide	Alcohol and amines	1.253	0.009	2.103	1.072	up
AMW3629	Ethanone, 1-(1-methylcyclohexyl)-	Aldehyde, Ketones, Esters	1.238	0.006	3.446	1.785	up
AMW3214	4-Heptanone, 2,2,3,3,5,5,6,6-octamethyl-	Aldehyde, Ketones, Esters	1.191	0.018	2.988	1.579	up
AMW0103	Ethyl 2-(5-methyl-5-vinyltetrahydrofuran-2-yl)propan-2-yl carbonate	Aldehyde, Ketones, Esters	1.184	0.028	2.103	1.072	up
AMW4976	6-Dodecanone	Aldehyde, Ketones, Esters	1.186	0.016	3.626	1.858	up
AMW5072	3-Hydroxy-4-methoxybenzoic acid	Organic acid and Its derivatives	1.262	0.006	2.733	1.451	up
AMW0451	4-[pyrrolidin-2-one-5-yl]-Butan-2-one	Aldehyde, Ketones, Esters	1.107	0.061	2.496	1.320	up
AMW2413	Tridecane, 5-methyl-	Hydrocarbons	1.219	0.018	2.844	1.508	up
AMW0783	1H-Imidazole-2-methanol, 1-methyl-	Alcohol and amines	1.319	0.000	0.482	-1.053	down
AMW1273	2-methoxy-6-methyl-4H-Pyran-4-one	Aldehyde, Ketones, Esters	1.314	0.001	2.561	1.357	up
AMW0252	2-Propenoic acid, oxiranylmethyl ester	Aldehyde, Ketones, Esters	1.309	0.000	2.514	1.330	up
AMW3755	dl-2-Aminobutyric acid	Organic acid and Its derivatives	1.316	0.001	0.485	-1.044	down
AMW1292	9-Azabicyclo[3.3.1]nonan-3-one	Aldehyde, Ketones, Esters	1.075	0.075	2.601	1.379	up

AMW2027	(E)-2-Tridecen-1-ol	Hydrocarbons	1.228	0.014	2.332	1.222	up
AMW3898	Thiophene-2-acetic acid, 2-dimethylaminoethyl ester	Aldehyde, Ketones, Esters	1.173	0.038	2.207	1.142	up
AMW3149	Diethanolamine	Alcohol and amines	1.247	0.006	2.902	1.537	up
AMW3773	3-Isopropyl-6,10-dimethylundecane-2-ol	Alcohol and amines	1.209	0.067	0.473	-1.081	down
AMW3746	Sulfurous acid, isobutyl pentyl ester	Aldehyde, Ketones, Esters	1.190	0.016	2.422	1.276	up
AMW2272	5-Ethyl-5-(propan-2-yl)imidazolidine-2,4-dione	Aldehyde, Ketones, Esters	1.231	0.006	2.492	1.317	up
AMW2920	Pentadecane, 7-methyl-	Hydrocarbons	1.230	0.016	2.221	1.151	up
AMW0055	Sulfurous acid, 2-ethylhexyl isobutyl ester	Organic acid and Its derivatives	1.160	0.020	2.454	1.295	up
AMW3047	Pentadecane, 6-methyl-	Hydrocarbons	1.229	0.019	2.130	1.091	up
AMW5069	2,2-Dimethylpropionic acid, decyl ester	Aldehyde, Ketones, Esters	1.198	0.019	2.676	1.420	up
AMW1140	.delta.-Nonalactone	Aldehyde, Ketones, Esters	1.091	0.044	2.349	1.232	up
AMW1822	Tetradecane	Hydrocarbons	1.137	0.038	2.063	1.045	up
AMW0399	1-Tetradecene	Hydrocarbons	1.146	0.033	2.155	1.107	up
AMW0394	Ethanol, 2-(2-ethoxyethoxy)-	Alcohol and amines	1.218	0.025	2.201	1.138	up
AMW2401	Hydrazine, (phenylmethyl)-	Nitrogen compounds	1.321	0.000	0.012	-6.329	down
AMW3765	3(2H)-Furanone, 2-(1-hydroxy-1-methyl-2-oxopropyl)-2,5-dimethyl-	Aldehyde, Ketones, Esters	1.148	0.031	2.164	1.113	up
AMW4996	4-Piperidinecarboxamide	Alcohol and amines	1.134	0.028	2.806	1.488	up
AMW4953	Butanedioic acid, methylene-	Organic acid and Its derivatives	1.212	0.018	3.348	1.743	up
AMW0537	Tetradecanal	Aldehyde, Ketones, Esters	1.141	0.025	2.717	1.442	up
AMW3574	Oxalic acid, cyclobutyl hexyl ester	Aldehyde, Ketones, Esters	1.147	0.025	2.601	1.379	up
AMW5089	1-Iodo-2-methylundecane	Halogenated hydrocarbons	1.143	0.025	2.672	1.418	up
AMW4442	Cyclohexanecarboxylic acid, 1-amino-	Organic acid and Its derivatives	1.151	0.029	2.470	1.305	up
AMW2363	6-Octenal, 7-methyl-3-methylene-	Terpenoids	1.271	0.001	2.618	1.388	up
AMW3756	2,5-Piperazinedione, 3-methyl-	Aldehyde, Ketones, Esters	1.164	0.020	2.592	1.374	up
AMW0487	(E)-Hex-3-enyl (E)-2-methylbut-2-enoate	Aldehyde, Ketones, Esters	1.156	0.013	4.532	2.180	up
AMW4337	4-Amino-3,5-dimethyl-1,2,4-triazole	Heterocyclic compounds	1.175	0.022	2.334	1.223	up

AMW0582	6,7-Dodecanedione	Aldehyde, Ketones, Esters	1.183	0.019	2.382	1.252	up
AMW4334	Oxalic acid, cyclohexyl ethyl ester	Aldehyde, Ketones, Esters	1.176	0.022	2.301	1.202	up
AMW2873	3,5-Diamino-1,2,4-triazole	Heterocyclic compounds	1.184	0.011	2.825	1.498	up
AMW4961	Bicyclo[2.2.1]heptane-2,3-dione, 1,7,7-trimethyl-, (1S)-	Aldehyde, Ketones, Esters	1.236	0.010	3.533	1.821	up
AMW2867	Heptane, 2,5,5-trimethyl-	Hydrocarbons	1.303	0.001	4.305	2.106	up
AMW0320	Cyclohexanone	Aldehyde, Ketones, Esters	1.317	0.000	6.526	2.706	up
AMW2138	.alpha.-Terpinyl acetate	Aldehyde, Ketones, Esters	1.314	0.004	3.231	1.692	up
AMW2948	Tetradecane, 4-ethyl-	Hydrocarbons	1.204	0.019	2.981	1.576	up
AMW2708	Phenol, 3,5-diethyl-	Benzene and substituted derivatives	1.316	0.004	3.031	1.600	up
AMW2459	Pentadecane, 5-methyl-	Hydrocarbons	1.147	0.031	2.275	1.186	up
AMW0936	4(1H)-Pyrimidinone, 1-methyl-	Aldehyde, Ketones, Esters	1.275	0.001	3.604	1.850	up
AMW1660	Octadecane	Hydrocarbons	1.147	0.027	2.468	1.304	up
AMW3134	1,4-Benzenediamine, N,N-dimethyl-	Alcohol and amines	1.304	0.001	2.681	1.423	up
AMW2958	(2S,6R,7S,8E)-(+)-2,7-Epoxy-4,8-megastigmadiene	Heterocyclic compounds	1.210	0.008	2.026	1.018	up
AMW1397	Hexadecane, 2,6,11,15-tetramethyl-	Hydrocarbons	1.139	0.030	2.548	1.349	up
AMW3231	1,15-Hexadecadiene	Hydrocarbons	1.028	0.137	0.420	-1.251	down
AMW3775	Bicyclo[3.3.0]octan-3-one, 6-hydroxy-6-methyl-	Aldehyde, Ketones, Esters	1.319	0.004	5.993	2.583	up
AMW3624	Cyclopentane, 1-butyl-2-ethyl-	Hydrocarbons	1.133	0.009	0.227	-2.137	down
AMW2936	3-Methyl-1H-pyrazole-4-carbaldehyde	Aldehyde, Ketones, Esters	1.035	0.012	3.269	1.709	up
AMW1705	Isopropyl phenyl ketone	Aldehyde, Ketones, Esters	1.161	0.008	5.621	2.491	up
AMW4047	3-Hydroxy-1-methylpyridinium hydroxide	Heterocyclic compounds	1.028	0.097	2.919	1.546	up
AMW3430	1,2,4-Triazine-3,5(2H,4H)-dione	Aldehyde, Ketones, Esters	1.019	0.082	2.659	1.411	up
AMW1104	1-Piperidineethanol	Alcohol and amines	1.144	0.024	4.340	2.118	up
AMW2387	Cyclohexene, 3-(1,5-dimethyl-4-hexenyl)-6-methylene-, [S-(R*,S*)]-	Terpenoids	1.122	0.033	3.820	1.933	up
AMW3762	Cyclododecanol	Alcohol and amines	1.106	0.027	3.919	1.970	up
AMW0122	(2E)-6Acetoxy-2-methylhexenal	Aldehyde, Ketones, Esters	1.096	0.050	3.385	1.759	up

AMW4299	cis-2,4-Dimethylthiane, S,S-dioxide	Heterocyclic compounds	1.129	0.034	3.979	1.992	up
AMW4783	Heptane, 4-ethyl-2,2,6,6-tetramethyl-	Hydrocarbons	1.152	0.018	4.557	2.188	up
AMW4076	Resorcinol monoacetate	Aldehyde, Ketones, Esters	1.095	0.042	3.388	1.760	up
AMW3710	l-Alanine, N-ethoxycarbonyl-, pentyl ester	Aldehyde, Ketones, Esters	1.052	0.070	2.882	1.527	up
AMW5090	N-Thio-valero-morpholine	Heterocyclic compounds	1.166	0.008	5.031	2.331	up
AMW0453	1-Hexyl-1-nitrocyclohexane	Nitrogen compounds	1.095	0.039	3.405	1.768	up
AMW3026	3-Methylbut-2-enoic acid, 4-nitrophenyl ester	Aldehyde, Ketones, Esters	1.050	0.051	3.095	1.630	up
AMW1828	Hexadecanal	Aldehyde, Ketones, Esters	1.057	0.066	2.926	1.549	up
AMW1826	n-Pentadecanol	Alcohol and amines	1.165	0.009	4.999	2.322	up
AMW2245*110	Benzene, 1,2,4,5-tetramethyl-	Benzene and substituted derivatives	1.322	0.001	5.287	2.402	up
AMW1223*071	2,3,4-Trimethylpyrrole	Heterocyclic compounds	1.322	0.000	5.060	2.339	up
AMW1947*179	cis-2-(2-Pentenyl)furan	Heterocyclic compounds	1.314	0.011	5.651	2.498	up
AMW5083	1-Penten-3-one, 1-(2,6,6-trimethyl-1-cyclohexen-1-yl)-	Terpenoids	1.020	0.113	0.392	-1.351	down
AMW1501	3-(Ehtylthio)propanal	Aldehyde, Ketones, Esters	1.321	0.001	0.186	-2.424	down
AMW2123	Propanamide	Alcohol and amines	1.320	0.002	14.599	3.868	up
AMW4132	Ethanone, 2-hydroxy-1-phenyl-	Aldehyde, Ketones, Esters	1.323	0.002	5.512	2.463	up
AMW2690	4-Isopropylcyclohexylamine	Alcohol and amines	1.323	0.000	6.157	2.622	up
AMW0403	2-methyl-Cyclopentanone	Aldehyde, Ketones, Esters	1.322	0.001	7.656	2.937	up
AMW3714	Benzene, 1-ethyl-4-methoxy-	Ethers	1.322	0.000	0.012	-6.345	down
AMW0633	Pyrazine, trimethyl-	Heterocyclic compounds	1.274	0.079	7.270	2.862	up
AMW3167	(1-Propoxy-pentyl)-cyclopropane	Ethers	1.322	0.001	8.435	3.076	up
AMW2076	3-Hexen-2-one	Aldehyde, Ketones, Esters	1.321	0.000	6.886	2.784	up
AMW1684	Phenylethyl Alcohol	Alcohol and amines	1.322	0.000	0.013	-6.276	down
AMW4728	Pyrazine, 2-ethyl-3-methyl-	Heterocyclic compounds	1.312	0.025	9.289	3.215	up
AMW4786	2-Furaldehyde diethyl acetal	Aldehyde, Ketones, Esters	1.289	0.072	9.030	3.175	up
AMW0883	1,11-Dodecadiyne	Hydrocarbons	1.013	0.099	0.363	-1.463	down

AMW0736	ethyl-Cyclohexane	Hydrocarbons	1.323	0.001	6.138	2.618	up
AMW3469	Amyl crotonate	Aldehyde, Ketones, Esters	1.322	0.002	7.950	2.991	up
AMW3075	Glutaraldehyde	Aldehyde, Ketones, Esters	1.321	0.001	25.633	4.680	up
AMW4119	Cyclohexanol, 1-ethenyl-	Alcohol and amines	1.316	0.007	10.378	3.375	up
AMW4738	2-Hexen-1-ol, acetate, (E)-	Aldehyde, Ketones, Esters	1.318	0.004	7.355	2.879	up
AMW0726	Ethanol, 2-(diethylamino)-, N-oxide	Alcohol and amines	1.320	0.007	5.825	2.542	up
AMW1277	2-methyl-Benzofuran	Heterocyclic compounds	1.322	0.000	0.120	-3.055	down
AMW1546*111	Terpinen-4-ol	Terpenoids	1.317	0.000	0.464	-1.109	down
AMW2908*207	4H-1,2,4-Triazol-3-amine, 4-methyl-	Alcohol and amines	1.194	0.015	2.438	1.285	up
AMW1700*132	Dodecane, 4,6-dimethyl-	Hydrocarbons	1.207	0.023	2.146	1.102	up
AMW4955*257	Naphthalene, 1-methyl-	Benzene and substituted derivatives	1.293	0.033	2.726	1.447	up
AMW2523*293	Tridecane, 2,5-dimethyl-	Hydrocarbons	1.181	0.021	2.287	1.193	up
AMW3353*222	1-(3H-Imidazol-4-yl)-ethanone	Heterocyclic compounds	1.205	0.108	2.273	1.184	up
AMW1444*110	Benzene, 1,2,3,5-tetramethyl-	Benzene and substituted derivatives	1.322	0.001	5.287	2.402	up
AMW1364*110	1,2,3,4-tetramethyl-Benzene	Benzene and substituted derivatives	1.322	0.001	5.287	2.402	up
AMW0920*071	2,3,5-trimethyl-1H-Pyrrole	Heterocyclic compounds	1.322	0.000	5.060	2.339	up
AMW0892*179	Furan, 2-(1-pentenyl)-, (E)-	Heterocyclic compounds	1.314	0.011	5.651	2.498	up
AMW4724*179	trans-2-(2-Pentenyl)furan	Heterocyclic compounds	1.314	0.011	5.651	2.498	up
